# Living apart together: crosstalk between the core and supernumerary genomes in a fungal plant pathogen

**DOI:** 10.1186/s12864-016-2941-6

**Published:** 2016-08-23

**Authors:** Adriaan Vanheule, Kris Audenaert, Sven Warris, Henri van de Geest, Elio Schijlen, Monica Höfte, Sarah De Saeger, Geert Haesaert, Cees Waalwijk, Theo van der Lee

**Affiliations:** 1 Department of Applied Biosciences, Faculty of Bioscience Engineering, Ghent University, Ghent, Belgium; 2 Wageningen UR, Wageningen, The Netherlands; 3 Department of Crop Protection, Faculty of Bioscience Engineering, Ghent University, Ghent, Belgium; 4 Department of Bioanalysis, Faculty of Pharmaceutical Sciences, Ghent University, Ghent, Belgium

**Keywords:** Single-molecule real-time sequencing, Supernumerary chromosomes, *Fusarium*, Repeat-induced point mutation, Translocation, Gene duplications, Transposable elements

## Abstract

**Background:**

Eukaryotes display remarkable genome plasticity, which can include supernumerary chromosomes that differ markedly from the core chromosomes. Despite the widespread occurrence of supernumerary chromosomes in fungi, their origin, relation to the core genome and the reason for their divergent characteristics are still largely unknown. The complexity of genome assembly due to the presence of repetitive DNA partially accounts for this.

**Results:**

Here we use single-molecule real-time (SMRT) sequencing to assemble the genome of a prominent fungal wheat pathogen, *Fusarium poae*, including at least one supernumerary chromosome. The core genome contains limited transposable elements (TEs) and no gene duplications, while the supernumerary genome holds up to 25 % TEs and multiple gene duplications. The core genome shows all hallmarks of repeat-induced point mutation (RIP), a defense mechanism against TEs, specific for fungi. The absence of RIP on the supernumerary genome accounts for the differences between the two (sub)genomes, and results in a functional crosstalk between them. The supernumerary genome is a reservoir for TEs that migrate to the core genome, and even large blocks of supernumerary sequence (>200 kb) have recently translocated to the core. Vice versa, the supernumerary genome acts as a refuge for genes that are duplicated from the core genome.

**Conclusions:**

For the first time, a mechanism was determined that explains the differences that exist between the core and supernumerary genome in fungi. Different biology rather than origin was shown to be responsible. A “living apart together” crosstalk exists between the core and supernumerary genome, accelerating chromosomal and organismal evolution.

**Electronic supplementary material:**

The online version of this article (doi:10.1186/s12864-016-2941-6) contains supplementary material, which is available to authorized users.

## Background

Genome plasticity is one of the most important drivers of evolutions in eukaryotes. This plasticity includes large scale genome duplications, rearrangements, deletions and compartmentalization [1–3]. Fungi exhibit these dynamics better than any other kingdom, possible due to their occurrence in highly diverse niches. The organization of fungal genomes varies remarkably and in many cases facilitates rapid evolution and speciation [4]. Indeed, the division of the genome into a core and accessory part evolving at different speeds, has been described in multiple pathogens, particularly fungi [5–7]. Supernumerary chromosomes are one of most radical extensions of genome plasticity in fungi. They represent chromosomal structures that vary in size and distribution among individuals of the same species and show presence/absence polymorphism [2]. Recent reviews have noted on the exceptional genome plasticity and particularly the widespread presence of supernumerary chromosomes as a hallmark of pathogenic fungi [6, 8]. For this reason, they represent excellent model organisms to investigate eukaryotic genome evolution [7].

In some cases, the supernumerary chromosomes contain genes involved in pathogenicity and/or efficient host colonization like in *Alternaria alternata* [9], *Fusarium solani* (formerly known as *Nectria haematococca*) [10], and *F. oxysporum* f. sp. *lycopersici* [11]. In other species, the role of supernumerary chromosomes is less clear as they do not show obvious pathogenicity related functions, such as *Zymoseptoria tritici* (formerly known as *Mycosphaerella graminicola*) [12]. Regardless, in all cases described up to now supernumerary chromosomes differ markedly from the core chromosomes in characteristics such as gene content, codon usage and distribution of transposable elements.

The specific reasons for the these differences are unclear. A popular hypothesis proposes a different evolutionary origin for supernumerary chromosomes, that are subsequently acquired by horizontal chromosome transfer [11, 13]. Different evolutionary pressure on supernumerary chromosomes has also been proposed as an explanation for the detected differences [5, 6]. It has been argued that supernumerary chromosomes represent extreme cases of genome compartmentalization, as was demonstrated within the core chromosomes of *Fusarium graminearum* and *Leptosphaeria maculans* [14, 15]. These compartments may serve as evolutionary cradles, enriched in genes such as secondary metabolite clusters, often transcriptionally silent and only expressed under specific conditions. In accordance, degeneration from the core genome has been proposed as a potential origin of supernumerary chromosomes [8].

Transposable elements (TE) play an important role in fungal genome diversity and the evolutionary success of some pathogens [2, 16]. Examples are the vast differences in genome sizes of *Fusarium* and *Phytophthora* species [11, 17], and the shaping of pathogenicity in *L. maculans* and *Pyrenophora tritici-repentis* [15, 18]. The link between TE-mediated genome expansion and the evolution of virulence factors has been reviewed extensively [16]. The possible deleterious effects of mobilization of TEs include gene disruption and intra- or inter-element recombination, potentially leading to gene loss. Fungi have evolved a specific genome defense mechanism against repetitive DNA, repeat-induced point mutation (RIP), that efficiently inactivates TEs by introducing cytosine to thymidine mutations [19]. However, this process does not discriminate between TE proliferation and gene duplications, and therefore the near-absence of paralogs has been found to be a hallmark of a RIP-active species, e.g. in *Fusarium graminearum* [20] and *Neurospora crassa* [21]. This finding has been termed the evolutionary cost of genome defense [22]. RIP functions on repetitive sequences with greater than 80 % identity and exceeding +/- 800 bp in length [22].

The RIP process occurs only during the di-karyotic pre-meiotic phase and is therefore intricately associated with sexual cycle [23]. Meiosis in fungi is partially regulated by the genes occupying the mating type locus. In heterothallic fungal species the locus is occupied by either the MAT1-1 or the MAT1-2 idiomorph, and isolates of these species require partners of the opposing mating type to enter into meiosis. For many species, a “cryptic” sexual cycle is presumed to occur in the field, that has never been witnessed nor simulated in the lab [24]. The presumption of active meiosis becomes substantiated when markers for a sexual lifestyle are considered, including recombination [25], RIP [26], distributions of the mating idiomorphs in the population [27], and functional constraint on the genes implicated for meiosis [28].

Meiosis is one of the drivers of diversity in length and number of supernumerary chromosomes in fungi. It has been shown that during meiosis a process called nondisjunction is responsible for the loss of these chromosomes in *Z. tritici* offspring, even if both parents contained the supernumerary chromosome [29]. Importantly, the offspring of these crosses are viable, underlining the conditionally dispensable nature of this part of the genome. The birth of a new supernumerary chromosome has been experimentally shown to occur through fusion of sister chromatids during meiosis, followed by breakage-fusion-bridge cycles [30]. How the presence of supernumerary chromosomes influences the fate of the core chromosomes and whether a crosstalk between the two genome complements exists, has not been investigated.

The genus *Fusarium* comprises many agriculturally and medically important pathogens [31]. As described above, species of this genus contain the hallmarks of fungal genome plasticity such as supernumerary chromosomes and compartmentalization of the core chromosomes. The *Fusarium* Head Blight disease on wheat and other small-grain cereals is caused by a number of species often co-occurring on the ear. Within this complex, *Fusarium poae* has been increasingly detected in a number of countries [32, 33]. Individuals of this species were shown to contain a highly variable set of supernumerary chromosomes [34]. The genome of the related species *F. graminearum* is one of the best assembled fungal genomes and therefore represents an excellent model for comparative genome biology studies, moreover it lacks supernumerary chromosomes [35]. In this study, we used SMRT sequencing to provide a high quality genome assembly of *F. poae*, and by a comparison with *F. graminearum* the cause of the sharp differences between the core and supernumerary genome was determined. This has led to a genetic crosstalk between the core and supernumerary genome, and the role of supernumerary chromosomes as evolutionary cradles that accelerate chromosomal and organismal evolution in fungi, was reaffirmed.

## Results

### The genome is composed of a core and supernumerary part

The genome of *F. poae* isolate 2516 was assembled from the single-molecule real-time (SMRT) reads using a combined approach, based on macrosynteny with related *Fusarium* species as well as support from different assemblies using different parameters. In this combined approach, the largest contigs from one SMRT assembly (assembly A) were queried against two SMRT assemblies based on different parameters (see Methods). By detecting long collinear stretches at the edges of these contigs, they could be manually merged to longer contiguous sequences and finally to chromosomes. Correct merging of contigs was verified by inspecting the mapping of SMRT reads. Finally a merged assembly of 4 chromosomes was obtained, originating from 9 contigs (two, three, three and one respectively), accounting for a total 38.13 Mb of sequence. The merged assembly of 4 chromosomes was supplemented with the remaining contigs and the degenerate unitigs from assembly A, and only the 172 non-redundant contigs were kept (see Methods).

The general statistics of this final assembly can be found in Table 1, and are compared with a *de novo* assembly of the HiSeq reads for this isolate. The SMRT assembly is larger by 7.28 Mb, significantly reduces the total number of contigs and has a much larger representation of bases in large contigs (N50 of 8783590 bp versus 170721 bp). The 4 merged chromosomes contain one, two, two and one telomeres, respectively. The long arm of chromosome 1 misses the telomere in this assembly, while the short arm of chromosome 4 ends in the ribosomal DNA tandem repeat. The long arm of chromosome 3 contains a 5000 N placeholder at 150 kb from the telomere. At this junction a 150 kb contig was joined to the rest of the assembly on the basis of its collinearity with *F. graminearum* and other *F. poae* isolates.

**Table 1:** Comparison of the SMRT and HiSeq assemblies of isolate 2516. The statistics for the SMRT assembly were extracted from the final version of the assembly: four core chromosomes and 172 supernumerary contigs

	SMRT assembly	HiSeq assembly
Number of contigs	176	1253
Average coverage	20.2	111.5
Total sequence length (bp)	46309701	39020932
Average sequence length (bp)	263123	31142
Minimum sequence length (bp)	10816	1004
Maximum sequence length (bp)	11790407	701709
N50 sequence index (# of contigs)	2	62
N50 sequence length (bp)	8783590	170721

The base quality of the assembly of the four core chromosomes was checked by mapping the HiSeq reads of isolate 2516 to the reference assembly. Over these 38.13 Mb, only one single nucleotide polymorphism (SNP) was detected between the HiSeq and SMRT reads. Two hundred and twenty-two variants were detected in homopolymeric stretches of nucleotides and low complexity (low GC%) regions (219 and 3 respectively). For these variants, read mapping was inconclusive for both HiSeq and SMRT reads, with both read batches giving support for different nucleotide calls.

**Fig. 1:**
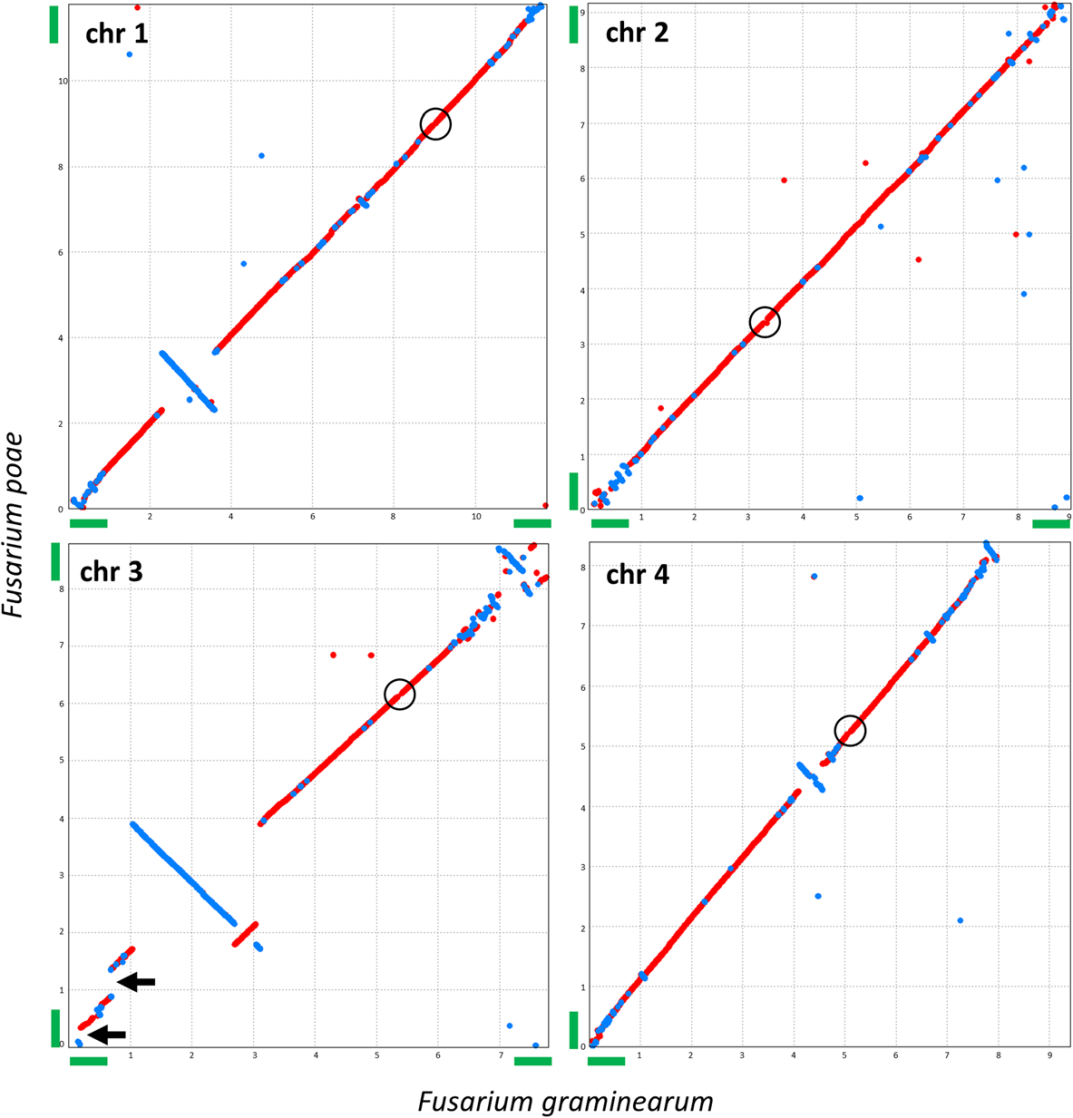
Chromosome alignments between *F. graminearum* (x axis) and *F. poae* (y axis). The best 1:1 alignment is shown between the four chromosomes of *F. graminearum* and the four core chromosomes of *F. poae*. Red indicates best hits in the same orientation while blue indicates inversions. The short arm of *F. graminearum* chromosome four ends in ~1.4 Mb of rDNA repeats that are not assembled in *F. poae*. All *F. graminearum* telomeres except the telomere of the short arm of chromosome 4 are assembled. For *F. poae*, the same telomere is lacking as well as the one on the long arm of chromosome 1. Telomeres that are assembled are shown with green bars on the arms of the chromosomes. Two insertions into *F. poae* chromosome 3 are denoted with black arrows. Approximate locations of the centromeres are shown with black circles (see Additional file 18)

Figure 1 shows the result of a whole genome alignment of the four core chromosomes of *F. poae* isolate 2516 and *F. graminearum* reference isolate PH-1. The latter was recently assembled to the chromosomal level [35]. Aside from two major chromosomal inversions (~2.2 Mb in chromosome 3, ~1.35 Mb in chromosome 1) and several smaller ones, the core chromosomes show extensive macrosynteny between the two species. Moreover, the entire *F. graminearum* sequence complement is present in our assembly, with the exception of 185 copies of the rDNA repeat at the end of chromosome 4. Two blocks of 204 kb and 464 kb on chromosome 3 of *F. poae* 2516 do not show synteny with *F. graminearum* (black arrows in Fig. 1) and are described in detail further below.

The remaining 172 contigs (8.18 Mb of the total 46.3 Mb) do not show any synteny with sister species *F. graminearum*. These 172 contigs contain eight copies of the ZIT1 transposable element (TE) described earlier as a specific marker for supernumerary chromosomes in *F. poae* [34]; this element was not found on the four core chromosomes. Not only ZIT1, but all TEs show an unequal distribution between the core chromosomes and supernumerary contigs, which is described in detail further below. The most striking difference in TE distribution comes from a Miniature Inverted–Repeat Transposable Element (MITE) that is the most abundant repetitive element in the genome. All 712 copies (with expect value < e^-10^) were found dispersed over the core chromosomes. In sharp contrast no MITE was found on the 172 supernumerary contigs. Its positional conservation among *F. poae* isolates was investigated, and for all four isolates the localization of the vast majority of MITE instances (97.7-99.8 %) was identical to at least one other isolate.

Taken together, the 172 contigs are likely to make up one or more supernumerary chromosomes, and they are designated as the “supernumerary genome” for the purpose of this study. The 8.18 Mb that the 172 contigs contain is likely a slight overestimation, as in some instances the end of one contig is collinear and identical to the start of the contig that follows in the assembly, potentially indicating a (partial) double assembly (see further below for example). Besides the four core chromosomes and 172 supernumerary contigs, the assembly contains the mitochondrial genome of 138 kb, and three mitochondrial plasmids. These plasmids were confirmed to be mitochondrial as their ORFs encode a reverse transcriptase only with the mold mitochondrial genetic code.

### A high quality machine annotation

Isolate 2516 was grown in six diverse conditions, to stimulate transcription of as many genes as possible. RNA was extracted and sequenced, and 659 076 900 RNAseq sequence reads were obtained. These were quality trimmed and the resulting 562 136 710 reads were used in the BRAKER1 pipeline [36]. This is a novel annotation method that uses RNAseq reads as extrinsic evidence, to annotate the genome in a rapid and automated way without any manual curation steps. In total 14817 genes were predicted for isolate 2516. Table 2 lists some core features of the machine annotation of *F. poae* 2516 compared to the annotation of *F. graminearum* PH-1 [35].

**Table 2:** General features of the machine annotation of *F. poae* 2516. These were compared to the features of the published annotation of *F. graminearum* PH-1 [35]

	*F. poae*			*F. graminearum*
	Total	Core	Supernumerary	
Genome size (bp)	46 309 701	38 129 297	8 180 404	37 958 956
GC%	46.30 %	46.00 %	47.60 %	48.20 %
# of genes	14 817	12 097	2 720	14 160
Mean gene density (per Mb)	320	317	332	373
Median gene length (bp)	1 391	1 406	1 309	1 257
Avg introns/gene	1.82	1.88	1.57	1.72
Median intron length (bp)	54	54	57	55

The BUSCO (Benchmarking Universal Single-Copy Orthologs) data set for fungi was used to assess whether the annotation can be considered complete [37]. This set comprises proteins that are very likely to be present in a queried genome, based on an analysis of other genomes within a particular kingdom. The predicted proteins from *F. poae* 2516 as well as the proteins from the most recent annotation of *F. graminearum* PH-1 [35] were analyzed by comparing them to the BUSCO data set. Table 3 shows the output for both species. The *F. poae* protein set is assessed at equally high quality as the *F. graminearum* set, indicating that the *F. poae* genome annotation is as accurate and complete as the *F. graminearum* annotation*.*

**Table 3:** BUSCO analyses of *F. poae* and *F. graminearum*

Organism	Complete	Fragmented	Missing	Total
*F. poae*	1431	7	0	1438
*F. graminearum*	1432	6	0	1438

Fragmented BUSCOs are proteins that are only partially recovered [37]. These were analyzed manually (Additional file 1). Three of the fragmented BUSCOs were shared between *F. poae* and *F. graminearum*, and examination of the RNAseq data did not provide conclusive evidence that the genes are miss-annotated. The remaining four and three proteins of *F. poae* and *F. graminearum* represent gene models that are likely to be miss-annotated in these species. In all four cases of *F. poae* and in one case of *F. graminearum*, a hybrid gene model was built from two separate genes. The remaining two *F. graminearum* gene models respectively lack two exons and contain two exons in excess. The validation of all fragmented BUSCOs with RNAseq data can be found in Additional file 1. For *F. graminearum* an RNAseq data set described before was used [38]. The BUSCO analysis suggests that the annotation of *F. poae* 2516 did not miss any conserved genes, and within the conserved genes, <0.5 % is miss-annotated.

### The ingredients for meiosis and RIP are present in the genome

RIP only functions during the sexual cycle, which has not been definitively shown in *F. poae*. Therefore the conservation of the necessary ingredients for meiosis was investigated for isolate 2516. The MAT1-1 locus was extracted from the assembly, and its architecture is presented in Additional file 2. As all four isolates in this study have the MAT1-1 mating type, the architecture of the MAT1-2 locus could not be investigated. The number, order and direction of the genes occupying the MAT1-1 locus is identical to that in other *Fusarium* species [39]. The *MAT1-1-1*, *MAT1-1-2* and *MAT1-1-3* genes have a predicted ORF with high similarity to those found in related species (85, 86 and 92 % similarity to proteins from *F. graminearum*). The *MAT1-1-1* gene was previously identified for *F. poae* [40] and has 99 % protein similarity with the gene model in this study. Transcription of *MAT1-1* idiomorph was noted in the RNAseq data, and the predicted splice forms lead to functional proteins (Additional file 2). A collection of 60 isolates was screened for the presence of MAT1-1 and MAT1-2 and both idiomorphs were detected, albeit in heavily skewed distribution (Additional file 3).

The KEGG pathway for meiosis in *F. graminearum* (fgr04113) was consulted to identify proteins involved in a putative sexual cycle. The conservation of this ‘meiotic toolbox’ was investigated in *F. poae*. All fifty-one entries in fgr01443 give best reciprocal protein hits with *F. poae* at expect values below 10^-150^, indicating that all ingredients of the meiotic toolbox are present in *F. poae* (Additional file 4).

Only a limited number of genes have been identified that belong to the machinery for RIP. A homolog of the *rid* (RIP defective) gene, shown to be vital for RIP, of *F. graminearum* is present, intact and transcribed in *F. poae* (Additional file 2). However, the expected intron was not spliced in the RNAseq data and the splice variant that was observed encodes a protein with a premature termination of translation.

### Distribution of transposable elements differs markedly between core and supernumerary genome

**Fig. 2:**
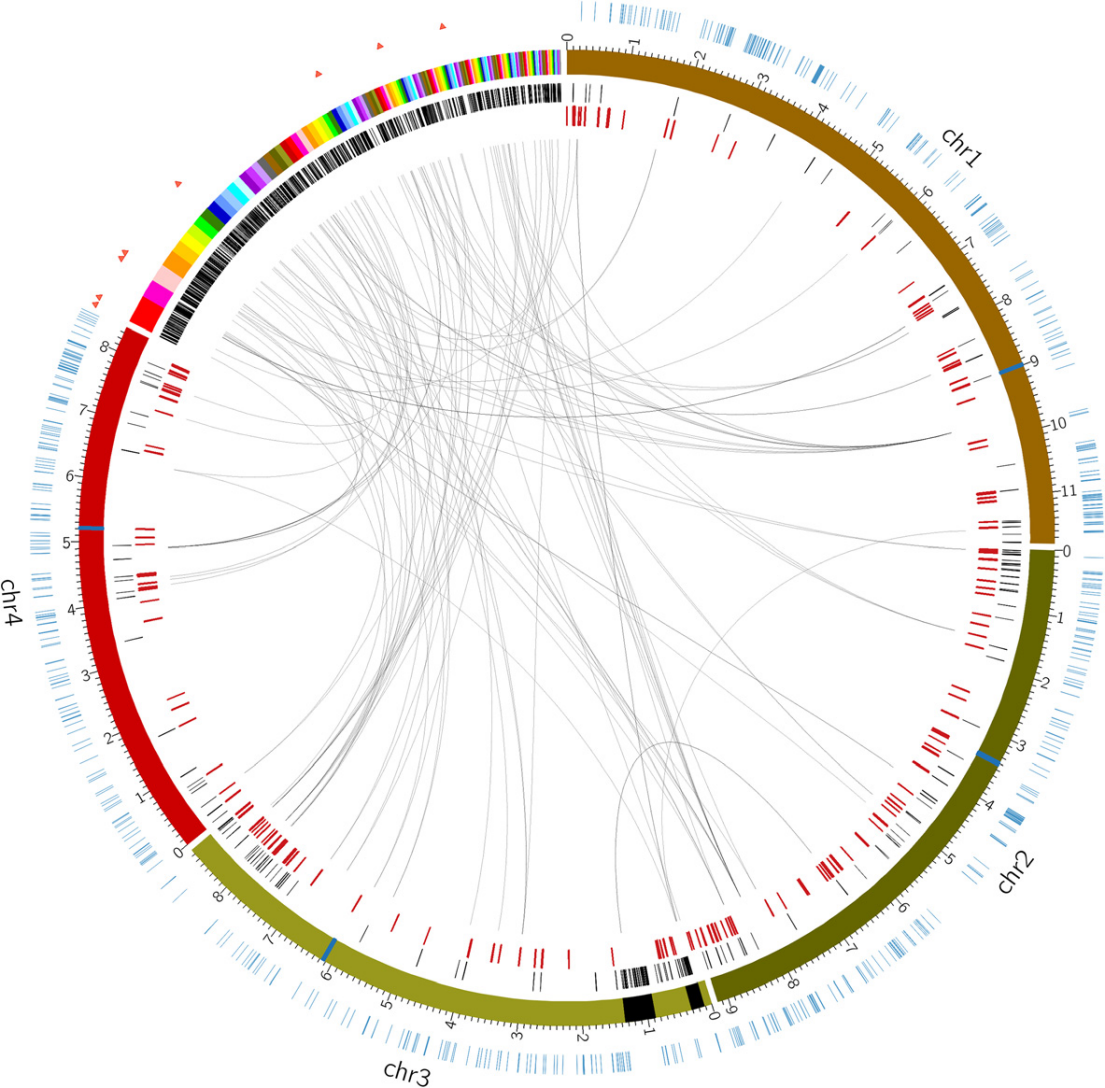
Circos plot showing differences between the core and supernumerary parts of the genome. Outer circle: blue lines denote the distribution of a MITE, red triangles denote ZIT1 copies. Second circle: core chromosomes and supernumerary contigs are colored, blue blocks on the chromosomes indicate the centromeres, black blocks show the two insertions of supernumerary sequence into the core chromosomes. Third circle: black lines represent intact (not RIPped) copies of TEs. Fourth circle: red lines represent RIPped copies of TEs. At the center of the plot, black lines connect gene duplications between the core genome and the supernumerary genome. Only protein hits larger than 266 amino acids are shown as their corresponding genes are supposed to be above the length threshold for RIP. Duplications within the supernumerary genome are not mapped

Table 4 lists the distribution of TEs throughout the genome. Figure 2 visualizes the chromosomal distribution of TEs. Many TE families only contain copies on the supernumerary part of the genome. RepeatMasker analysis with the identified TEs classified 2.1 % of the core genome and 25.6 % of the supernumerary genome as TEs.

**Table 4:** Classification and key characteristics of TE families in the genome of *F. poae* 2516. Elements below the length threshold for RIP are not included (MITE, ZIT1). Repetitive elements such as the rDNA tandem and two families of telomere linked RecQ helicases are not included. Nomenclature of TEs is as recommended in literature [73]. *R* retrotransposon, *D* DNA transposon, *L* long terminal repeat (LTR), *T* terminal inverted repeat (TIR), *G* Gypsy, *C* Copia, *F* Fot1/Pogo, *T* Tc1/mariner, *M* Mutator, *A* hAT, *x* unknown. n/a designates instances where a TIR/LTR could not be detected for a specific element

	Core		Supernumerary		Size (bp)	LTR/TIR (bp)	Family
	Intact	RIP	Intact	RIP			
Retrotransposons							
RLG_*Maggy*	27	25	11	-	5684	240	Gypsy/Ty3 like
RLG_*Skippy*	5	7	13	-	6561	379	Gypsy/Ty3 like
RLC_*Ghost*	-	1	14	-	4900	195	Copia/Ty1 like
Rxx_*marsu*	-	-	30	-	2234	n/a	unknown
DNA transposons							
DTF_*Fot4*	1	-	-	-	1852	48	Pogo
DTF_*Fot8*	-	-	1	-	2133	43	Pogo
DTF_*Fot2*	-	2	41	-	2220	90	Pogo
DTF_*Fot3-A*	-	1	7	-	2212	75	Pogo
DTF_*Fot3-B*	-	1	20	-	2200	73	Pogo
DTF_*Fot3-C*	-	-	9	-	2203	73	Pogo
DTF_*Fot5-A*	40	10	9	-	1865	51	Pogo
DTF_*Fot5-C*	-	15	7	-	1865	51	Pogo
DTF_*ESP4-A*	-	-	21	-	2909	98	Pogo
DTF_*ESP4-B*	12	11	24	-	2868	90	Pogo
DTF_*Drogon*	33	-	24	-	1934	51	Pogo
DTF_*Viserion*	8	6	8	-	2885	84	Pogo
DTF_*Rhaegal*	-	1	10	-	1854	36	Pogo
DTF_*Balerion*	-	-	12	-	2749	79	Pogo
DTA_*RLT1*	-	-	17	-	2912	27	hAT-like
DTA_*RLT2*	-	-	11	-	2975	22	hAT-like
DTA_*RLT3*	-	-	12	-	2954	n/a	hAT-like
DTA_*Hornet1*	-	1	10	-	2613	n/a	hAT-like
DTA_*Hornet2*	-	-	22	-	2739	n/a	hAT-like
DTA_*Hornet3*	-	-	11	-	2965	n/a	hAT-like
DTA_*Tfo1*	-	-	20	-	2852	28	hAT-like
DTA_*Tfo2*	-	-	15	-	2838	26	hAT-like
DTA_*Drifter*	-	-	23	-	2779	n/a	hAT-like
DTA_*Obara*	-	-	13	-	3867	19	hAT-like
DTA_*Nymeria*	-	-	36	-	2850	30	hAT-like
DTA_*Obella*	-	-	5	-	2480	29	hAT-like
DTA_*Sarella*	-	-	12	-	4236	n/a	hAT-like
DTM_*Hop7*	8	10	2	-	3449	81	Mutator
DTM_*Hop4*	1	-	7	-	2825	97	Mutator

Transcription and splicing of the predicted introns were noted for many intact elements. Single nucleotide polymorphisms (SNPs) in the RNAseq reads allowed for the specific copy/copies of TEs that were transcribed, to be identified. These are included as TE prototypes in Additional file 5 and annotated in Additional file 6. The functional and structural features of every TE were used for the classification into superfamilies (Table 4). Additional file 6 also contains phylogenetic trees of every element for which a protein coding sequence could be determined. In most cases, TE phylogeny lines up well with species phylogeny. Exceptions are the TEs DTA_*Nymeria* and DTM_*Hop7*, that show higher similarity to elements from unrelated fungi than to elements from related *Fusarium* species.

### Unbalanced RIP between core and supernumerary genome

RIPcal was used to analyze the occurrence of RIPped copies from the 33 transposable element families, separately for the core and supernumerary genome. The results are presented in Additional file 7. On the core genome, there are 13 families that show RIPcal patterns that are typical for RIP (dominance of CpA → TpA mutations; red trace). On the supernumerary genome, there are no such instances. The alignments that were used to perform RIPcal analysis were then manually examined in an attempt to quantify the number of RIPped copies per family, on the core and the supernumerary genome. Indeed, RIPped copies of TEs were only detected on the core genome (Table 4).

In a few cases, low complexity regions on the supernumerary genome resemble RIP of intact elements. These also contain most transversions when compared to genuinely RIPped copies (Additional file 8).

### Transposable elements copy number is dynamic between isolates of the same species

**Fig. 3:**
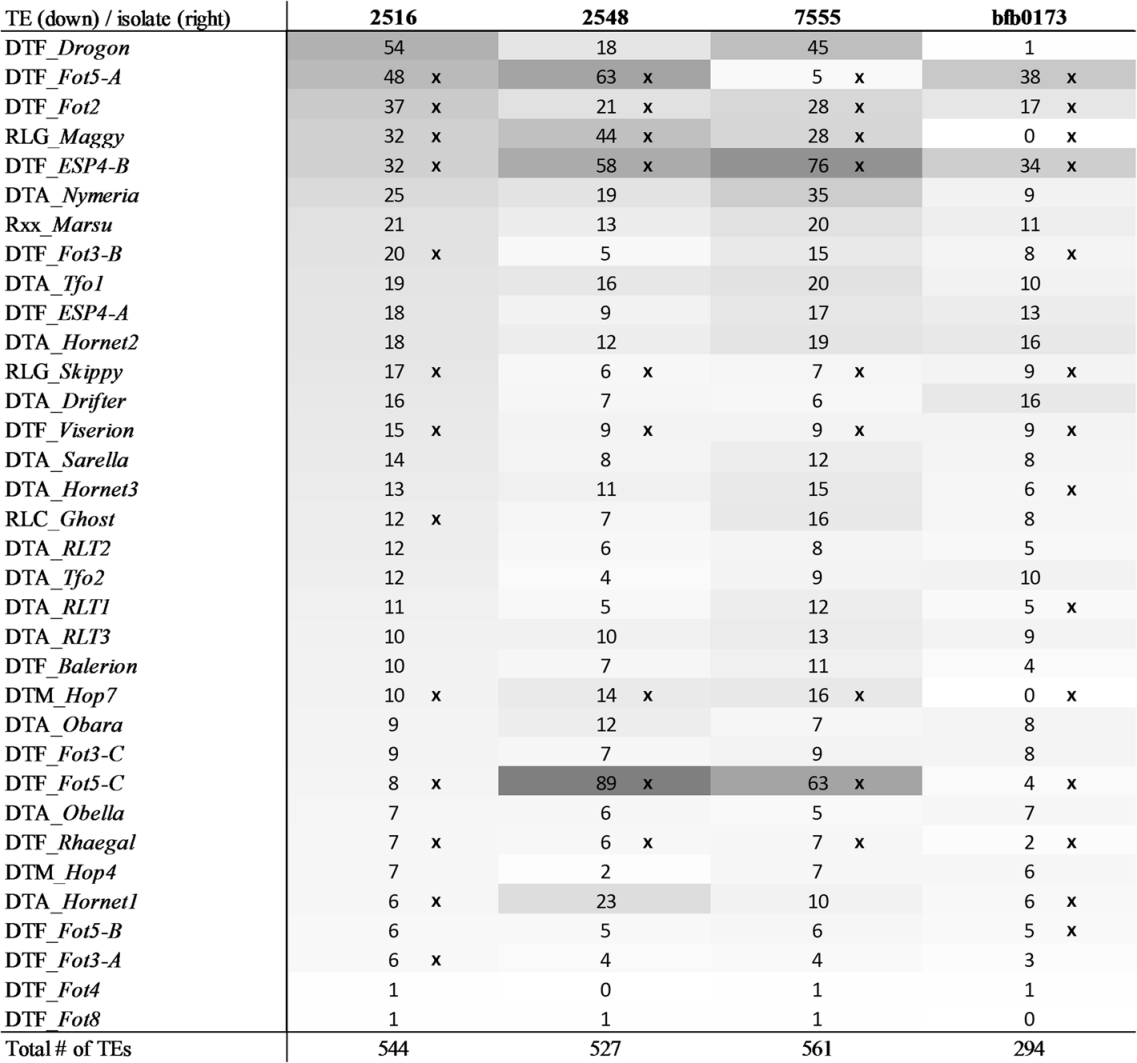
Estimation of TE numbers in the different *F. poae* isolates used in the study, as determined by a coverage-based method. Repeat families are classified in decreasing order of incidence in the genome of *F. poae* 2516; only class I and II transposable elements that are intact in *F. poae* 2516 are included, therefore elements such as the rDNA tandem and two families of telomere linked RecQ helicases are not in the table. X denotes families for which RIP was detected. It should be noted that average read coverage does not account for possible truncations and therefore the numbers in this table should be considered an estimate

Figure 3 shows the TE copy number variation between the isolates in this study. Based on genome coverage the most abundant element in isolate 2516 (DTM_*Drogon*) occurs only once in isolate bfb0173, a strain originating from China. As its ORF and TIRs are intact, it remains unknown why this element has not proliferated in isolate bfb0173. Isolates 2516 and 2548 were isolated from the same Belgian field at the same time, but show sharp differences in TE copy number. Two families that contain multiple copies in the Belgian isolates, are not present as intact copies in the Chinese isolate (RLG_*Maggy*, DTM_*Hop7*). However, RIPped copies present in the genome of bfb0173 indicate that during the evolution of the lineage that isolate bfb0173 belongs to, intact copies of these families have been present but were effectively eradicated from the genome.

Interestingly, RIP of certain elements seems isolate-specific, such as DTA_*RLT1* in isolate bfb0173 and RLC_*Ghost* in isolate 2516. A process similar to the loss of DTM_*Hop7* and RLG_*Maggy* in isolate bfb0173 may have occurred species-wide, as RIPped elements in isolate 2516 were detected of up to 14 families that no longer contain any intact copies in this isolate, or any other isolate in this study. For a retrotransposon of the Gypsy family, RIPped copies are present in all isolates, but only isolate 2548 contains intact copies.

### Localization and divergence of transposable elements differs between the core and supernumerary genome

The localization and divergence of the intact TEs was investigated. One hundred and thirty-five intact TEs are present on the core chromosomes of isolate 2516. Figure 3 shows that elements of these families are often, but not always, also present in multiple copies in the genomes of the other isolates in this study. However, read mapping shows that none of the 135 elements on the core chromosomes of isolate 2516 are present in the same location in isolates 2548, 7555 and bfb0173 (as exemplified in Additional file 9). The integration of these elements therefore seems to have happened recently.

**Fig. 4:**
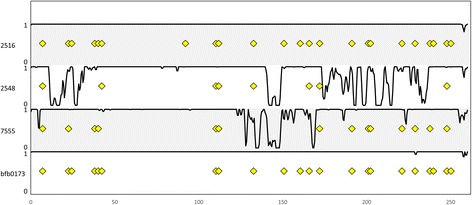
Integration of intact TEs on supernumerary contig 308. The graphs shows in a sliding 1 kb window the fraction of bases from the reference contig that is covered by HiSeq reads of every isolate (value between 0 and 1). The upper track shows all TEs on contig 308 of isolate 2516 that are >1 kb and >90 % identity to the element prototype (Additional file 5) with yellow dots. This TE landscape was used for comparison with isolates 2548, 7555 and bfb0173. Dots for these three isolates indicate elements for which there is read mapping that an element has integrated in the exact same location as the element in isolate 2516 (and is therefore ancestral). Dots that align vertically are conserved in multiple isolates

In contrast, on the supernumerary genome of isolate 2516 elements can be found that show identical integration in isolate 2516 and one or more of the other isolates. Figure 4 illustrates this for supernumerary contig 308. The four tracks show the TE presence (dots) and genome coverage (lines) for every isolate. Several elements have identical flanks in all isolates, indicating that they are ancestral integrations (dots that line up vertically in Fig. 4). Additional file 10 shows the profile for supernumerary contigs 668, 561 and 550. Together these four contigs are the largest supernumerary contigs, totaling 1.26 Mb. Whole blocks of sequence are absent from some isolates: most of contig 550 in isolate bfb0173, parts of contig 561 in isolates 2548 and bfb0173. This absence/presence of sequence on the supernumerary genome is not cumulative for any one isolate or contig investigated. Moreover, the integration of TEs on the supernumerary genome is also not concordant with vertical inheritance. This is illustrated in Additional file 11. The recombination-like picture of sequence absence/presence and TE integration, may reflect the dynamics the supernumerary chromosomes undergo during crossing.

**Fig. 5:**
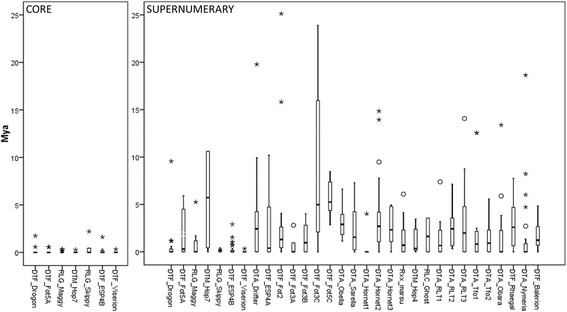
Divergence estimation of intact (not RIPped) TE copies on the core (left) and supernumerary (right) genomes. Copies were aligned and branch lengths extracted from a maximum-likelihood phylogenetic tree. Branch lengths were used to calculate divergence times with a fixed substitution rate (1.05 * 10^-9^ substitutions per site per year [66]). Y axis scale was cut off at 25 Mya, but for the supernumerary genome many outliers are above this value. Additional file 19 shows the boxplot with outliers for the supernumerary genome. The boxes for every TE show the lower and upper quartile of the divergence estimates and the median (thick line within the boxes). The whiskers represent the minimum and maximum values. Circles and asterisks are outliers and extreme values which fall respectively outside of one-and-a-half additional box lengths and three additional box lengths counted from the upper quartile limit

A comparison was made between the estimated divergence time of TEs in the core genome and those in the supernumerary genome of isolate 2516. This is based on the principle that when TEs are present at a certain location for a longer period of time, they gradually accumulate more SNPs, which can be used to calculate the time elapsed since their insertion. As can be seen in Fig. 5, TEs in the supernumerary genome are more divergent and are therefore presumed to result from more ancient transposition events.

### The core genome is invaded by transposable elements from the supernumerary genome

On the supernumerary genome, TEs are more divergent than TEs on the core chromosomes and some are present at identical sites in at least two isolates. We therefore wanted to test whether TEs in the core genome may originate from the supernumerary genome. This directionality was best illustrated by one element of RLG_*Skippy* located on the short arm of chromosome 3. This TE has recently integrated into the genome of isolate 2516 and contains 23 SNPs compared to the other copies on the core chromosomes of this isolate. These 23 SNPs, together with one additional SNP, are also present in a copy on the supernumerary genome, that is at the exact same location for all isolates and therefore is an ancestral insertion.

The recent integration of TEs into the core genome of isolate 2516 has sometimes occurred within the coding region of genes. The environment of all 135 recent integrations in the core genome was investigated (Additional file 12). Ten instances were found where integration disrupted a gene. Notably, DTF_*Drogon* integrations account for nine of these. Remarkable differences can be detected between the environments of the different TE families. DTF_*Fot5-A* elements consistently integrate within RIPped or low complexity (low GC%) environments, while DTF_*Drogon* elements have integrated within regions of average GC%.

An extreme case of core genome invasion is found near the telomere of the long arm of chromosome 3. Two sequence blocks do not show any synteny with *F. graminearum*, with coordinates 115073-319336 (204 kb) and 883738-1348064 (464 kb). Analysis of the flanking sequences of these two regions shows that they are continuous in isolates 2548, 7555 and bfb0173. Therefore, these regions represent translocations of supernumerary sequence to the core genome of isolate 2516. All parameters that were used to compare the core and supernumerary genome in this study, support the classification of these sequence blocks as supernumerary sequence. For the purpose of this study they have been regarded as part of the “supernumerary genome”. The 204 kb insertion is an underestimate as it holds a 5000 N placeholder, where presumably one or more of the 172 supernumerary contigs belong.

These insertions into the core genome, both of single TEs and whole blocks of supernumerary sequence, may have large implications for the biology of the organism such as respectively gene disruptions and hampered meiotic alignment. We investigated how common the two supernumerary sequence translocations are in a population of 60 *F. poae* isolates and found that seven isolates contain the first insertion, closest to the telomere. These seven isolates were isolated from three different locations in Belgium. Three of these seven isolates also contain the second insertion, at 883738 bp into chromosome 3 (Additional file 3). Isolate 2516 is one of these three isolates. Isolates 2548, 7555 and bfb0173 were confirmed not to have any of the insertions.

### The supernumerary genome is a refuge for gene duplications

The absence of paralogs is a hallmark of a RIP-active species [20]. In a blastp of all proteins encoded by genes on the core chromosomes against themselves, no hits with identity >80 % and length above the RIP length threshold can be found. This confirms that the core chromosomes are subjected to RIP. Additional file 13 shows the blastp output of all proteins encoded on the supernumerary genome queried against those encoded in the core genome. Many hits well above the RIP length threshold show high protein identity (>80 %) and represent genuine gene duplications that have not been inactivated by RIP. A total of 44 genes on the core chromosomes have one or more duplicates in the supernumerary genome, totaling 104 hits on the latter. Figure 2 visualizes these gene duplications as lines connecting both the gene on one of the four core chromosomes and its paralog(s) on the supernumerary genome.

Additional file 13 lists the functional annotation of these duplicated genes. Notable instances include the transcription factor *EBR1* [41, 42], key component of the RNA silencing pathway *Dicer2* [43] and the secondary metabolite backbone gene *PKS8* [44]. In the case of *EBR1*, up to 11 duplications were found. To ascertain that these are not artefacts from the assembly, the duplicated sequences and their 500 bp flanking regions were aligned. Assembly artefacts are identified by sequence alignments with nearly 100 % sequence identity across the entire region. Five of the 104 duplications were identified as likely double assemblies. The remaining 99 instances are likely genuine gene duplications (Additional file 13). Additional file 14 shows an example of a potential assembly artefact as well as an example of an expected duplicate gene.

The presence of the duplicated genes was assessed between the different isolates of *F. poae* in this study. Additional file 13 shows that some duplications are unique for isolate 2516, while others are not cumulative over the different isolates. Indeed, isolates 2548, 7555 and bfb0173 each share unique duplications with isolate 2516 that are absent in the other isolates.

## Discussion

Chromosomes that vary in both size and number, and have an uneven distribution among individuals of the same species, have been described in animals [45], plants [46] and fungi [8]. Throughout these kingdoms they are identified as supernumerary, accessory, dispensable or B chromosomes, in contrast to the core or A chromosomes. These supernumerary chromosomes show distinct features compared to the core chromosomes: they can be high in repeats and transposable elements [10], have different gene density and function [47] and/or GC-content [12], are epigenetically very dissimilar [48], can be transmitted by horizontal transfer [11] and are unstable in meiosis [29]. This sharp contrast between the two sets of chromosomes can be explained by different evolutionary pressure, different origins, or a combination of both [6, 13]. The functions of the supernumerary chromosomes are unclear. Speculations range from selfish DNA fragments without benefit to the host [49] to components that are critical for pathogenicity and survival [11]. As they occur in the same nucleus, this raises questions how the different sub-genomes are managed within the organism and if there are potential conflicts and interactions. The dynamics between the core genome of an organism and its extra-chromosomal DNA have been studied for plasmids in bacteria [50], and mitochondrial DNA insertions into the nuclear genome of many eukaryotes [51]. In this study we aimed to provide a contiguous genome assembly of a fungal pathogen that contains supernumerary chromosomes, and to compare it with a sister species that contains none. This allowed us to determine differences in genome biology as the causal agent for the differences between the core and supernumerary genomes, and to observe a significant crosstalk between them as a result.

The assembly using SMRT long reads allowed the division of the genome into a core and supernumerary part, a feature that was not possible with the assembly using short reads due to the occurrence of highly repetitive DNA. The advantages of SMRT sequencing for fungal genome assembly was recently demonstrated for *Verticillium dahliae* [52]. The core chromosomes of *F. poae* showed a high level of macrosynteny with *F. graminearum* and cover the entire *F. graminearum* sequence complement. They showed characteristics of chromosomes under the control of RIP, such as many inactivated TEs and no gene paralogs with high sequence identity. The opposite holds for the supernumerary chromosomes, and the absence of RIP on the supernumerary genome is responsible for the differences between the two core and supernumerary genome in *F. poae*. Indeed, on the supernumerary chromosomes, no RIPped TE copies are found, and many duplicated genes are present. This is the first time a definitive causal agent is identified for the sharp contrast between the core and supernumerary chromosomes in a fungal pathogen.

The different rules that govern the core and supernumerary chromosomes, lead to a genetic crosstalk between them. We found many cases of exchange of genetic material between the core and supernumerary genomes. Genes from the core chromosomes are duplicated to the supernumerary genome, where some of these genes have undergone further copy number expansion. Vice versa, transposable elements originating from the supernumerary genome, have integrated into the core chromosomes and, in some instances, have led to gene disruptions. Most drastically, large sequence blocks (>200 kb) have been translocated from the supernumerary genome to the core chromosomes. Translocation of whole regions from supernumerary chromosomes to core chromosomes is not restricted to *F. poae*. A region of core chromosome 1 of *F. oxysporum* f. sp. *lycopersici* has all the characteristics of supernumerary sequence [8, 11]. It has been shown that this region is highly syntenic with one of its supernumerary chromosomes [14]. This situation may have arisen from ancient translocation followed by chromosome gain, or by duplication and integration of the supernumerary sequence. Our results show that unique events such as large insertions into the core chromosomes give rise to novel genotypes in *F. poae*, which may be able to rapidly spread as they were recovered from three different locations. Interestingly, both in *F. poae* and *F. oxysporum* f. sp. *lycopersici*, insertion of blocks of supernumerary sequence has occurred close to a telomere of core chromosomes, which supports the finding that core chromosomes in *Fusarium* species may be divided into distinct chromosomal regions on both a structural and functional level [14, 53].

The evolutionary advantage of a genome region not burdened by RIP defense is evident from the many gene duplications occurring specifically on that region. The primary versus secondary metabolism master regulator *EBR1* is present as a single copy gene in *F. graminearum* [42]. In *F. oxysporum* f. sp. *lycopersici* it has undergone gene duplication on the supernumerary chromosomes [41], which seemingly has independently occurred on the supernumerary chromosomes of *F. poae*. In *Metarhizium*, it has been hypothesized that an arrest of RIP was instrumental in the evolution to generalist infection agents [54], and the advantages of a temporary RIP relaxation or arrest may include accelerated evolution and divergence between related species [2]. The mechanism that gave rise to the existence of supernumerary chromosomes in *F. poae* is unknown. In *Z. tritici*, it has been suggested that supernumerary chromosomes may have originated from core chromosomes and subsequently degenerated and evolved separately [30]. A recent large scale duplication seems unlikely for *F. poae*, as an ancient MITE that characterizes the core chromosomes is completely absent from the supernumerary chromosomes. The contribution of horizontal chromosome transfer remains to be investigated, but has likely contributed to the supernumerary chromosome diversity in other species [11, 55].

Specific exclusion of duplicated genes from RIP has been observed before in *F. solani* (formerly known as *N. haematococca*), a species distantly related to *F. poae* [10]. Similar to those in *F. poae*, its supernumerary chromosomes are rich in TEs and gene duplications. It was experimentally determined that progeny, of which one of the parents contained two copies of the *hph* marker gene, contained both an intact and a RIPped copy of that gene [10], contrary to the standard *modus operandi* of RIP wherein all copies are inactivated. Apparently, a region in the genome of *N. haematococca* is excluded from RIP, but it is not known whether this coincides with the supernumerary chromosome(s). The regional variability of RIP extends beyond supernumerary chromosomes however. Nucleolus organizer regions (NORs) contain the rDNA tandem repeats in many fungal species, and within this region they are protected from RIP [22]. Either this is an active form of protection, which may be similar to what is happening on the supernumerary chromosomes of *F. poae*, or rDNA is mutated by RIP and mutated copies subsequently undergo reduced intrachromosomal recombination to give rise to a full-length rDNA tandem during meiosis [23].

It is thought that RIP functions after plasmogamy but before the final pre-meiotic DNA replication and karyogamy. It works multiple times during the rounds of nuclear division that occur at this point, presumably during G1 or near the replication fork during the S phase [23]. It functions only in the nucleus, or nuclei, that contain(s) DNA duplications and does so on a single DNA strand. Mis-pairing of duplicated DNA has been hypothesized to deliver the substrate for RIP [23]. Why supernumerary chromosomes in *F. poae* escape RIP is unknown. While physical alignment of duplicated copies is presumed to be important, the exact search mechanism for homology is unknown. Clutterbuck [26] proposed two hypotheses for the function of RIP that implicate the temporal or spatial proximity of haploid nuclei in dikaryotic cells, where RIP acts, to diploid cells undergoing meiotic pairing. This pairing was previously shown to be hampered for supernumerary chromosomes through their high variability [29]. The splice form of the *rid* gene, shown to be vital for RIP, that was detected in this study does not lead to a functional protein. One explanation for this may be that the gene model constitutes a case of crucial alternative splicing, and the *rid* gene is only correctly spliced in the pre-meiotic phase.

Supernumerary or extra-chromosomal structures are considered to be evolutionary cradles for pathogenicity in viruses, bacteria and fungi [6]. We showed that they shelter TEs and gene duplications in a fungal plant pathogen, and that large sequence blocks may translocate to the core genome, with a profound effect on genome biology. The “living apart together” dynamic between the core and supernumerary genome is explained by different rules that govern both genome compartments. Future investigation will be able to further characterize the core versus supernumerary chromosomes in parameters that have been shown to be relevant in other pathogens such as histone modification [48], transcription [14] and gene content [11].

## Conclusions

The genome biology of the related plant pathogenic fungi *F. poae* and *F. graminearum* primarily differs by the presence of supernumerary chromosomes in *F. poae*, not present in *F. graminearum*. These supernumerary chromosomes differ markedly from the core chromosomes. We provided a high quality genome assembly for *F. poae* and determined the cause of these differences to be RIP, a mutational defense mechanism against TEs which functions on the core chromosomes but not the supernumerary chromosomes. This has led to a dynamic crosstalk between the core and supernumerary genome, which significantly affects chromosomal and organismal evolution.

## Methods

### Fungal material

Table 5 lists the *F. poae* isolates that were used for whole genome sequencing. An additional sixty *F. poae* isolates were collected from various sources (Additional file 3) and used for diagnostic PCRs (see further).

**Table 5:** Isolates used for whole genome sequencing

ID	Location	Year	Host	Reference
bfb0173	China	2005	barley	[74]
2516	Belgium	2011	wheat	this study
2548	Belgium	2011	wheat	this study
7555	Belgium	1965	wheat	MUCL

*MUCL* Mycothèque de l’Université catholique de Louvain (Louvain-la-Neuve, Belgium)

### Nucleic acid manipulation, library preparation and sequencing

Detailed information on growth conditions and nucleic acid manipulation can be found in Additional file 15. In short, for HiSeq sequencing, DNA was extracted from isolates bfb0173, 2516, 2548 and 7555. DNA from isolate bfb0173 was used for random sheared shotgun library preparation using the NEXTflex ChIP-seq Library prep kit with adaptations for low DNA input according to the manufacturer’s instructions (Bioscientific). The library was loaded as (part of) one lane of an Illumina Paired End flowcell for cluster generation using a cBot. Sequencing was performed on an Illumina HiSeq2000 instrument using 101, 7 and 101 flow cycles for forward, index and reverse reads respectively. De-multiplexing of resulting data was carried out using Casava 1.8. Shotgun libraries were made for isolates 2516, 2548 and 7555 using the Illumina TruSeq LT DNA sample prep kit according to the manufacturer’s instructions (Illumina). Libraries were then pooled equimolarly and loaded on one flowcell lane for 2x100 nt paired end sequencing on an Illumina HiSeq2000 platform as described above.

For SMRT sequencing, DNA was isolated from isolate 2516. Genomic DNA was extracted with the Wizard Magnetic DNA Purification System for Food (Promega) according to the manufacturer’s instructions. Twenty μg DNA was used for a large insert (10 kb) library prep according to the manufacturer’s instructions (Pacific Biosciences) with small adaptations (Additional file 16). After library prep and SMRT bell adapter ligation, the SMRT bells were size selected with a 7000 bp minimum cutoff. Sequencing was done on a Pacbio RS II system using one cell per well, C4 chemistry and 240 min movie time. A total of 16 SMRT cells was run.

RNA samples were prepared from isolate 2516 grown under different conditions, designed to have maximal numbers of genes expressed. Details can be found in Additional file 16. In short, samples were designed to favor different metabolic stages. Primary metabolism was simulated in a rich medium. Secondary metabolism was induced in five distinct conditions, namely trichothecene biosynthesis induction, fungicide application, N-starvation, C-starvation, and conidiation under UV. RNA was extracted from the rich medium and the 5 “stress-inducing” treatments with TRIzol (Life Technologies). Subsequently, the crude RNA was purified with the RNA cleanup protocol included in the RNeasy Plant Mini kit (Qiagen) according to the manufacturer’s instructions. RNAseq library preparation was performed using the Illumina TruSeq total RNA sample preparation kit and guidelines. Libraries were pooled equimolarly and loaded on one Illumina HiSeq2000 flowcell lane for 2x100 nt paired end sequencing as described above.

### Genome assembly

From SMRT Portal version 2.3.0.140893, the HGAP2 (Hierarchical Genome Assembly Process) was initiated using data from 16 SMRT cells. Raw reads were filtered on *Q* = 0.83, polymerase read length >1000 bp and subread length >1200 bp. The seed read length for the error correction procedure was manually set to 6 kb. After the error correction step, the data was filtered for reads >9 kb. With this dataset the assembly was performed with Celera, using the default settings provided by HGAP2. The contigs from this assembly (assembly A) were taken as the basis for a merged assembly, for which the contigs were supplemented with those derived from two additional automatic HGAP2 assemblies (assemblies B and C). One assembly was performed with 10 SMRT cells using default settings, and one with 16 SMRT cells using only HQ input data (read quality > = 0.85, polymerase and subread length >4000 bp).

The largest contigs, corresponding to a major part of a hypothetical chromosome were used as queries for blastn searches against the other contigs in the assemblies. Contigs were joined on the basis of their colinearity (usually excluding the very end of one contig and the very beginning of another, where the assembler presumably stalled or followed a wrong seed for a particular assembly) and their macrosynteny with other *Fusarium* species. Rightful joining of contigs was evaluated by mapping SMRT long reads. The resulting merged assembly of four chromosomes was polished using Quiver (SMRT Portal resequencing protocol) for 2 times, using HQ reads (read quality > = 0.80, polymerase and subread length >3000 bp). The merged assembly was supplemented with the remaining contigs and the degenerate unitigs from assembly A. Nine contigs that only contained rDNA tandem repeats, and 66 contigs that contained mitochondrial sequence, were removed. The remaining contigs were added to the four-chromosome assembly, and the entire assembly was error corrected in 1 pass using quiver (read quality > = 0.84, polymerase and subread length >1000 bp). After this quiver run, 13 contigs were removed from the assembly that had overall base quality scores close to zero, compared to an average base quality of 50 for the rest of the assembly.

The mitochondrial genome was assembled with GRABb using standard settings and with the PH-1 mitochondrion (NCBI accession HG970331.1) as bait (Brankovics et al., submitted). One mitochondrial plasmid was assembled in SMRT assembly A. Two additional mitochondrial plasmids were taken from the HiSeq assembly (see below), that were not present in the SMRT assembly. This may result from the fragment size selection that was performed, as the plasmids are <3 kb in size. The final assembly therefore contains four chromosomes, 172 unplaced contigs, one mitochondrial genome and three mitochondrial plasmids. For the four chromosomes, the error rate of the SMRT assembly was checked by mapping the HiSeq reads of isolate 2516 to the SMRT assembly with CLC Genomics Workbench 7.5 (length and similarity fraction = 0.8). For the four core chromosomes, basic variant detection was run with minimum coverage = 50, minimum count = 10 and minimum frequency = 70 %, and other settings at standard value. A *de novo* assembly of Illumina HiSeq reads for isolate 2516 was perfomed with CLC Genomics Workbench 7.5 using standard settings.

### Annotation of the reference genome

The Illumina HiSeq RNAseq paired-end reads were cleaned and trimmed using Trimmomatic [56]. Tophat2 [57] was used to map the trimmed reads to the SMRT assembly of isolate 2516. The mapping results were used in the genome annotation pipeline BRAKER1 [36] for training GeneMark [58] and Augustus [59]. BRAKER1 uses the introns parsed from the TopHat2 mapping as extrinsic evidence for the final gene models predicted by Augustus. The annotation is outputted as a GFF file with genes, introns, exons and the protein sequences predicted to be encoded by these genes. A short Python script was used to extract the protein sequences from the GFF.

### Repeat identification, localization, structural and functional characterization

RepeatModeler [60] was run on the genome of isolate 2516 with standard settings. RepeatModeler output was manually curated to obtain complete elements. These elements were then subjected to functional and structural characterization. When possible, terminal inverted repeats (TIR) and long terminal repeats (LTR) were identified. Bowtie2/TopHat2 read mapping as well as related NCBI accessions were consulted to find intron/exon boundaries. The translations of the predicted ORF for every TE were used as blastp queries against the NCBI non-redundant protein sequences database. The 15 best hits were aligned with the TE query using ClustalO [61] implemented in CLC Genomics Workbench 7.5. The resulting neighbor-joining phylogenetic trees are included in the TE data sheets. The elements were divided into superfamilies based on their domain similarities to described TEs. For RIP analysis, a copy coding for a functional protein (determined as described above) was used as a query for blastn in CLC Genomics Workbench 7.5 against the core and supernumerary genomes separately, at expect value < 1e^-10^. All hits were aligned to the query with ClustalO [61], and the alignments were fed directly to RIPcal for alignment-based RIP analysis with the query as the defined reference [62].

### Analysis of transposable element integration sites

Blastn was used to obtain genomic coordinates of all intact and RIPped copies of TEs. Using the getfasta utility of BEDtools [63] these hits, including their flanking regions, were extracted. HiSeq reads from all isolates in this study were subsequently mapped to the extracted reference sequences at high stringency (minimum length fraction 0.95, minimum similarity fraction 0.95) in CLC Genomics Workbench 7.5. Results were manually inspected to find identical genomic environments. Elements with read support for only one flank were also considered to be identically inserted.

For synteny of the MITE, a prototype of the element (see Additional file 5) was used as a blastn query against the entire genome (expect value < 1e^-10^), hits were extracted including 500 bp upstream and downstream flanking sequence with the getfasta utility of BEDtools [63]. This was done for the assembly with long reads of isolate 2516 and the short read *de novo* assemblies of isolates 2548, 7555 and bfb0173. For every isolate, the resulting sequence list was queried against the genome assemblies of the other isolates with blastn, and the matches longer than 640 bp were counted (indicating instances where the localization of the MITE coincides between isolates: at least one flank of 500 bp and the 140 bp element are shared).

### Divergence estimates of TE copies

Intact (not RIPped) copies were extracted as described above from the core and unplaced sequence separately. Only families containing five or more copies were retained. ClustalO alignments [61] were fed to PhyML [64] and maximum-likelihood phylogenies were built for every family with settings retrieved from literature [15]. Specifically, a neighborhood joining tree was used as starting tree, the transition/transversion ratio was 4, the HKY85 evolution model was used and distribution parameters were allowed to optimize. In the resulting phylogenies, terminal branch lengths represent the relative age of every separate element. These branch lengths were extracted from the Newick files with Newick Utilities [65]. Using the substitution rate determined for protein-coding genes in fungi (1.05 * 10^-9^ [66]), divergence time estimates were calculated from the branch lengths. These were then visualized as boxplots using SPSS.

### Paralogs and gene duplications

Blastp was used to find paralogs for all proteins (initial blastp parameters at expect value < 1e^-5^). Results were filtered to > 80 % identity and a length above the RIP threshold (+/- 800 nt or 266 amino acids). When the proteins encoded by genes on the core genome were queried against themselves, no hits other than self-hits were found. When the proteins encoded by genes in the supernumerary genome were queried against those from the core genome, 104 hits were obtained. These were formatted for Circos visualization. Using the gff2sequence tool [67] the 104 genes in the supernumerary genome and their 44 paralogs on the core chromosomes were extracted, including 500 bp up- and downstream. These sequences were aligned all-vs-all with Smith-Waterman using a python application based on PaSWAS [68], which produced local alignments in SAM [69] format.

### Genome visualization

Circos [70] was used for circular genome visualization. Locations of TEs were extracted from blastn output (expect value < 1e^-10^). Gene duplications above the RIP threshold were parsed from blastp output.

### Diagnostic PCRs

To determine the MAT1-1 and MAT1-2 distribution in the *F. poae* population, primer pairs POA-1-F/POA-1-R and POA-2-F/POA-2-R were used [40]. For the two insertions of supernumerary sequence into the core chromosomes, primers were designed flanking insertion site as well as covering the extremes of the inserted block (visualized in Additional file 16). All primers used in this study can be found in Additional file 17. PCR reactions were performed as described earlier [71]. Gel electrophoresis of the diagnostic PCRs is shown in Additional file 17.

### Comparative genomics intra- and inter-species

To estimate the number of intact copies for every family in the isolates that were sequenced with only Illumina technology, reads were mapped to the curated library of repeats (see above), and the resulting coverage was normalized against the mean coverage of the single-copy genome for each isolate. To estimate the coverage across the four largest supernumerary contigs, HiSeq reads from every isolate were mapped with CLC Genomics Workbench 7.5 (length and similarity fraction = 0.8) to the reference assembly which was masked for TE with RepeatMasker. BAM files were processed with the coverage utility of BEDtools [63] to find the fraction of bases covered by reads in a 1 kb sliding window. For whole-genome alignment, the genome of the reference isolate was masked using the curated repeat library with RepeatMasker. The masked genome was aligned with the completed genome of *F. graminearum* PH-1 using MUMmer [72].

## Abbreviations

BUSCO, benchmarking universal single-copy orthologs; LTR, long terminal repeat; MITE, miniature inverted-repeat transposable element; Rid, RIP defective; RIP, repeat-induced point mutation; SMRT, single-molecule real-time; SNP, single nucleotide polymorphism; TE, transposable element; TIR, terminal inverted repeat

## Additional files

Additional file 1: Fragmented BUSCOs in *F. poae* and *F. graminearum*. A: *Fusarium poae* predicted proteins that were identified as fragmented by the BUSCO analysis. The top track corresponds to the predicted gene model, the second track shows the predicted coding features and the bottom track shows the TopHat mapping of the RNAseq reads. Arrows indicate likely sites of miss-annotation (likely fusion of two separate genes). From top to bottom: g7865, g6381, g8721 and g6717. B: Predicted proteins in the *F. graminearum* set that were identified as fragmented by the BUSCO analysis. The top track corresponds to the predicted gene model, the second track shows the predicted coding features and the bottom track shows the TopHat mapping of the RNAseq reads. Arrows indicate likely sites of miss-annotation From top to bottom: FGRRES_16573 (likely fusion of two neighboring genes), FGRRES_10897 (likely addition of two exons), FGRRES_06268 (likely two exons missed). RNAseq data described in Zhao et al. [38] were used. C: Predicted proteins in the *F. poae* and *F. graminearum* set that were identified as fragmented by the BUSCO analysis and are shared between the two species. The top track corresponds to the predicted gene model, the second track shows the predicted coding features and the bottom track shows the TopHat mapping of the RNAseq reads. From top to bottom: g1567/FGRRES_05972, g1914/FGRRES_06308, g8796/FGRRES_09970. The first of the two visualizations corresponds to *F. poae*, the second corresponds to *F. graminearum*. (DOCX 754 kb) Additional file 2: Structural annotation and transcription of MAT1-1 and rid. A. Architecture of the MAT1 locus in *F. poae* isolate 2516, located at 3 120 000 bp into chromosome 2. The top track represents the predicted gene model, the second track represents the predicted coding features and the bottom track shows the TopHat mapping of the RNAseq reads. Note the correct splicing of introns for all three alleles. B. The *rid* (RIP defective) gene in *F. poae* isolate 2516, located at 2 232 000 bp into chromosome 2. The top track represents the predicted gene models, the second track represents the predicted coding features and the bottom track shows the TopHat mapping of the RNAseq reads. Two separate genes were predicted by the BRAKER1 pipeline. The *F. pseudograminearum* like gene model is superimposed as the single long coding feature. There is no splicing that supports this model under the conditions tested in this study. (DOCX 109 kb) Additional file 3: List of 60 *F. poae* isolates used for diagnostic PCRs. The origin, host and year of isolation are given. Mating type and insertion results are provided, methodology of the diagnostic PCRs are described elsewhere. (XLSX 12 kb) Additional file 4: Conservation of the KEGG pathway for meiosis. The table lists genes belonging to the KEGG pathway for meiosis in *F. graminearum* (fgr04113) and their counterparts in *F. poae*, as well as the predicted function of the *F. graminearum* gene. (XLSX 11 kb) Additional file 5: Transposable element prototypes. (FA 98 kb) Additional file 6: Transposable element datasheets. Information is listed for every transposable element family found in the genome of isolate 2516 that contains intact (not RIPped) elements. A: Tracks from top to bottom: LTR/TIR, coding region and mapping of RNAseq reads. B: Predicted protein used as bait for blastp, alignment of 15 best hits with the bait and neighbour-joining phylogenetic tree. For the retrotransposons, the pol protein was used. An asterisk denotes the *Fusarium poae* element in the tree. (PPTX 3451 kb) Additional file 7: RIPcal analysis of transposable element families on the core and the supernumerary genome. A comprehensive RIPcal analysis was performed on the core and the supernumerary genome separately. Based on an unbiased approach, 13 families out of 33 show patterns typical for RIP, and this is only detected on the core genome. (PPTX 735 kb) Additional file 8: RIP-like mutations on the supernumerary chromosomes. Three elements that have RIP-like copies on the supernumerary genome. The intact element is compared with RIPped copies on the core chromosomes, and “RIP-like copies” (unknown) on the supernumerary genome. For the third element, there is no intact in the genome. The total number of transitions and transversions compared to a reference (ref) is given, as welll as the transition/transversion ratio, which is lower for the “RIP-like copies” on the supernumerary genome. (XLSX 11 kb) Additional file 9: Intact elements on the core chromosomes of isolate 2516 are not in the same location in the other isolates. Integration of a RLG_*Maggy* element in chromosome 4 of isolate 2516. Location of the TE is shown in the RepeatMasker track. Mapping of the HiSeq reads of isolates 2516, 2548, 7555 and bfb0173 is shown. This TE is not present at the same location in the other isolates. In isolate 2548 and 7555, no reads span the borders of the element (but the element is present in other locations in the genomes of isolates 2548 and 7555, and therefore reads for this sequence do exist). In isolate bfb0173, RLG_Maggy is not present at all. Yellow indicates that the reads could have mapped to other places in the genome of isolate 2516 as well. (DOCX 768 kb) Additional file 10: TE integration and genome coverage on three supernumerary contigs. Integration of intact TEs on supernumerary contigs 668, 561 and 550. The graphs shows in a sliding 1 kb window the fraction of bases from the reference contig that is covered by HiSeq reads of every isolate (value between 0 and 1). The upper track shows all TEs on contig 668, 561 and 550 of isolate 2516 that are >1 kb and >90 % identity to the element prototype (Additional file 5) with yellow dots. This TE landscape was used for comparison with isolates 2548, 7555 and bfb0173. Dots for these three isolates indicate elements for which there is read mapping that an element has integrated in the exact same location as the element in isolate 2516 (and is therefore ancestral). Dots that align vertically are conserved in multiple isolates. (DOCX 261 kb) Additional file 11: Non-cumulative integrations of TEs on the supernumerary genome. Four instances are shown where a transposable element is in the same location for isolate 2516 and one or more other isolates. Tracks from top to bottom: RepeatMasker output, HiSeq reads from isolate 2516, HiSeq reads from isolate 2548, HiSeq reads from isolate 7555, and HiSeq reads from isolate bfb0173. Reads that can map to more than one location in the genome are automatically colored yellow in CLC Genomics Workbench. First screenshot: a DTA_*Nymeria* element on contig 550 is shared between 2516, 2548 and 7555. Second screenshot: a DTF_*Fot2* element is shared between 2516, 2548 and 7555 on contig 550. Third screenshot: A DTF_*Fot3-B* element has inserted into a DTA_*RLT2* element on contig 308. The DTA_*RLT2* element is shared between 2516, 7555 and bfb0173. The insertion of DTF_*Fot3-B* is shared between 2516 and bfb0173. Fourth screenshot: a RLG_*Skippy* element on contig 308 is partially shared between 2516 and bfb0173 (only downstream flank has convincing read support). (DOCX 622 kb) Additional file 12: List of intact transposable elements on the core chromosomes. All 135 elements are listed with their coordinates and the environment that they inserted into. Low complexity is a low GC% region that is sometimes hard to distinguish from ancient RIP. RIP indicates that the element has inserted into a RIPped copy of a TE (and is also low GC%). (XLSX 17 kb) Additional file 13: List of 104 gene duplications on the supernumerary chromosomes. Gene identifier for the core and supernumerary gene(s) are given as well as the length of the AA similarity between them. Locations of the duplicated genes on the supernumerary genome are given. For isolates 2548, 7555 and bfb0173, the presence of the duplicated genes was assessed by HiSeq read mapping. “yes” indicates the same duplication is present, “no” indicates that it is absent. Parentheses indicate inconclusive read mapping. Finally the functional annotation of the gene on the core that was duplicated, is detailed. Likely double assemblies are commented upon in the final column. (XLSX 1881 kb) Additional file 14: Representation of both a genuine (top) and double assembled gene duplication (middle and bottom). Upper track in every panel: mapping of the SMRT reads. Second track: CDS annotations of the reference genome. Lower track: mapping of the HiSeq reads for the reference isolate. Upper panel: two identical genes (circled in black, g12962 and g12967) on contig 459, separated by 20 kb of sequence. Read mapping shows contiguous sequence without assembly mistakes. Middle and bottom panel: identical genes (circled in black) are present at respectively the end of contig 440 (at 17 kb of the 23 kb contig) and the beginning of contig 441 (at 6 kb into the contig). Note the untangling of the reads near the end of the contig 440, where the assembler presumably stalled. The environment of the “duplicated genes” is identical in both instances. This is likely a case of double assembly. (DOCX 408 kb) Additional file 15: Supporting methods. (DOCX 18 kb) Additional file 16: Strategy for the detection of the supernumerary sequence insertions in chromosome three. A: Gel electrophoresis and PCR schematic for insertion 1 at 115-319 kb of chromosome 3. A single amplicon of 1010 bp is formed in the absence of the insertion (primers INS1-FLANK-fwd + INS1-FLANK-rev). When the insertion is present, two amplicons of 848 bp (INS1-FLANK-fwd + INS1-BLOCK-rev) and 1158 bp (INS1-BLOCK-fwd + INS1-FLANK-rev) are formed. An impression of the PCR schematic is given, the principle is the same for both insertions. B: Gel electrophoresis for insertion 2 at 883-1.348 kb of chromosome 3. A single amplicon of 1213 bp is formed in the absence of the insertion (primers INS2-FLANK-fwd + INS2-FLANK-rev). When the insertion is present, two amplicons of 932 bp (INS2-FLANK-fwd + INS2-BLOCK-rev) and 1294 bp (INS2-BLOCK-fwd + INS2-FLANK-rev) are formed. C: Gel electrophoresis of MAT1-1 (1203 bp) and MAT1-2 (859) diagnostic PCR. (DOCX 176 kb) Additional file 17: List of all primers used in this study, their sequence, reference and target. (XLSX 9 kb) Additional file 18: Centromeres of the four core chromosomes of isolate 2516. Hypothetical position of the centromeres of the four core chromosomes in *F. poae* isolate 2516 (denoted with black line). The positions of these regions that are low in GC%, coincide with low GC% regions in *F. graminearum*, where the presumed centromeres lie for that species. (DOCX 302 kb) Additional file 19: Molecular dating of TEs on the supernumerary genome, with outliers. Copies were aligned and branch lengths extracted from a maximum-likelihood phylogenetic tree. Branch lengths were used to calculate divergence times with a fixed substitution rate (1.05 * 10-9 substitutions per site per year). (DOCX 1068 kb)
