# Supplementary material for: Living apart together: crosstalk between the core and supernumerary genomes in a fungal plant pathogen
Source: BMC Genomics. 2016 Aug 23;17(1):670. doi: 10.1186/s12864-016-2941-6 (PMC4994206; doi:10.1186/s12864-016-2941-6)
Supplement: Additional file 1: — Fragmented BUSCOs in F. poae and F. graminearum. A: Fusarium poae predicted proteins that were identified as fragmented by the BUSCO analysis. The top track corresponds to the predicted gene model, the second track shows the predicted coding features and the bottom track shows the TopHat mapping of the RNAseq reads. Arrows indicate likely sites of miss-annotation (likely fusion of two separate genes). From top to bottom: g7865, g6381, g8721 and g6717. B: Predicted proteins in the F. graminearum set that were identified as fragmented by the BUSCO analysis. The top track corresponds to the predicted gene model, the second track shows the predicted coding features and the bottom track shows the TopHat mapping of the RNAseq reads. Arrows indicate likely sites of miss-annotation From top to bottom: FGRRES_16573 (likely fusion of two neighboring genes), FGRRES_10897 (likely addition of two exons), FGRRES_06268 (likely two exons missed). RNAseq data described in Zhao et al. [38] were used. C: Predicted proteins in the F. poae and F. graminearum set that were identified as fragmented by the BUSCO analysis and are shared between the two species. The top track corresponds to the predicted gene model, the second track shows the predicted coding features and the bottom track shows the TopHat mapping of the RNAseq reads. From top to bottom: g1567/FGRRES_05972, g1914/FGRRES_06308, g8796/FGRRES_09970. The first of the two visualizations corresponds to F. poae, the second corresponds to F. graminearum. (DOCX 754 kb) [file 12864_2016_2941_MOESM1_ESM.docx]

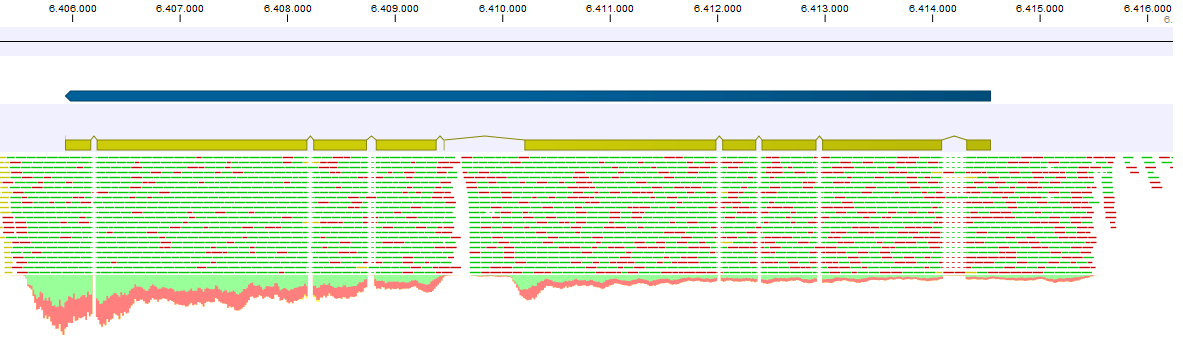

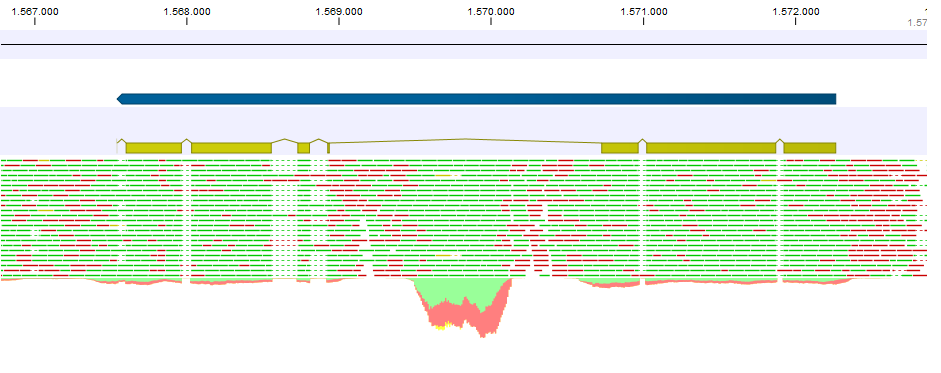

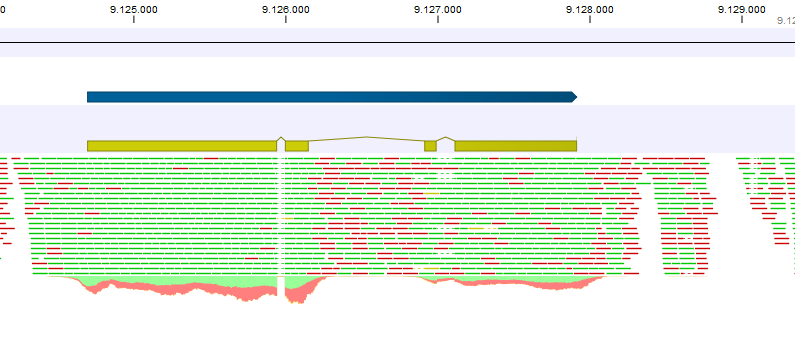

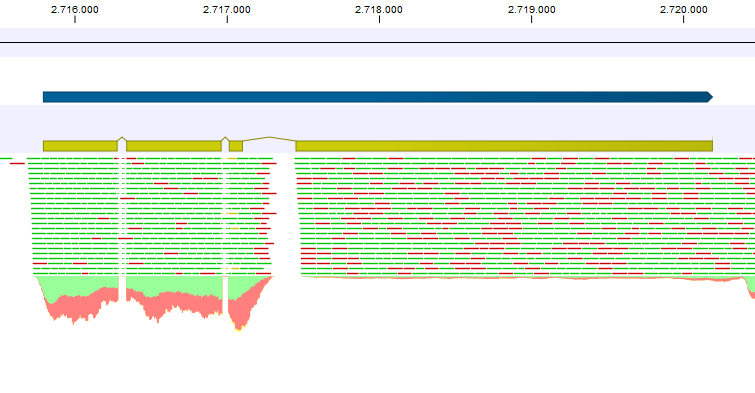
**Additional file 1** – **Fragmented BUSCOs in *F. poae* and *F. graminearum*. A:** *Fusarium poae* predicted proteins that were identified as fragmented by the BUSCO analysis. The top track corresponds to the predicted gene model, the second track shows the predicted coding features and the bottom track shows the TopHat mapping of the RNAseq reads. Arrows indicate likely sites of miss-annotation (likely fusion of two separate genes). From top to bottom: g7865, g6381, g8721 and g6717.

**A**


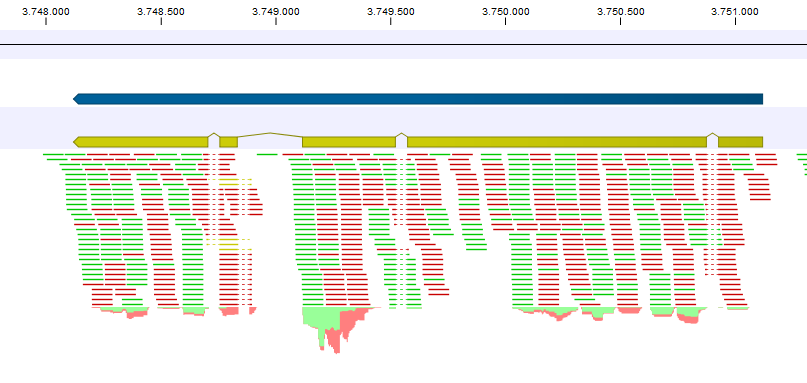

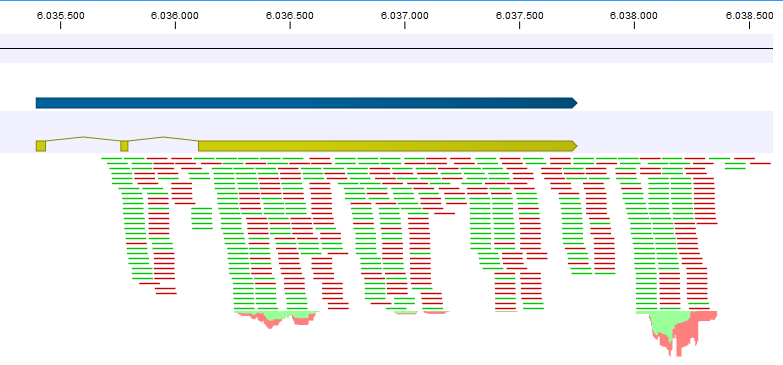

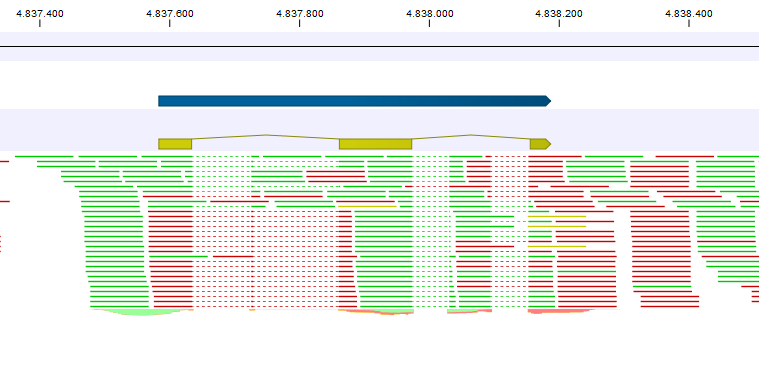


**B**

**Additional file 1** – **Fragmented BUSCOs in *F. poae* and *F. graminearum*. B:** Predicted proteins in the *F. graminearum* set that were identified as fragmented by the BUSCO analysis. The top track corresponds to the predicted gene model, the second track shows the predicted coding features and the bottom track shows the TopHat mapping of the RNAseq reads. Arrows indicate likely sites of miss-annotation From top to bottom: FGRRES_16573 (likely fusion of two neighboring genes), FGRRES_10897 (likely addition of two exons), FGRRES_06268 (likely two exons missed). RNAseq data described in Zhao et al. (2014) were used.


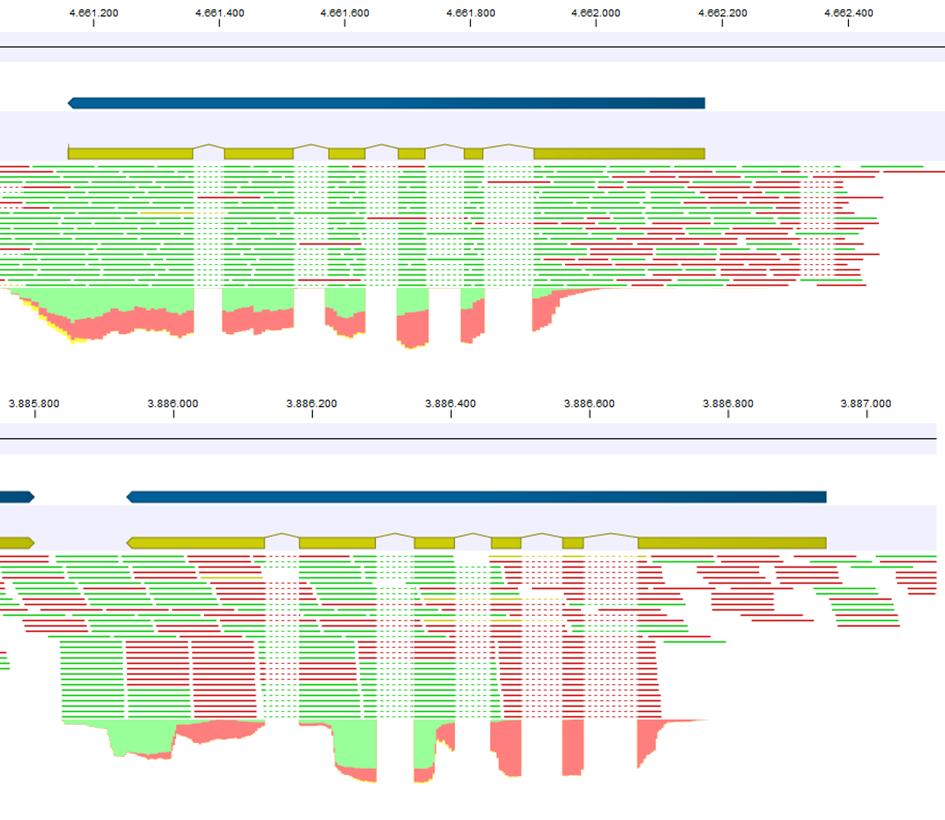


**C**


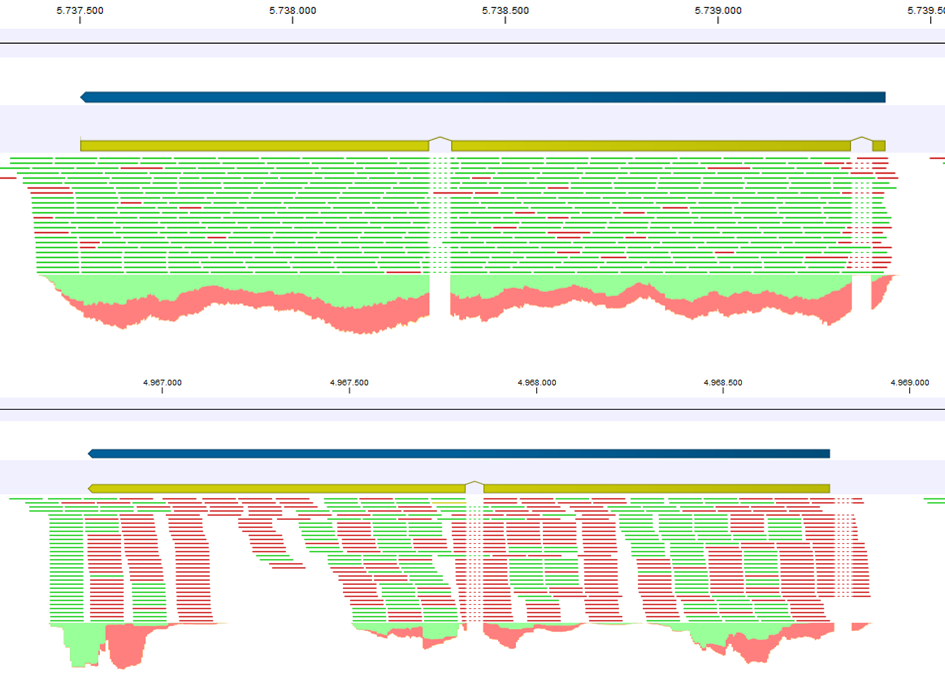


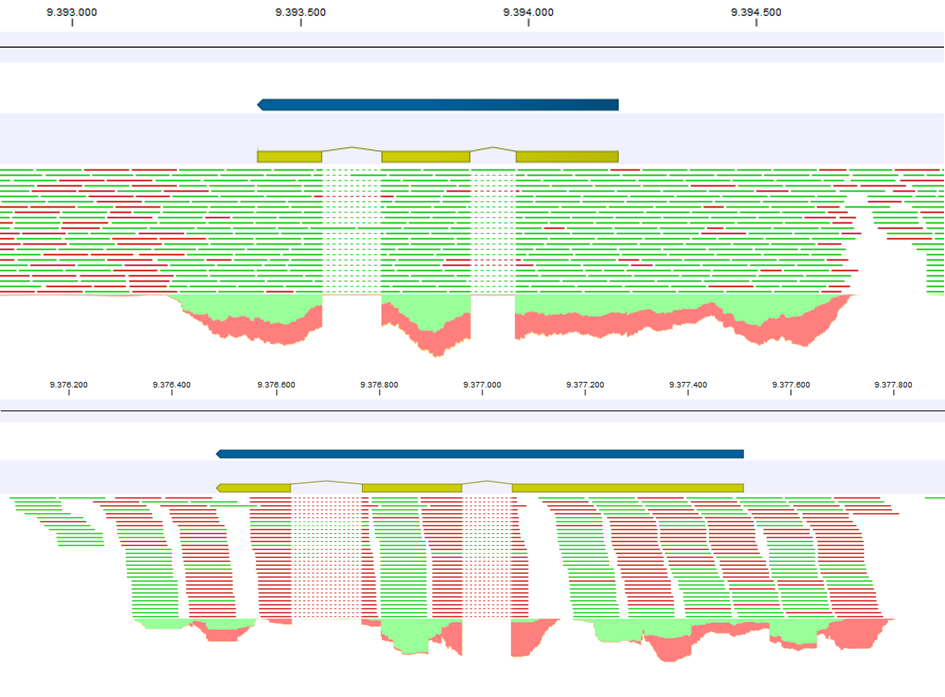


**Additional file 1** – **Fragmented BUSCOs in *F. poae* and *F. graminearum*. C:** Predicted proteins in the *F. poae* and *F. graminearum* set that were identified as fragmented by the BUSCO analysis and are shared between the two species. The top track corresponds to the predicted gene model, the second track shows the predicted coding features and the bottom track shows the TopHat mapping of the RNAseq reads. From top to bottom: g1567/FGRRES_05972, g1914/FGRRES_06308, g8796/FGRRES_09970. The first of the two visualizations corresponds to *F. poae*, the second corresponds to *F. graminearum*.
