# Supplementary material for: Living apart together: crosstalk between the core and supernumerary genomes in a fungal plant pathogen
Source: BMC Genomics. 2016 Aug 23;17(1):670. doi: 10.1186/s12864-016-2941-6 (PMC4994206; doi:10.1186/s12864-016-2941-6)
Supplement: Additional file 2: — Structural annotation and transcription of MAT1-1 and rid. A. Architecture of the MAT1 locus in F. poae isolate 2516, located at 3 120 000 bp into chromosome 2. The top track represents the predicted gene model, the second track represents the predicted coding features and the bottom track shows the TopHat mapping of the RNAseq reads. Note the correct splicing of introns for all three alleles. B. The rid (RIP defective) gene in F. poae isolate 2516, located at 2 232 000 bp into chromosome 2. The top track represents the predicted gene models, the second track represents the predicted coding features and the bottom track shows the TopHat mapping of the RNAseq reads. Two separate genes were predicted by the BRAKER1 pipeline. The F. pseudograminearum like gene model is superimposed as the single long coding feature. There is no splicing that supports this model under the conditions tested in this study. (DOCX 109 kb) [file 12864_2016_2941_MOESM2_ESM.docx]

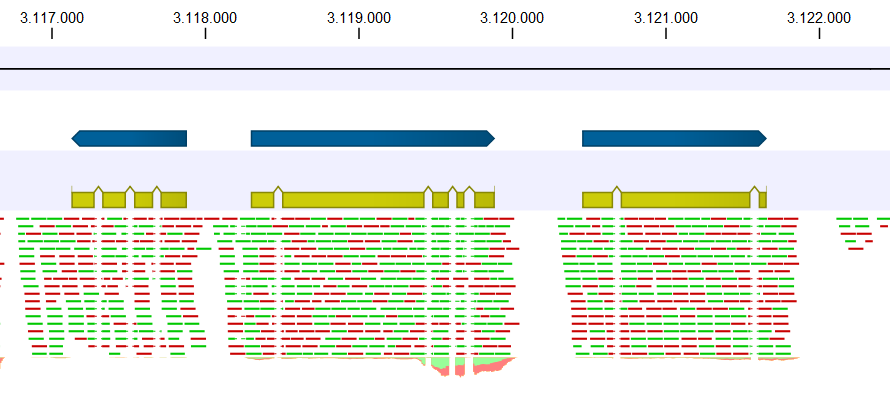


**A**


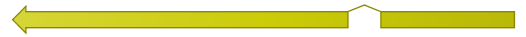

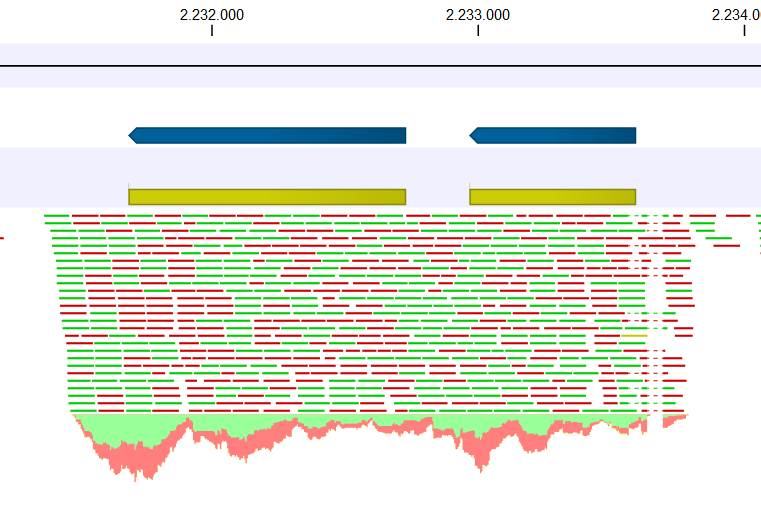


**B**

**Additional file 2: Structural annotation and transcription of MAT1-1 and rid. A.** Architecture of the MAT1 locus in *F. poae* isolate 2516, located at 3.120.000 bp into chromosome 2. The top track represents the predicted gene model, the second track represents the predicted coding features and the bottom track shows the TopHat mapping of the RNAseq reads. Note the correct splicing of introns for all three alleles. **B.** The *rid* (RIP defective) gene in *F. poae* isolate 2516, located at 2.232.000 bp into chromosome 2. The top track represents the predicted gene models, the second track represents the predicted coding features and the bottom track shows the TopHat mapping of the RNAseq reads. Two separate genes were predicted by the BRAKER1 pipeline. The *F. pseudograminearum* like gene model is superimposed as the single long coding feature. There is no splicing that supports this model under the conditions tested in this study.
