# Supplementary material for: Living apart together: crosstalk between the core and supernumerary genomes in a fungal plant pathogen
Source: BMC Genomics. 2016 Aug 23;17(1):670. doi: 10.1186/s12864-016-2941-6 (PMC4994206; doi:10.1186/s12864-016-2941-6)
Supplement: Additional file 6: — Transposable element datasheets. Information is listed for every transposable element family found in the genome of isolate 2516 that contains intact (not RIPped) elements. A: Tracks from top to bottom: LTR/TIR, coding region and mapping of RNAseq reads. B: Predicted protein used as bait for blastp, alignment of 15 best hits with the bait and neighbour-joining phylogenetic tree. For the retrotransposons, the pol protein was used. An asterisk denotes the Fusarium poae element in the tree. (PPTX 3451 kb) [file 12864_2016_2941_MOESM6_ESM.pptx]

## Slide 1
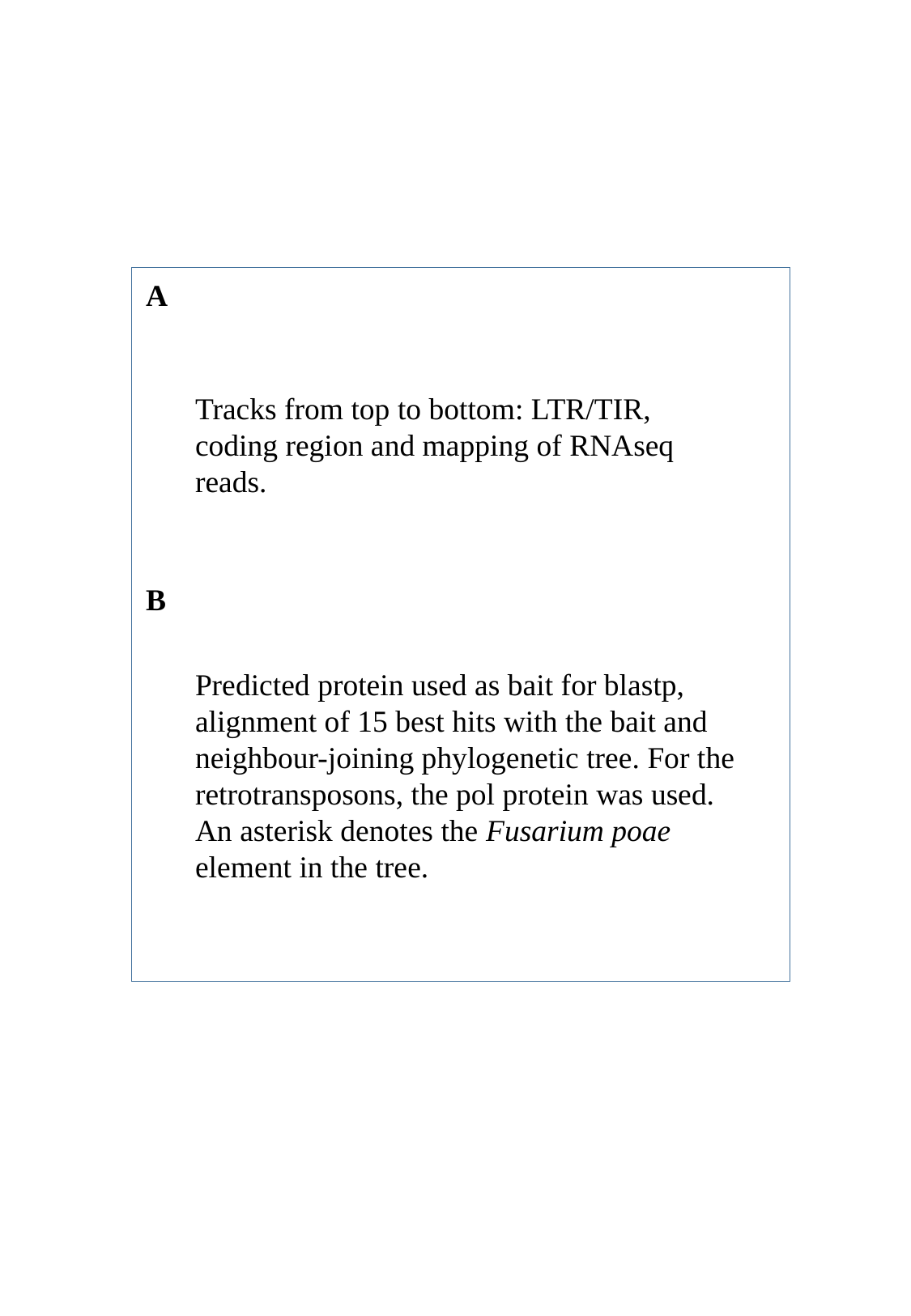

A
Tracks from top to bottom: LTR/TIR, coding region and mapping of RNAseq reads.
B
Predicted protein used as bait for blastp, alignment of 15 best hits with the bait and neighbour-joining phylogenetic tree. For the retrotransposons, the pol protein was used. An asterisk denotes the Fusarium poae element in the tree.

## Slide 2
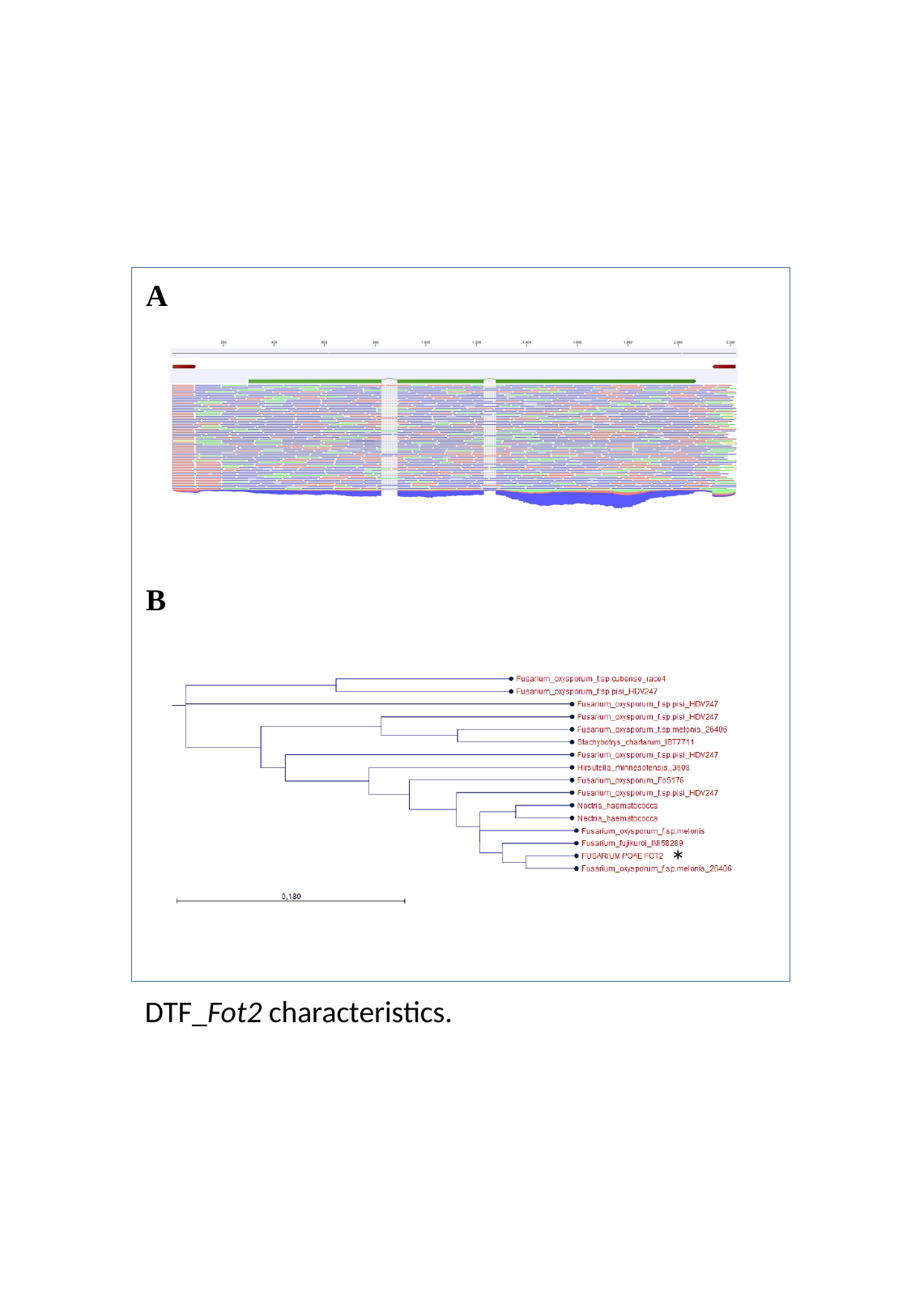

A
B
*
DTF_Fot2 characteristics.

## Slide 3
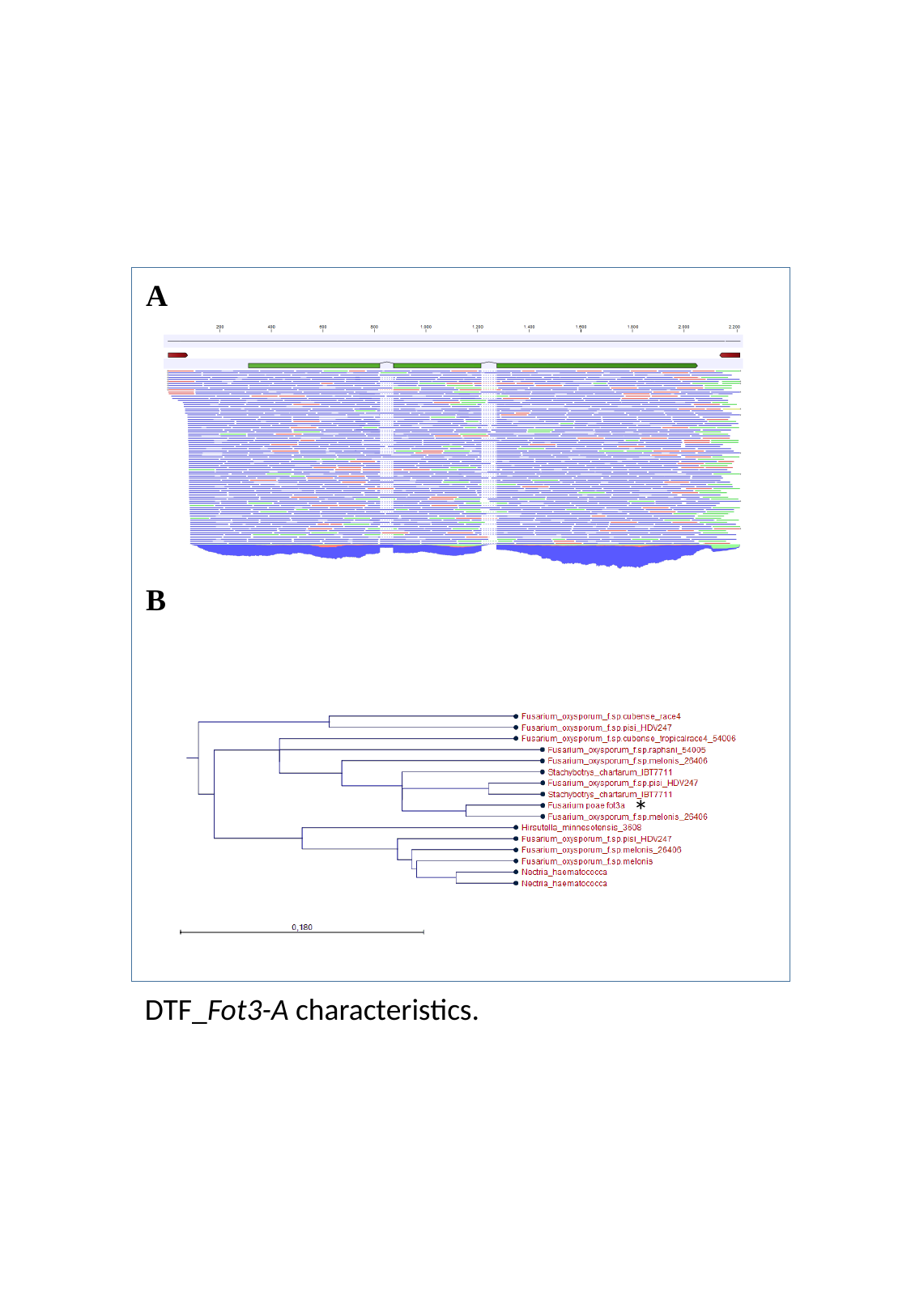

A
B
*
DTF_Fot3-A characteristics.

## Slide 4
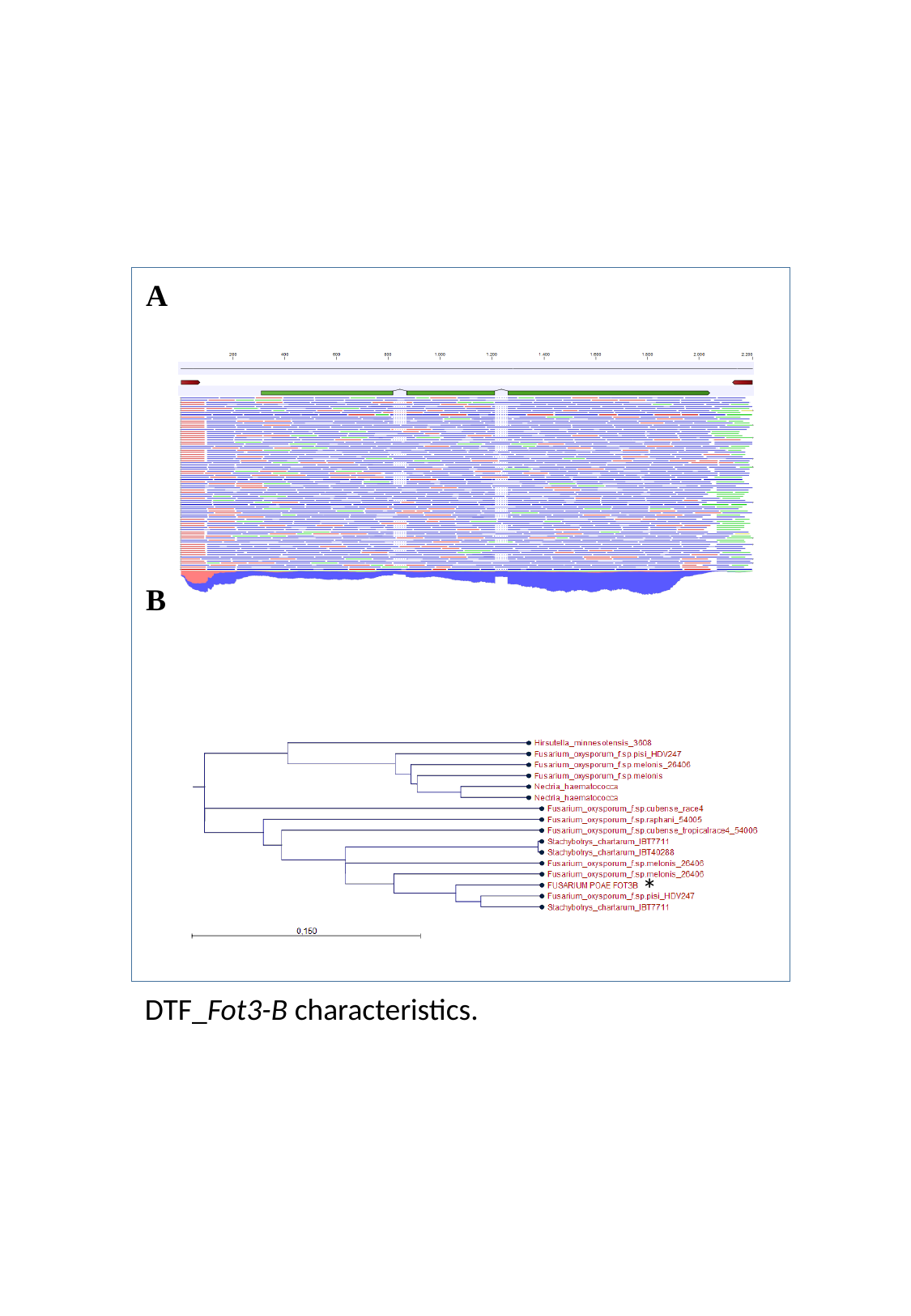

A
B
*
DTF_Fot3-B characteristics.

## Slide 5
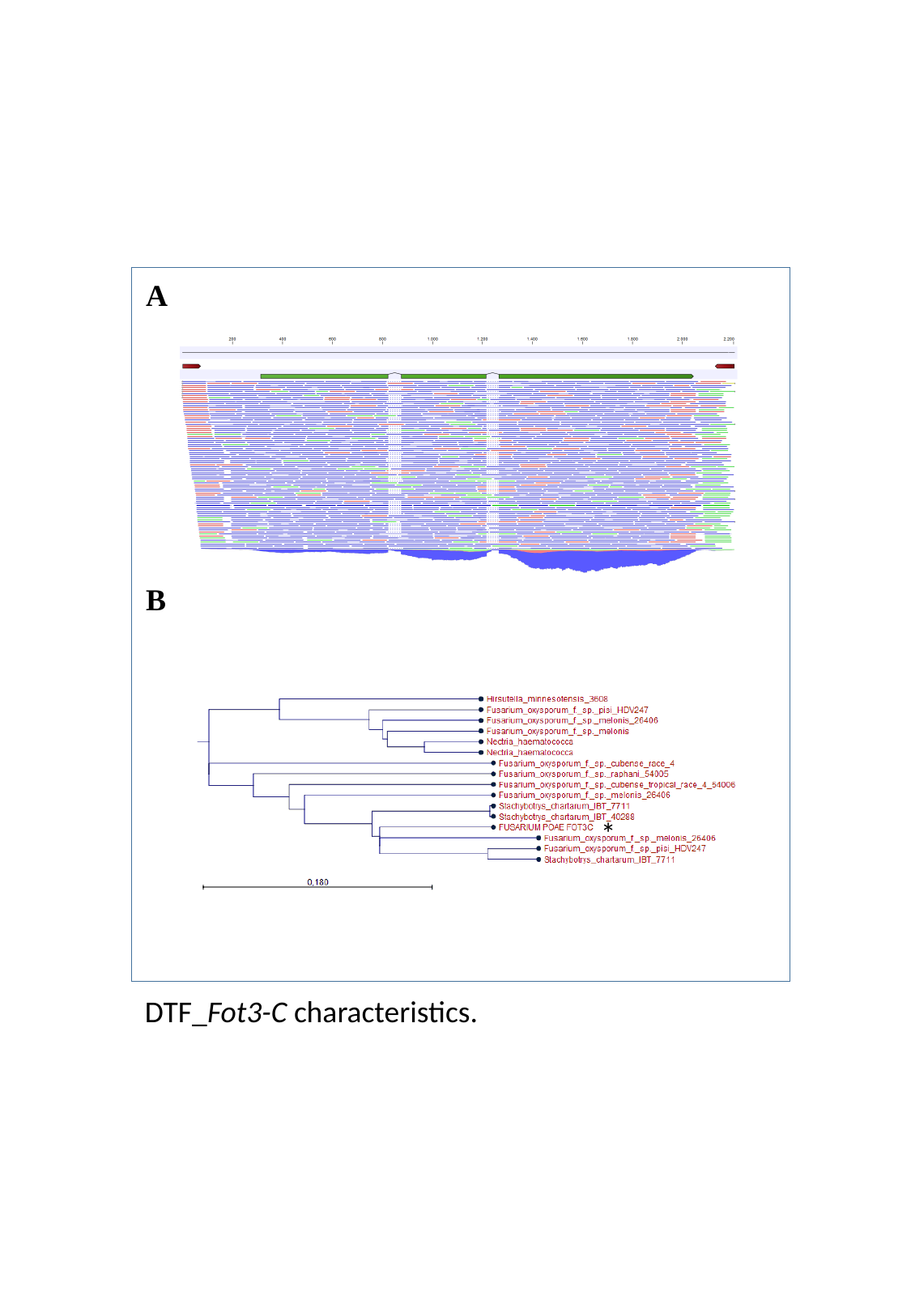

A
B
*
DTF_Fot3-C characteristics.

## Slide 6
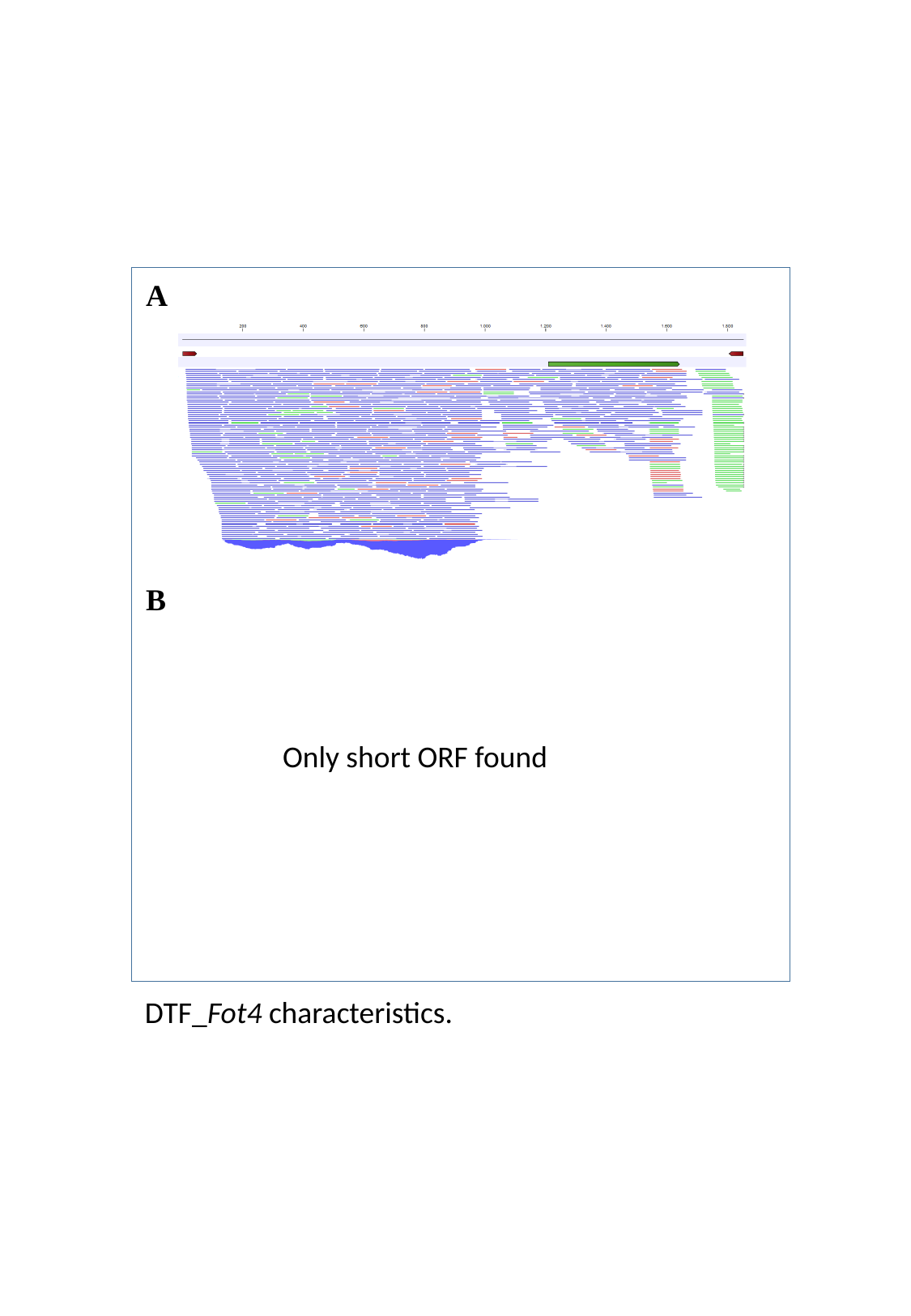

A
B
Only short ORF found
DTF_Fot4 characteristics.

## Slide 7
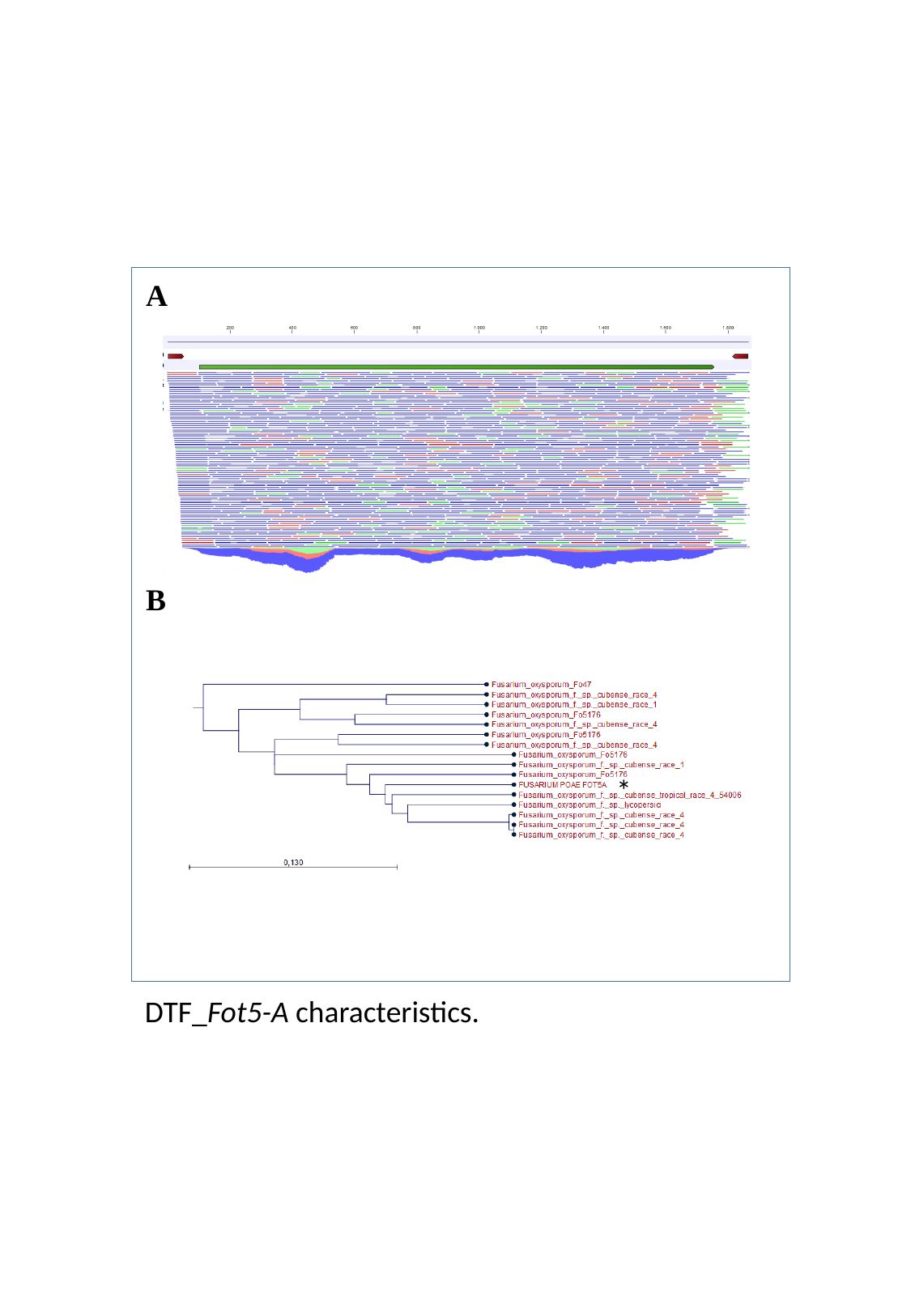

A
B
*
DTF_Fot5-A characteristics.

## Slide 8
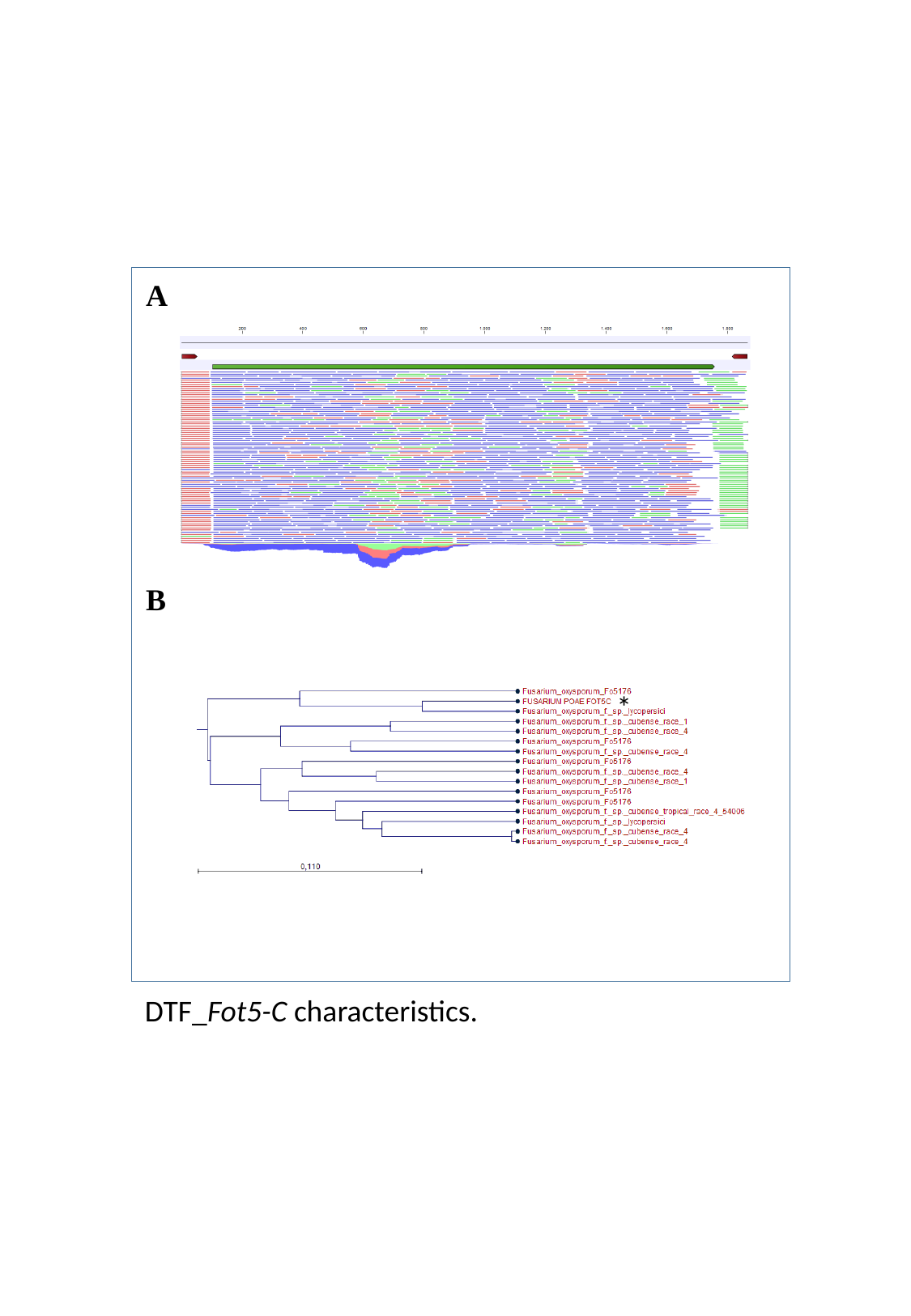

A
B
*
DTF_Fot5-C characteristics.

## Slide 9
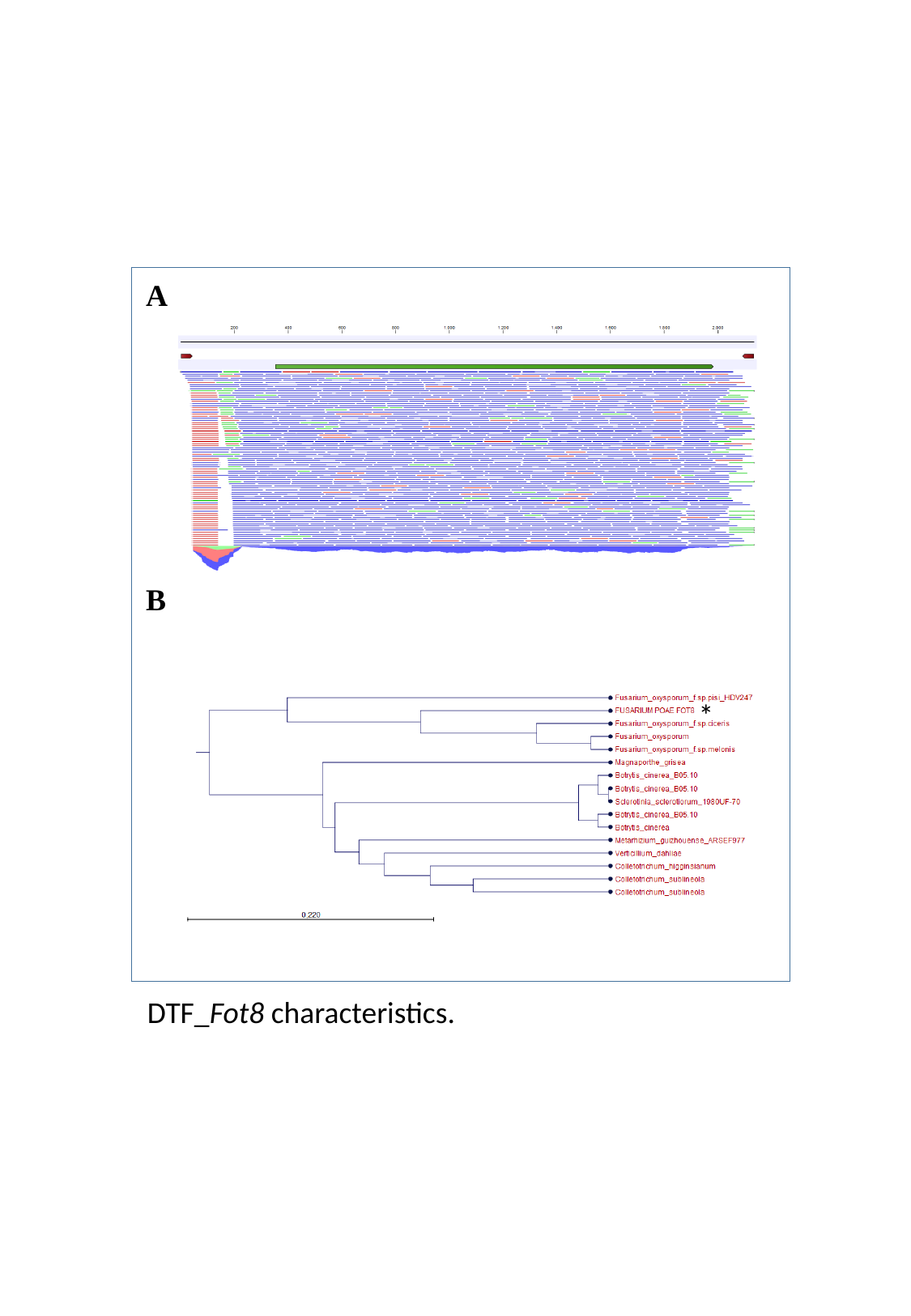

A
B
*
DTF_Fot8 characteristics.

## Slide 10
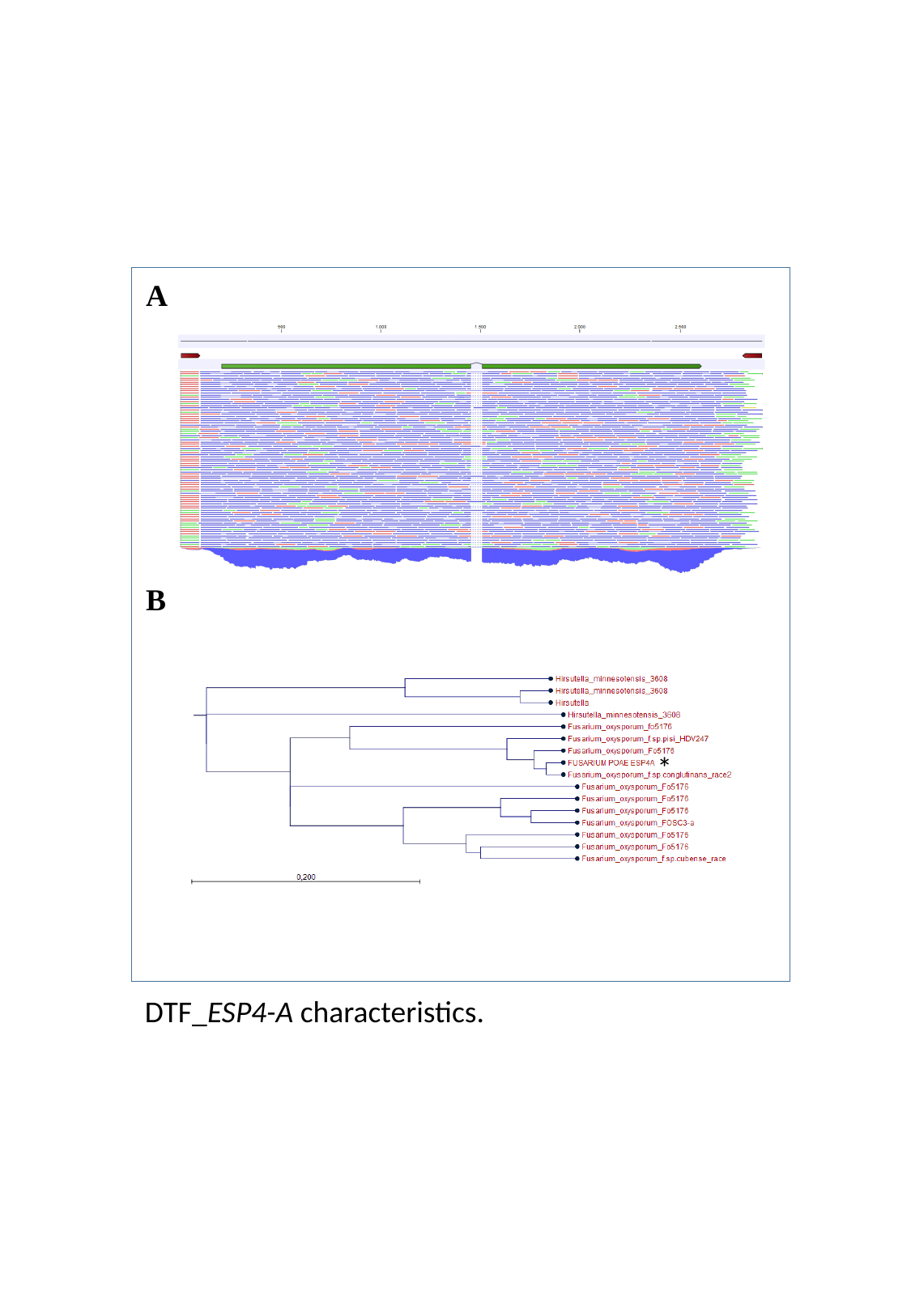

A
B
*
DTF_ESP4-A characteristics.

## Slide 11
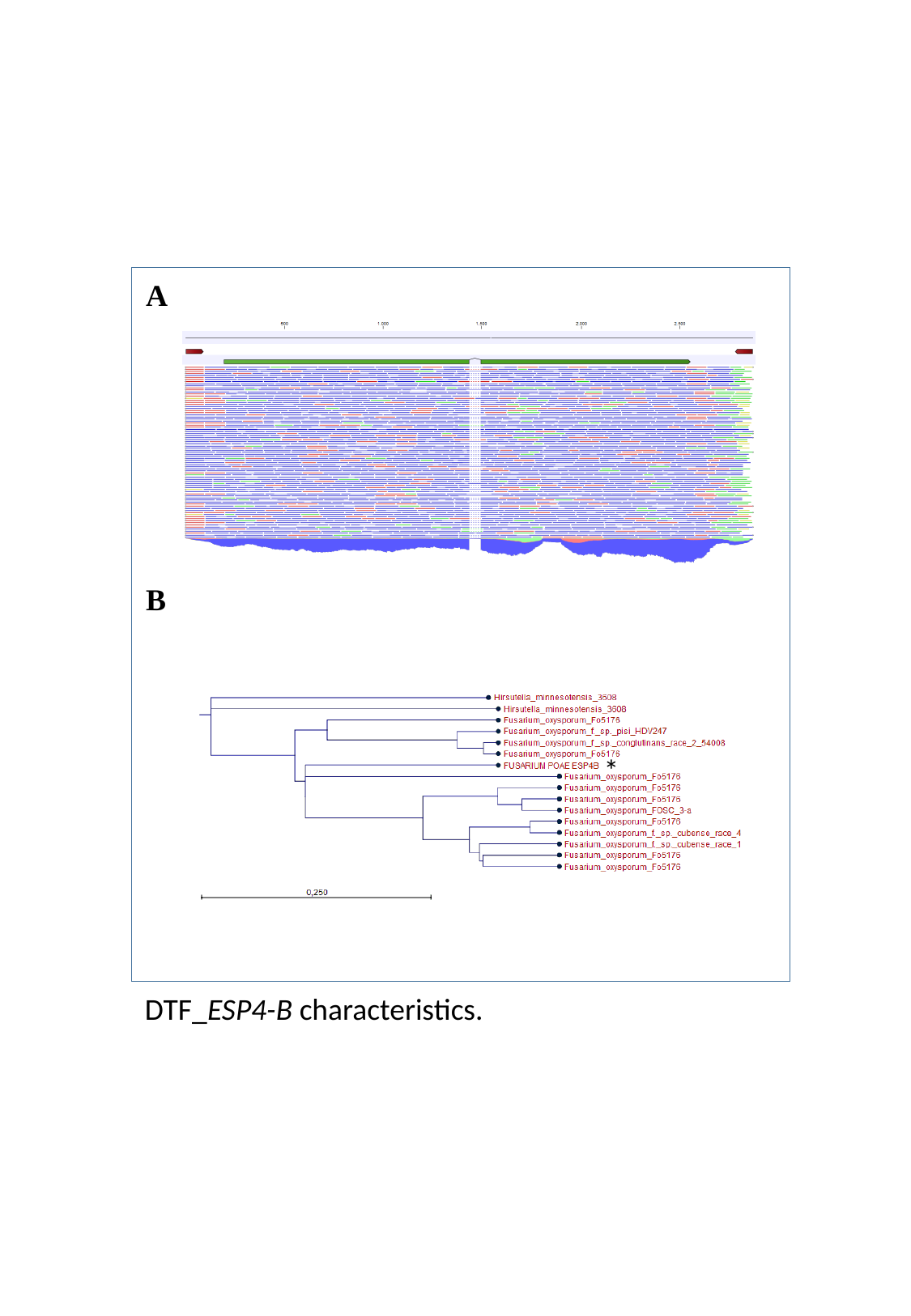

A
B
*
DTF_ESP4-B characteristics.

## Slide 12
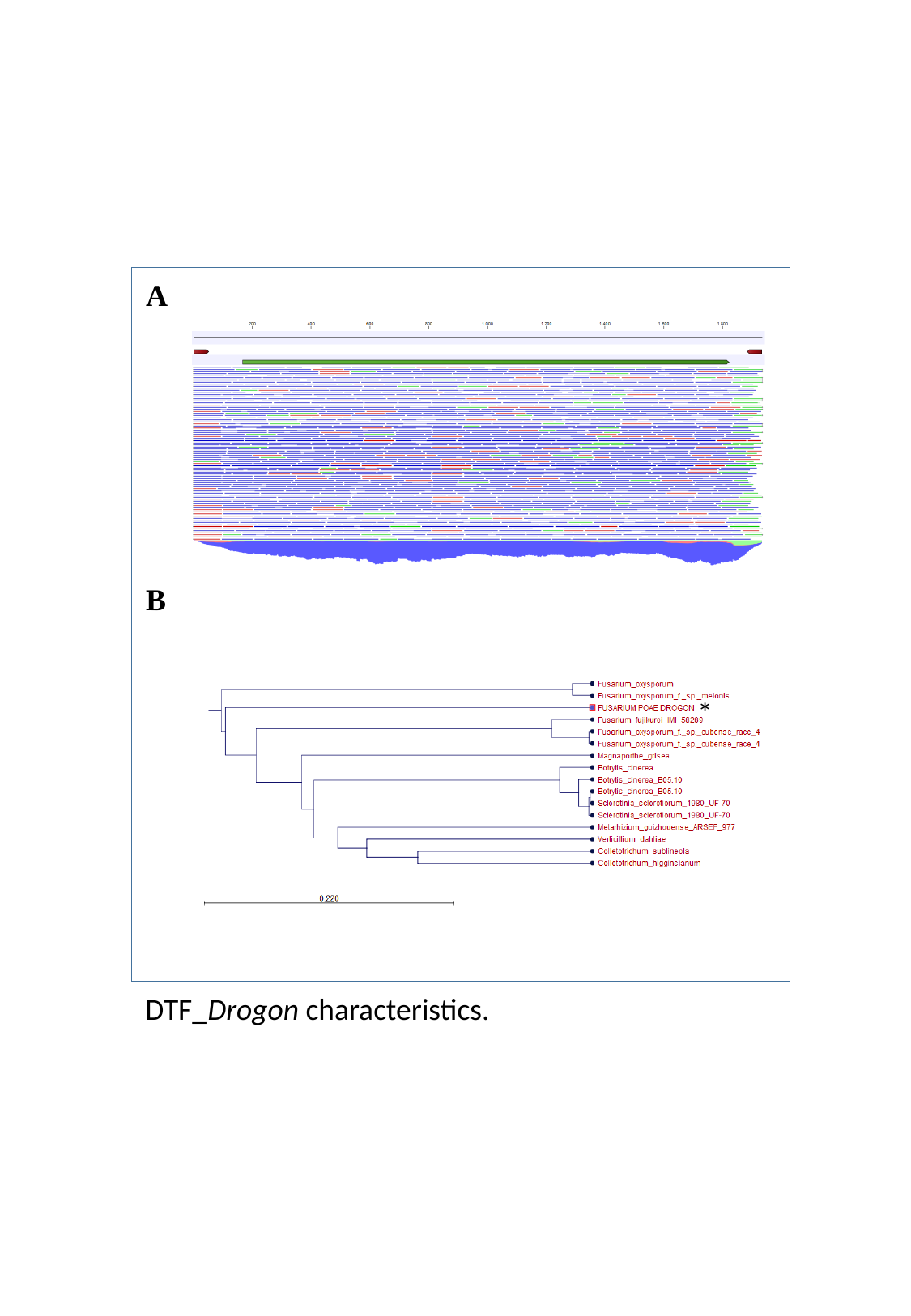

A
B
*
DTF_Drogon characteristics.

## Slide 13
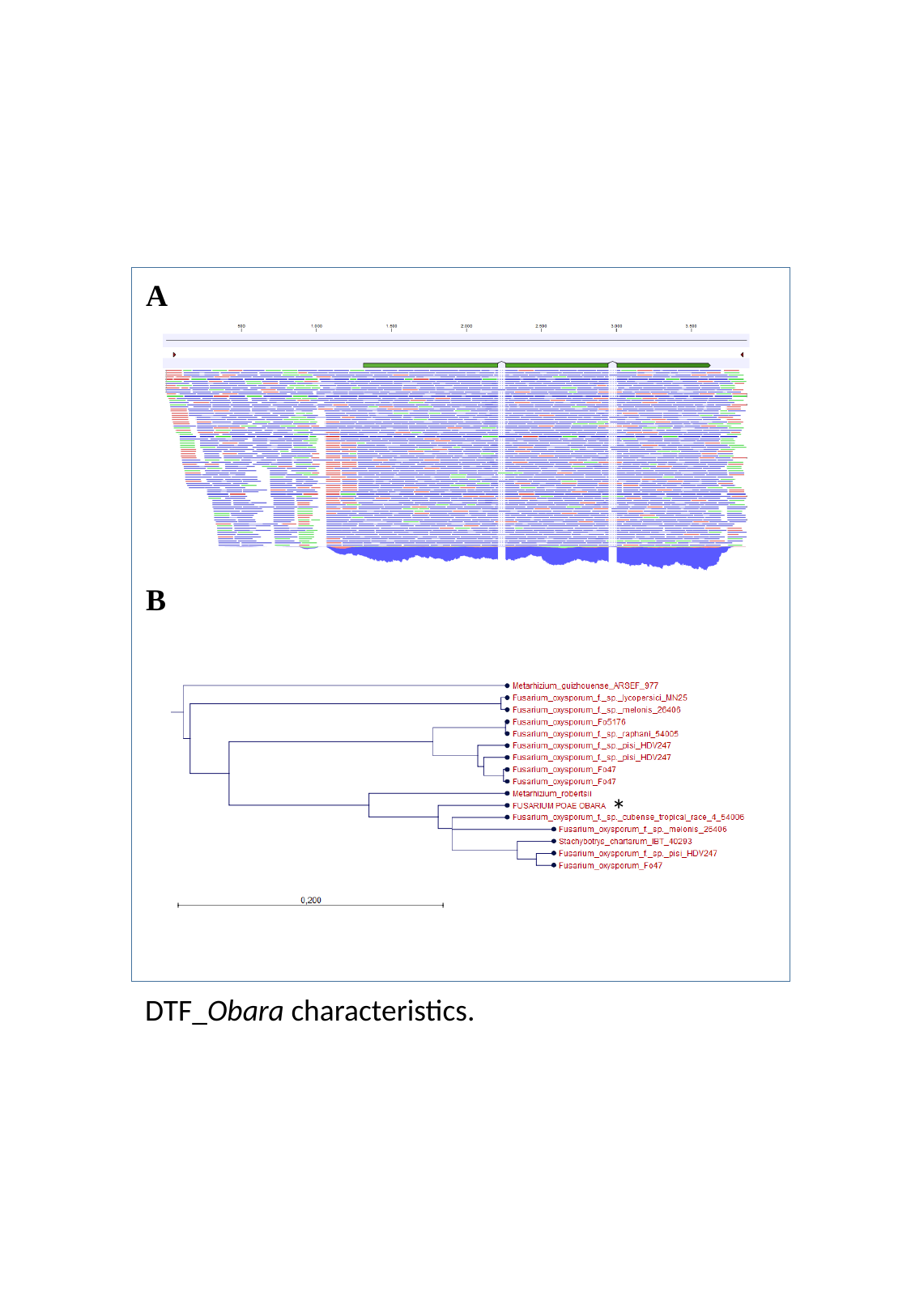

A
B
*
DTF_Obara characteristics.

## Slide 14
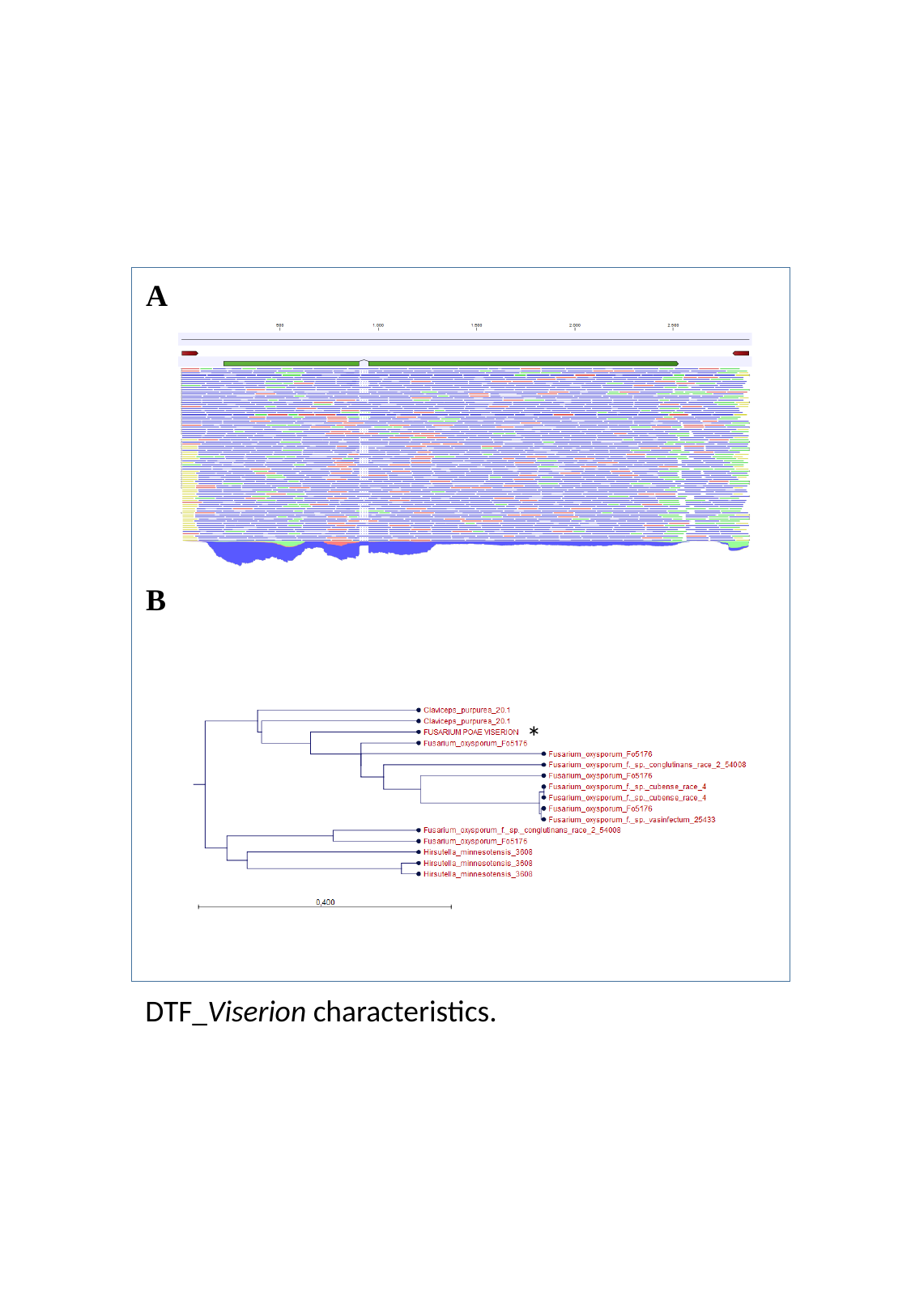

A
B
*
DTF_Viserion characteristics.

## Slide 15
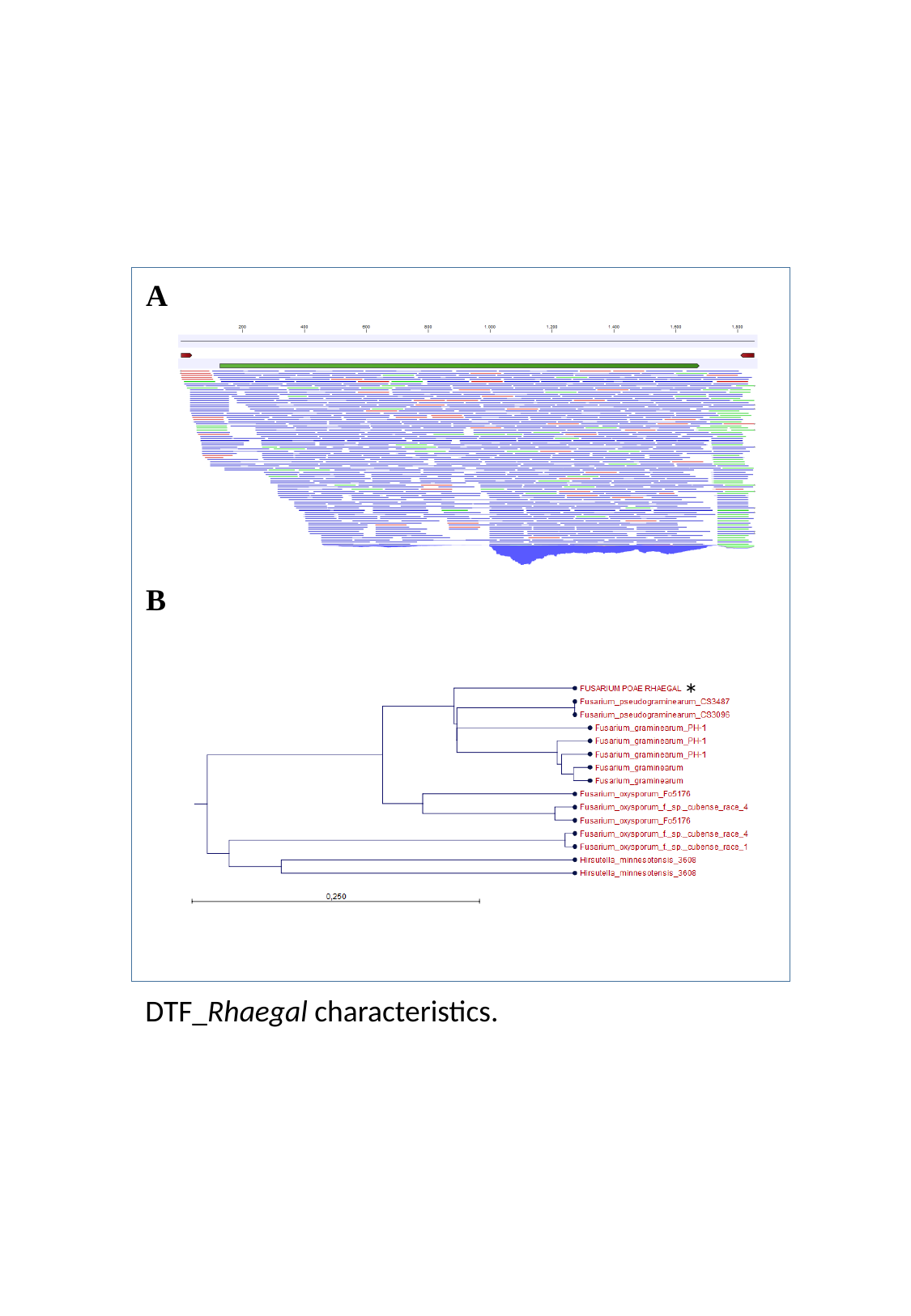

A
B
*
DTF_Rhaegal characteristics.

## Slide 16
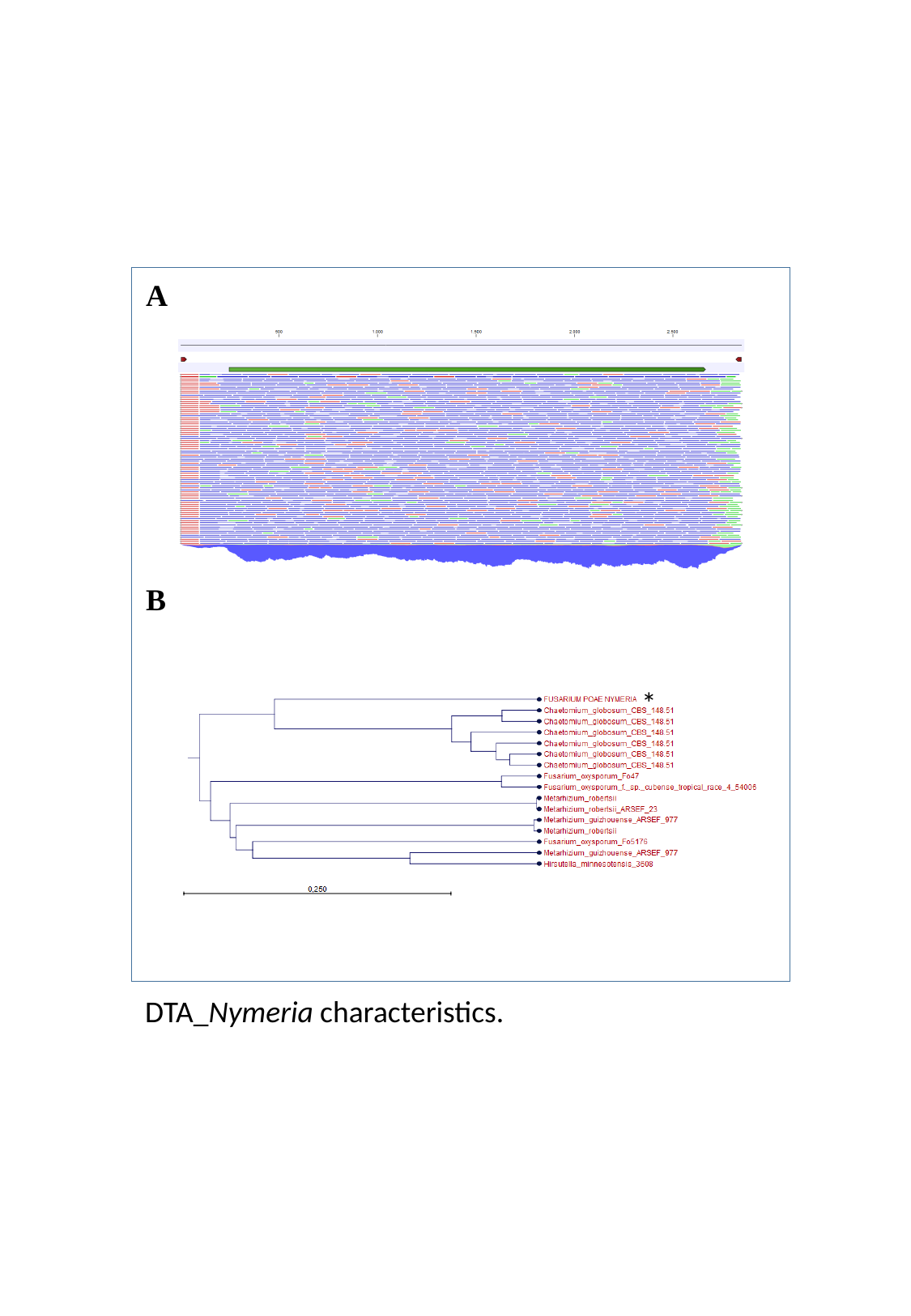

A
B
*
DTA_Nymeria characteristics.

## Slide 17
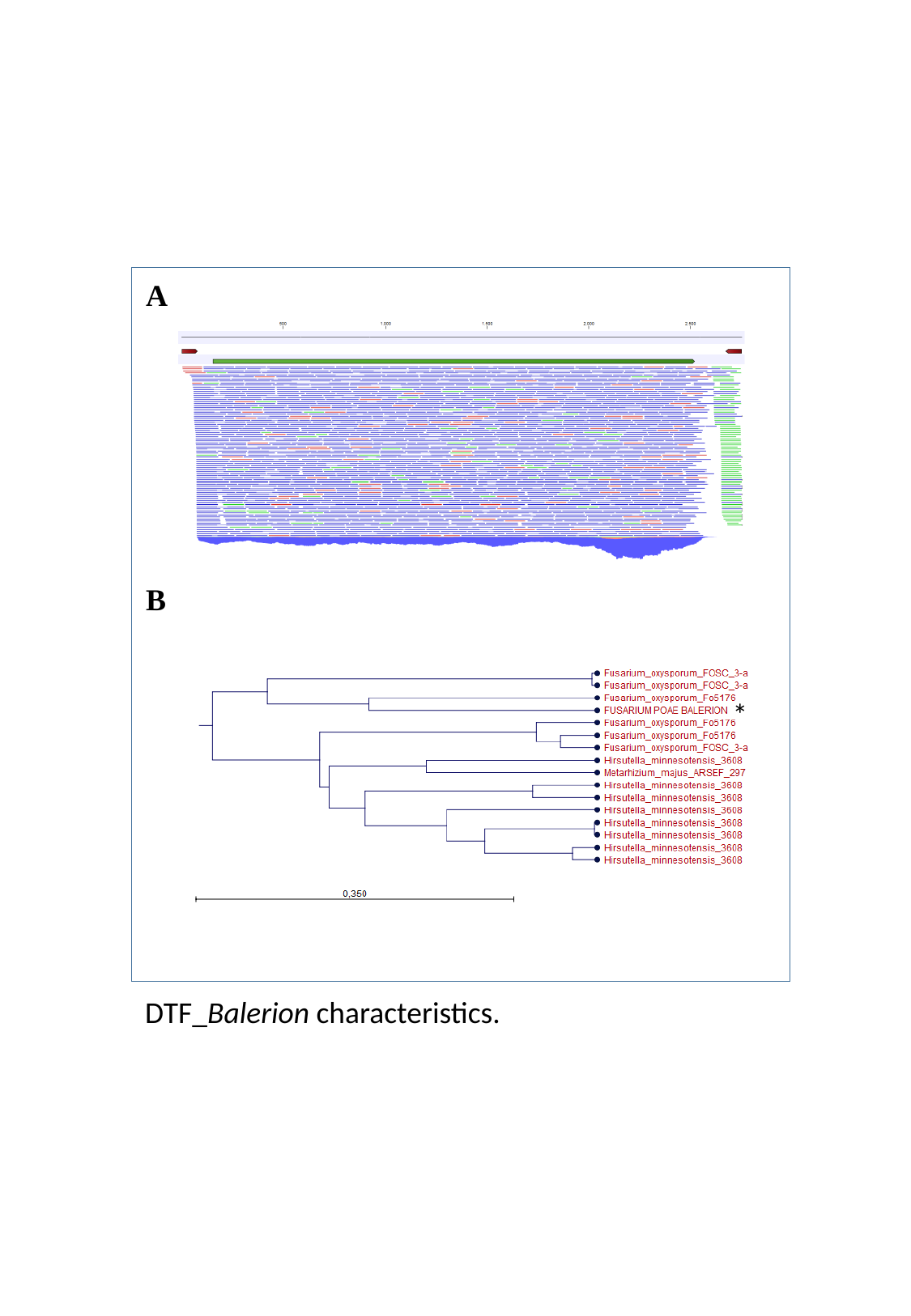

A
B
*
DTF_Balerion characteristics.

## Slide 18
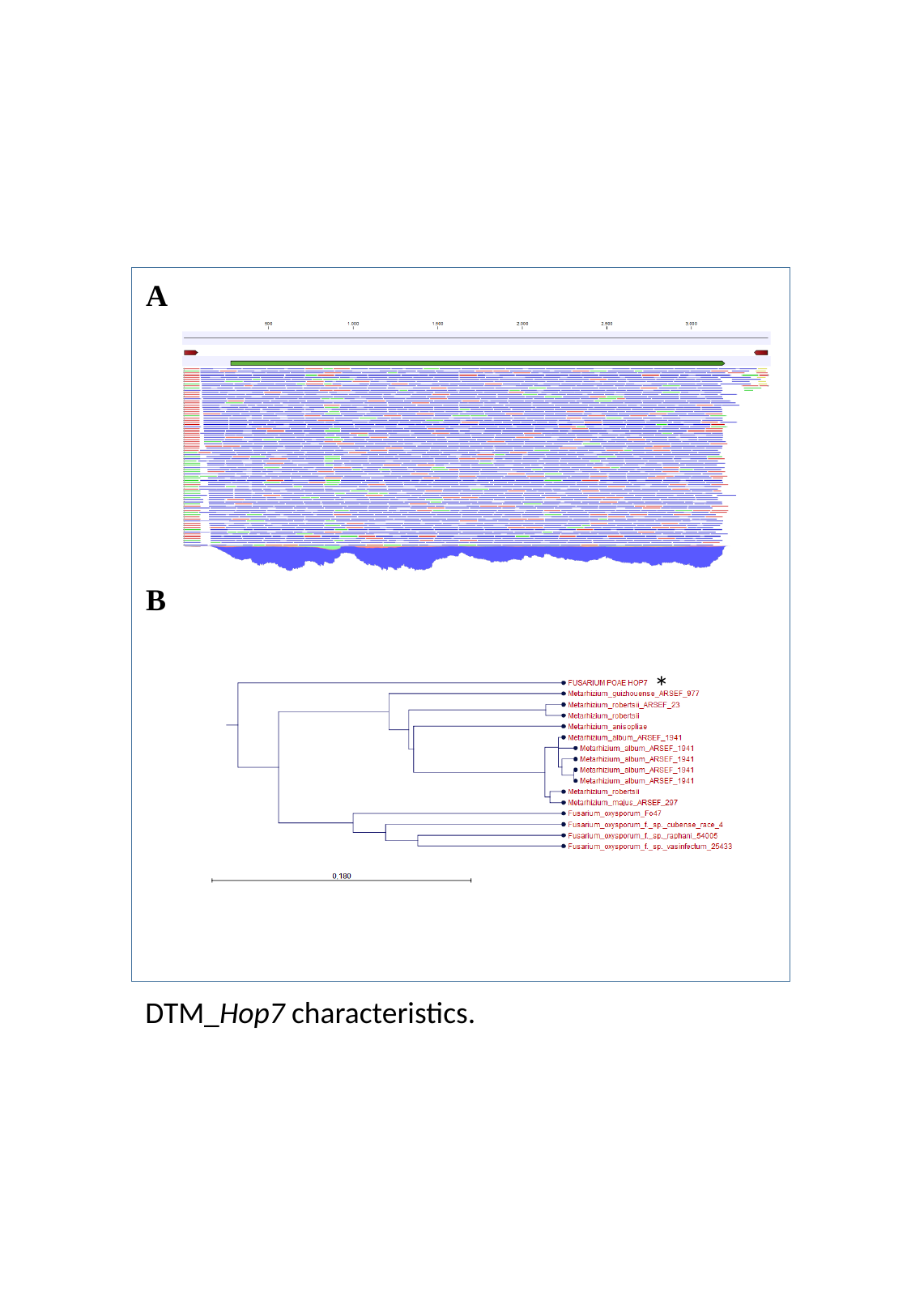

A
B
*
DTM_Hop7 characteristics.

## Slide 19
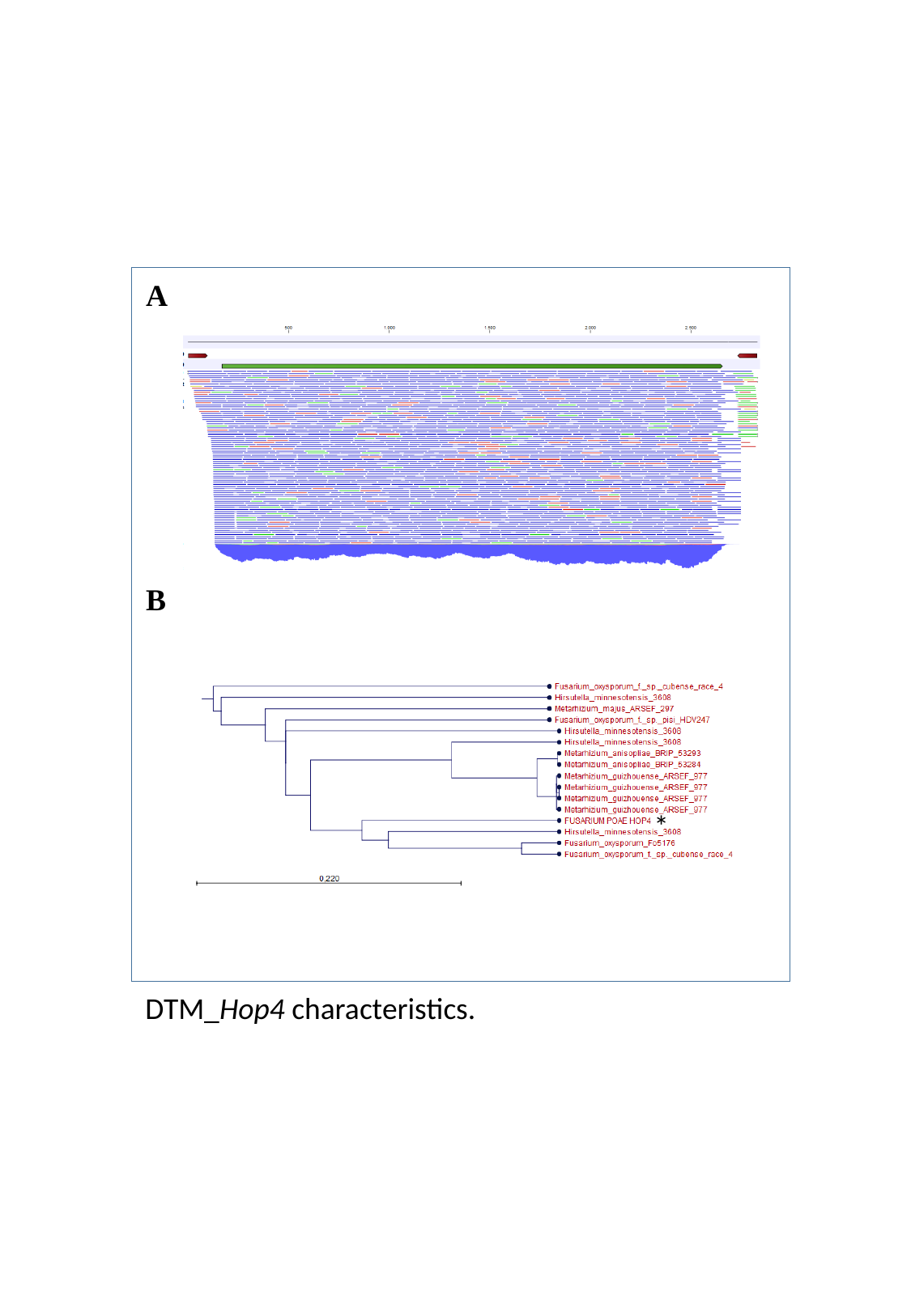

A
B
*
DTM_Hop4 characteristics.

## Slide 20
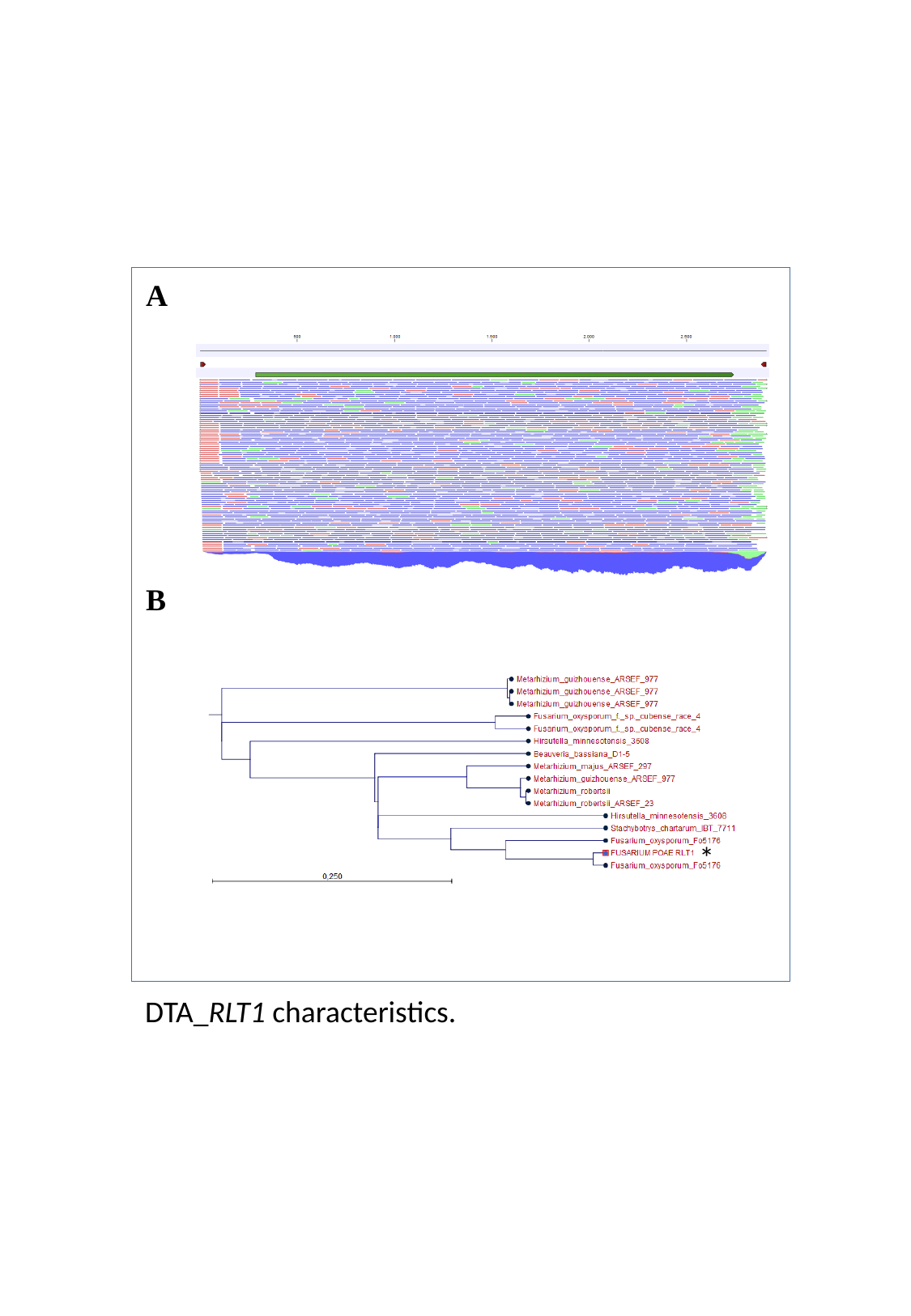

A
B
*
DTA_RLT1 characteristics.

## Slide 21
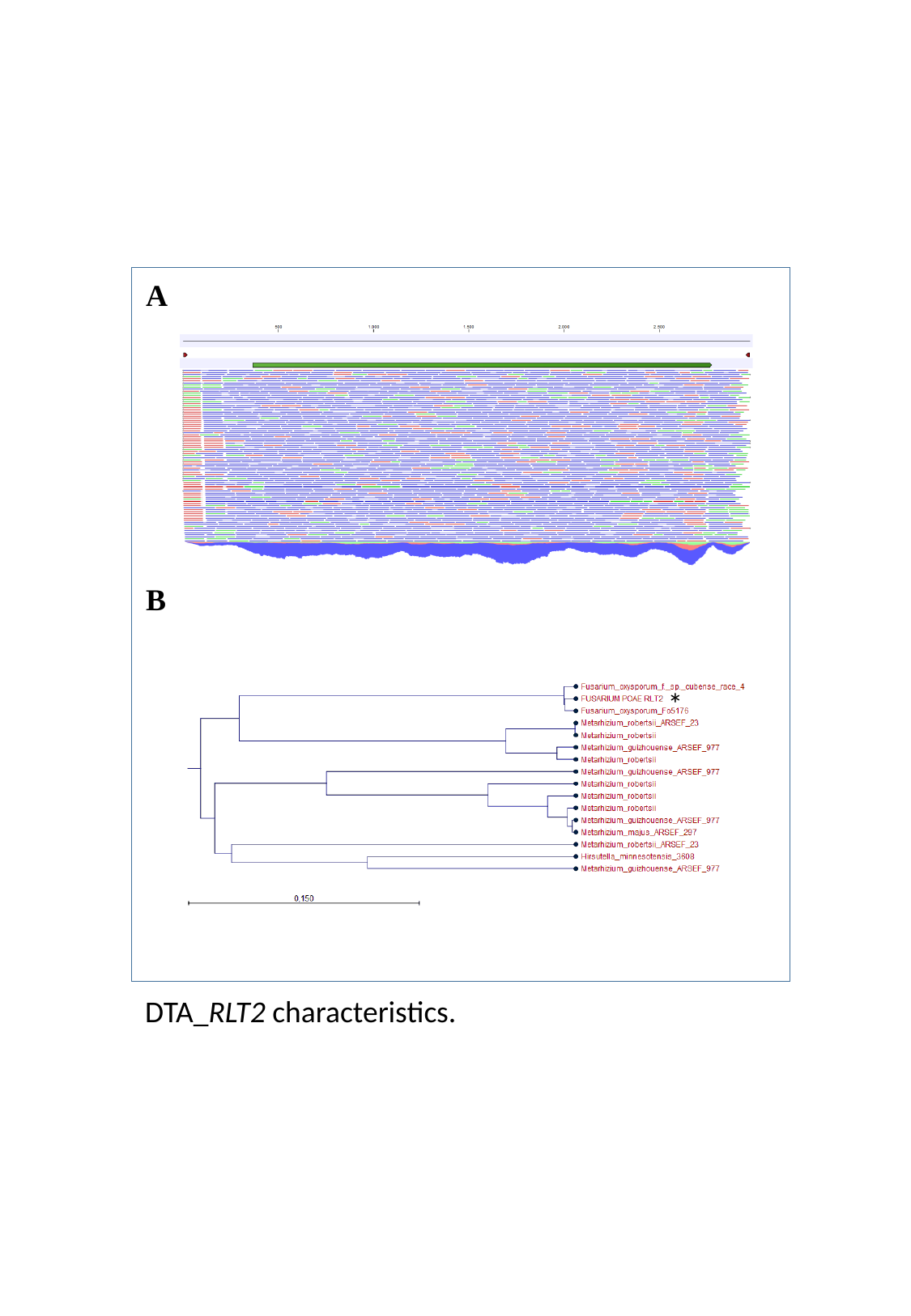

A
B
*
DTA_RLT2 characteristics.

## Slide 22
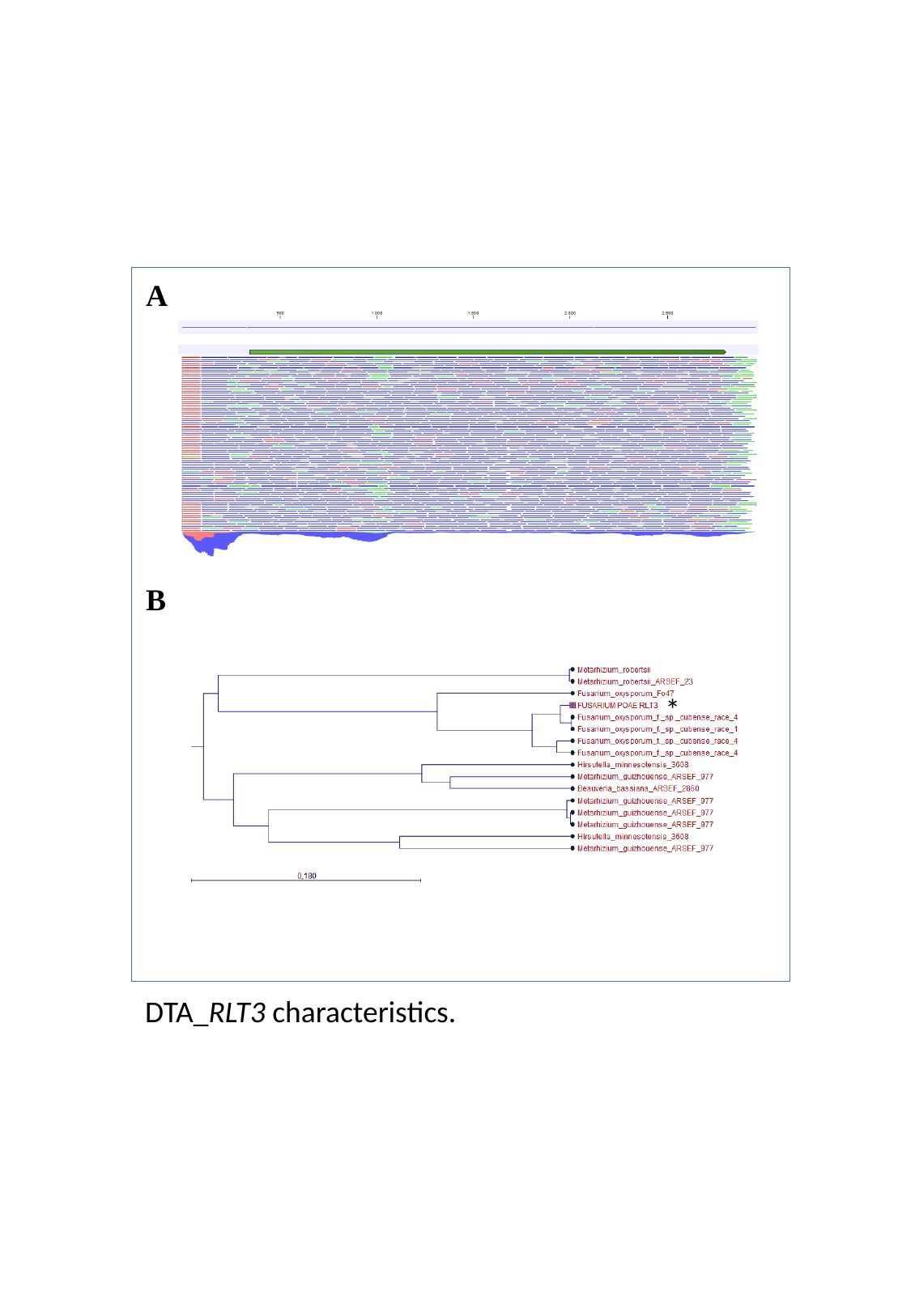

A
B
*
DTA_RLT3 characteristics.

## Slide 23
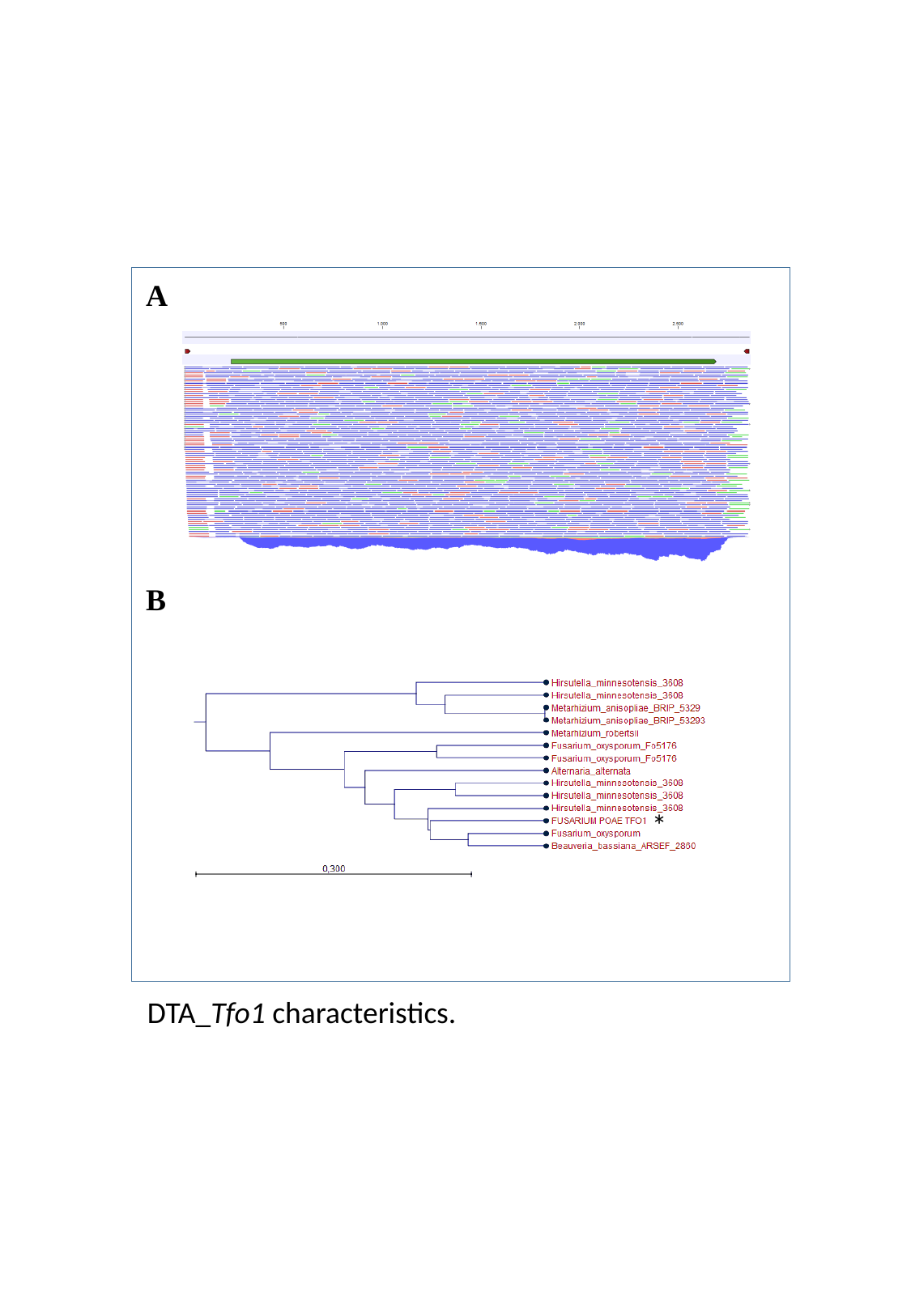

A
B
*
DTA_Tfo1 characteristics.

## Slide 24
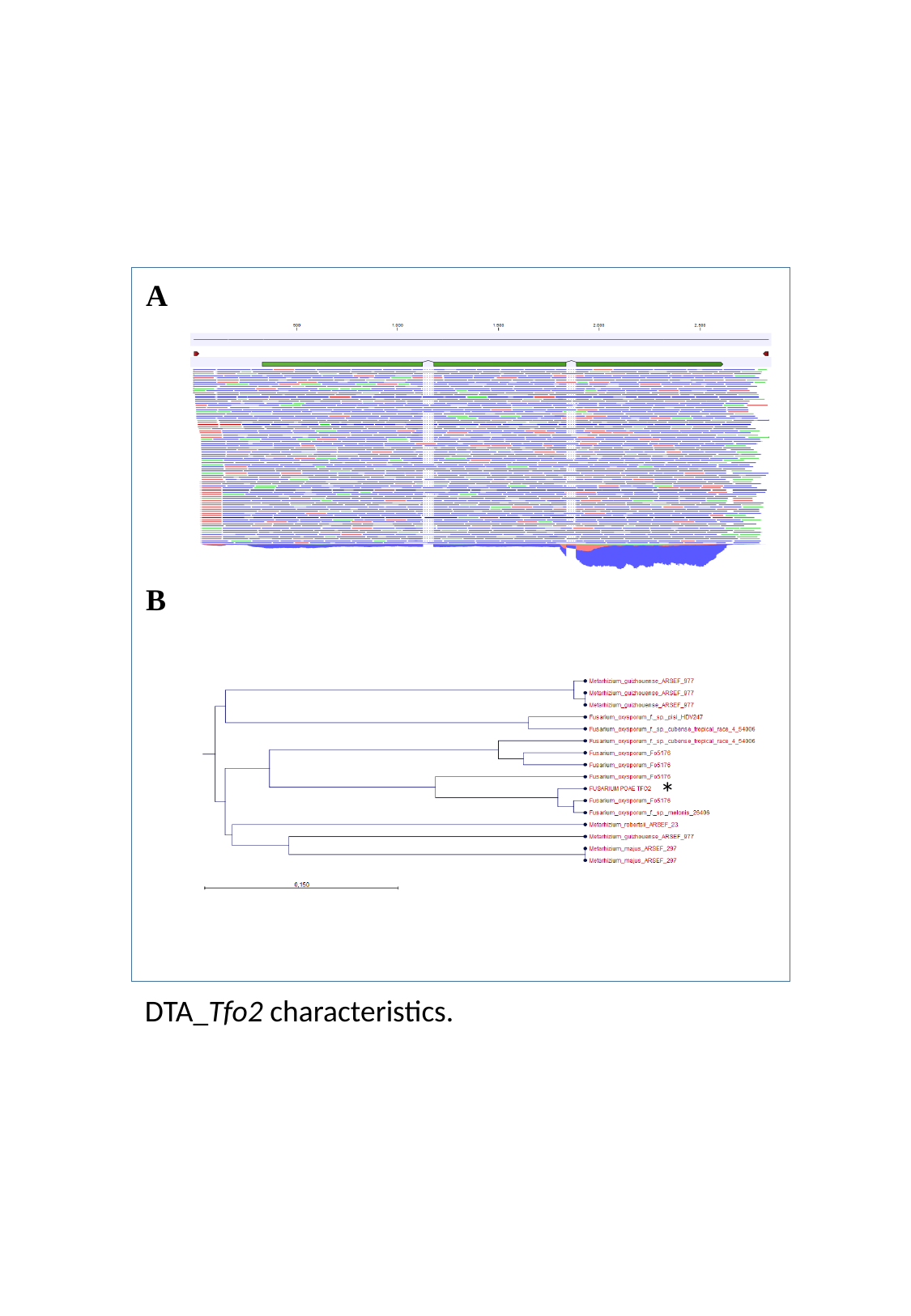

A
B
*
DTA_Tfo2 characteristics.

## Slide 25
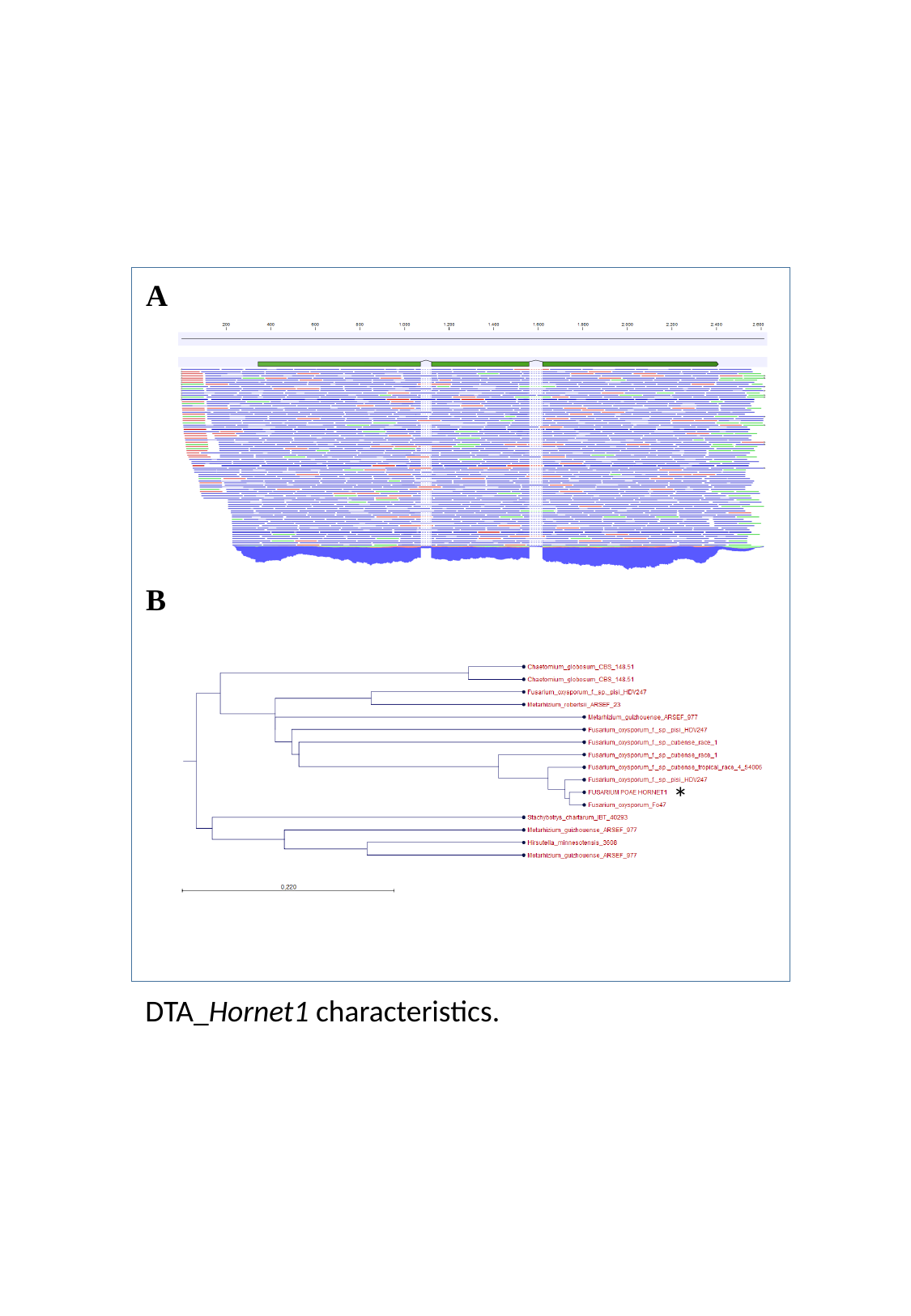

A
B
*
DTA_Hornet1 characteristics.

## Slide 26
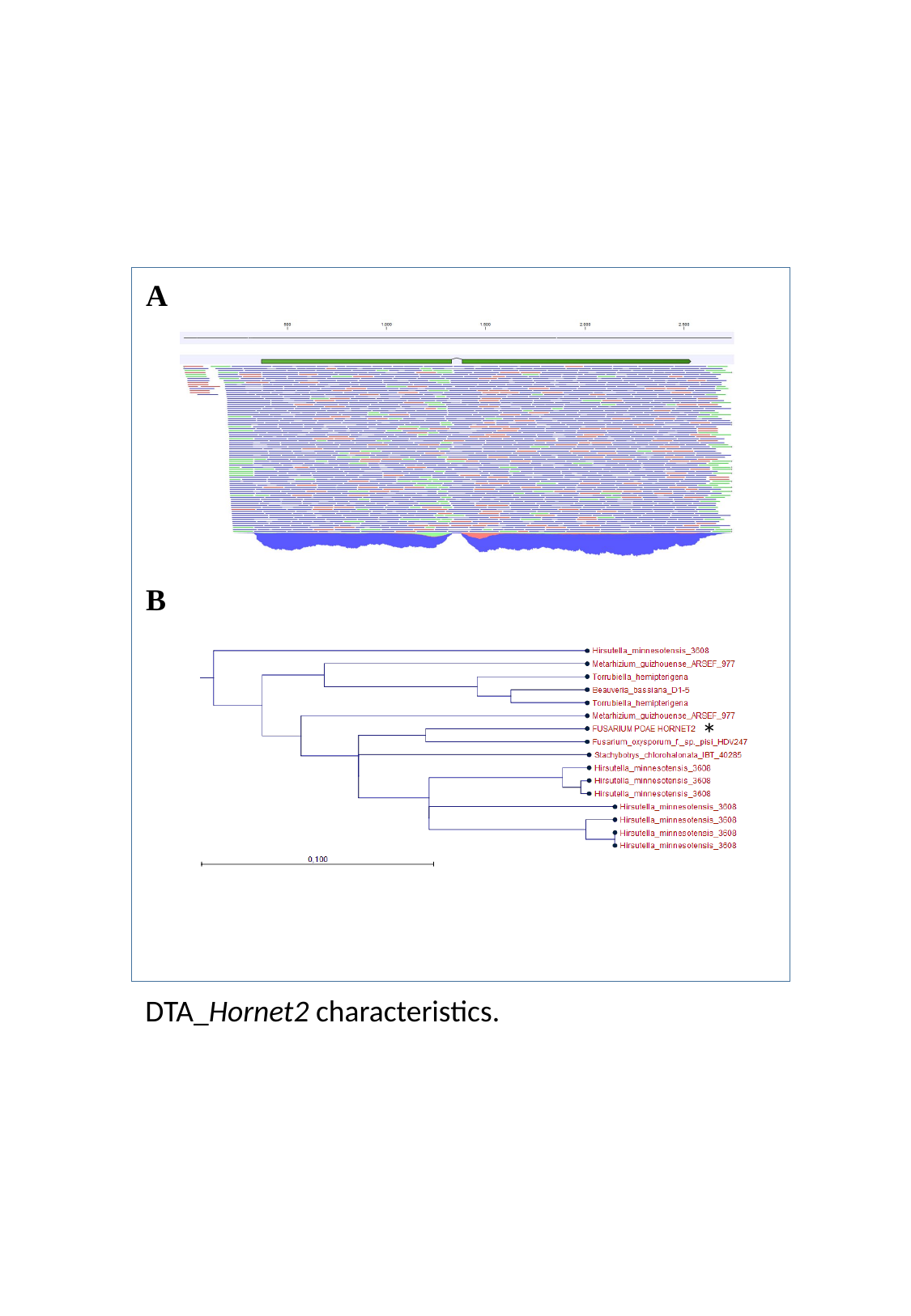

A
B
*
DTA_Hornet2 characteristics.

## Slide 27
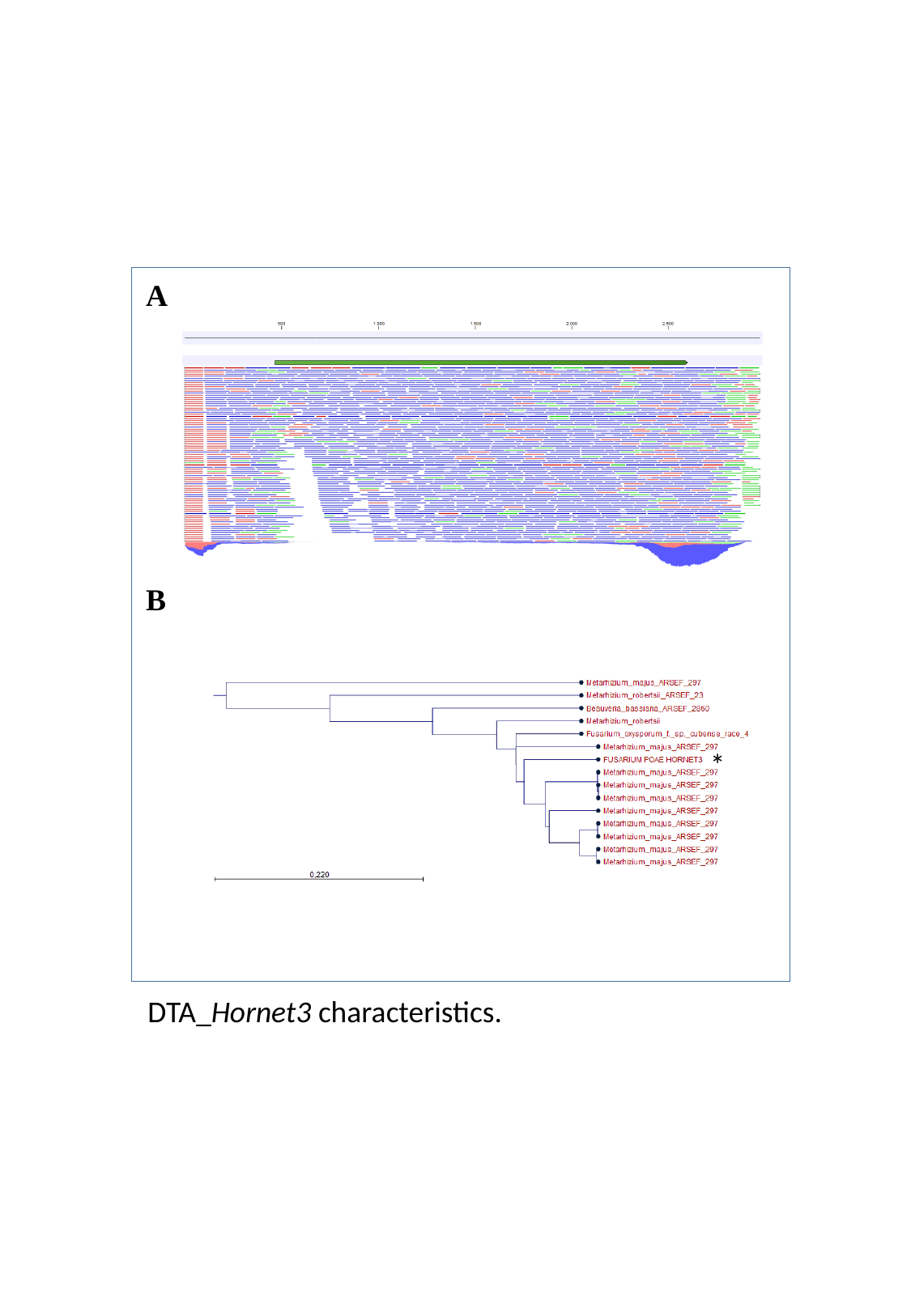

A
B
*
DTA_Hornet3 characteristics.

## Slide 28
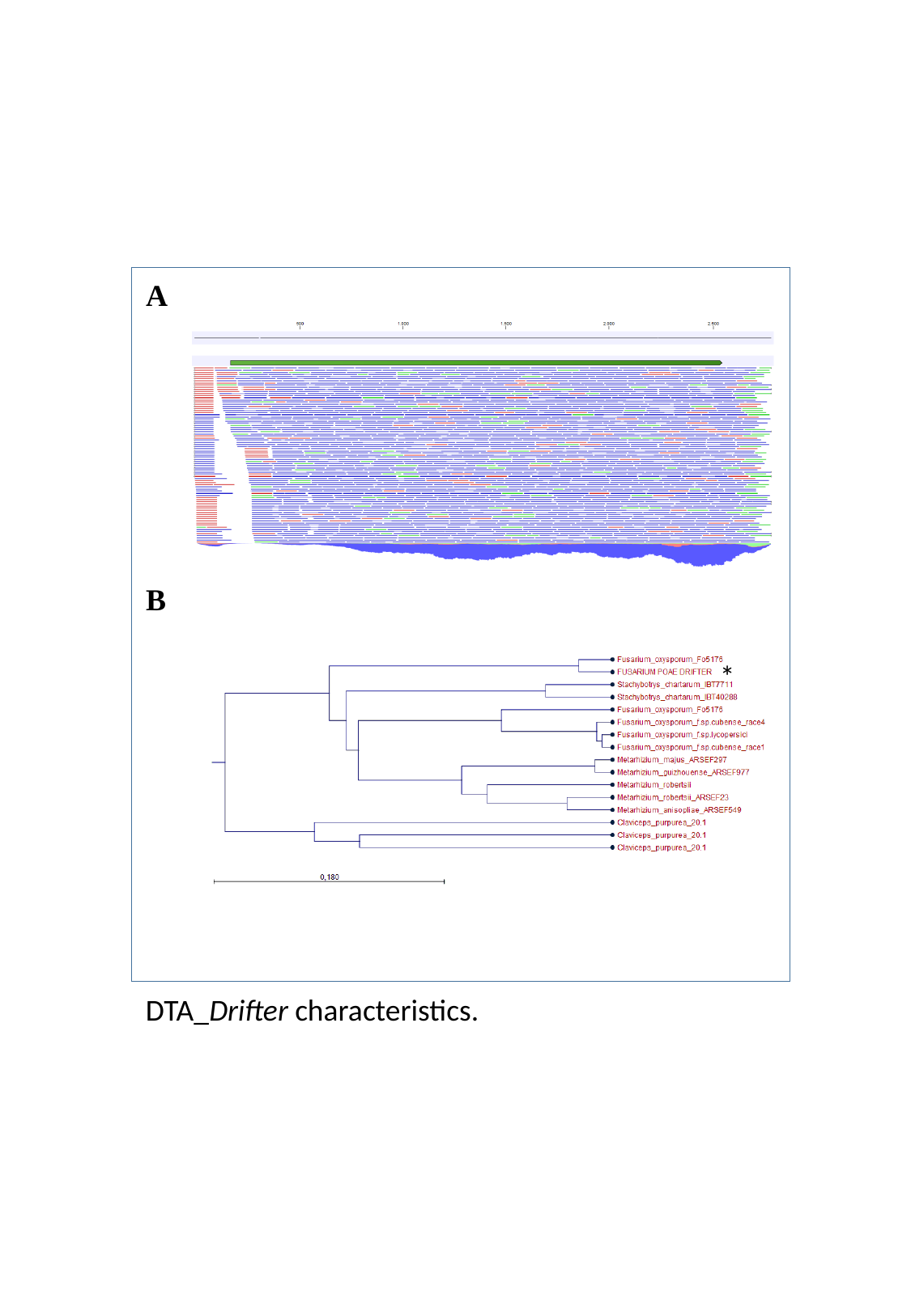

A
B
*
DTA_Drifter characteristics.

## Slide 29
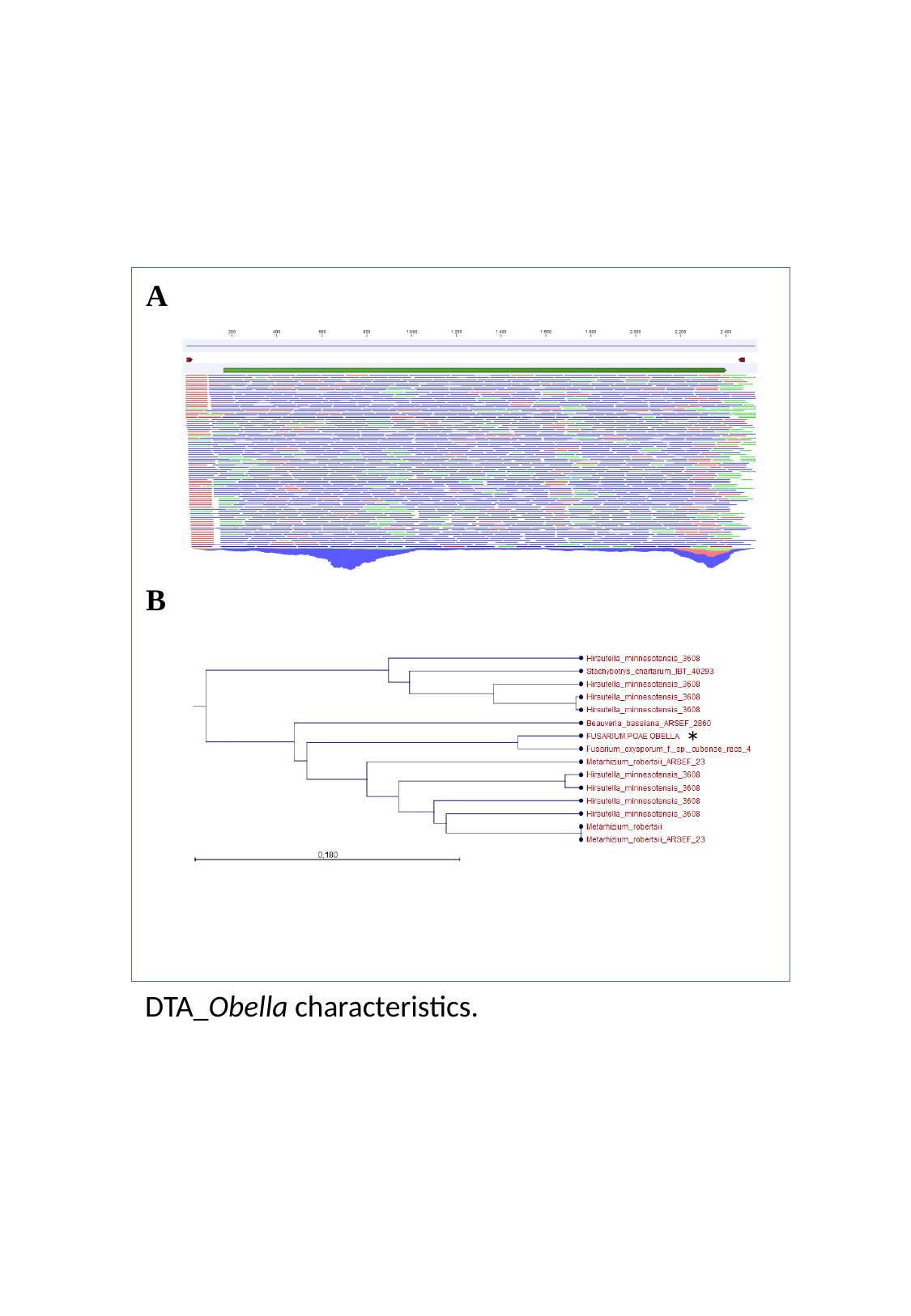

A
B
*
DTA_Obella characteristics.

## Slide 30
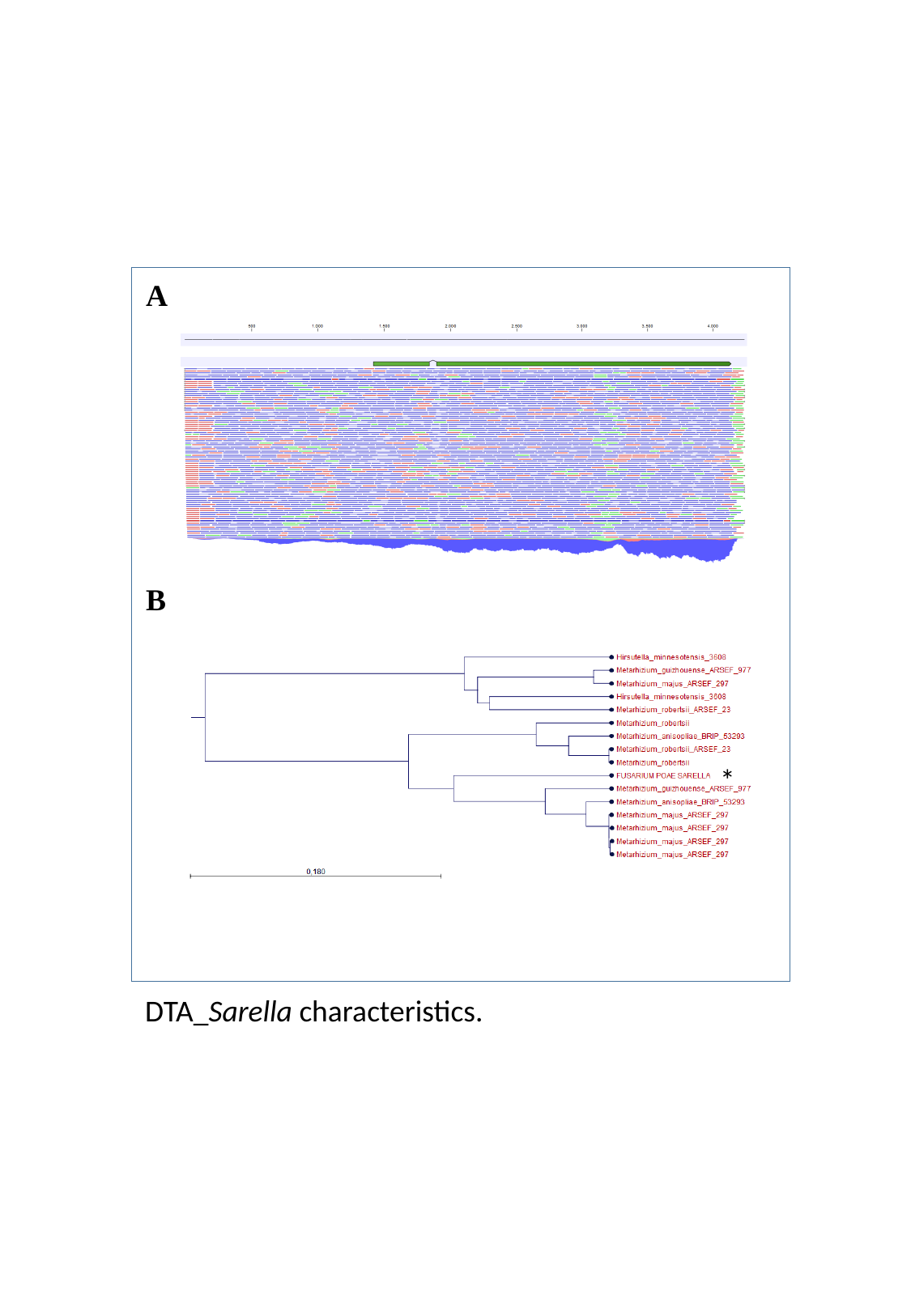

A
B
*
DTA_Sarella characteristics.

## Slide 31
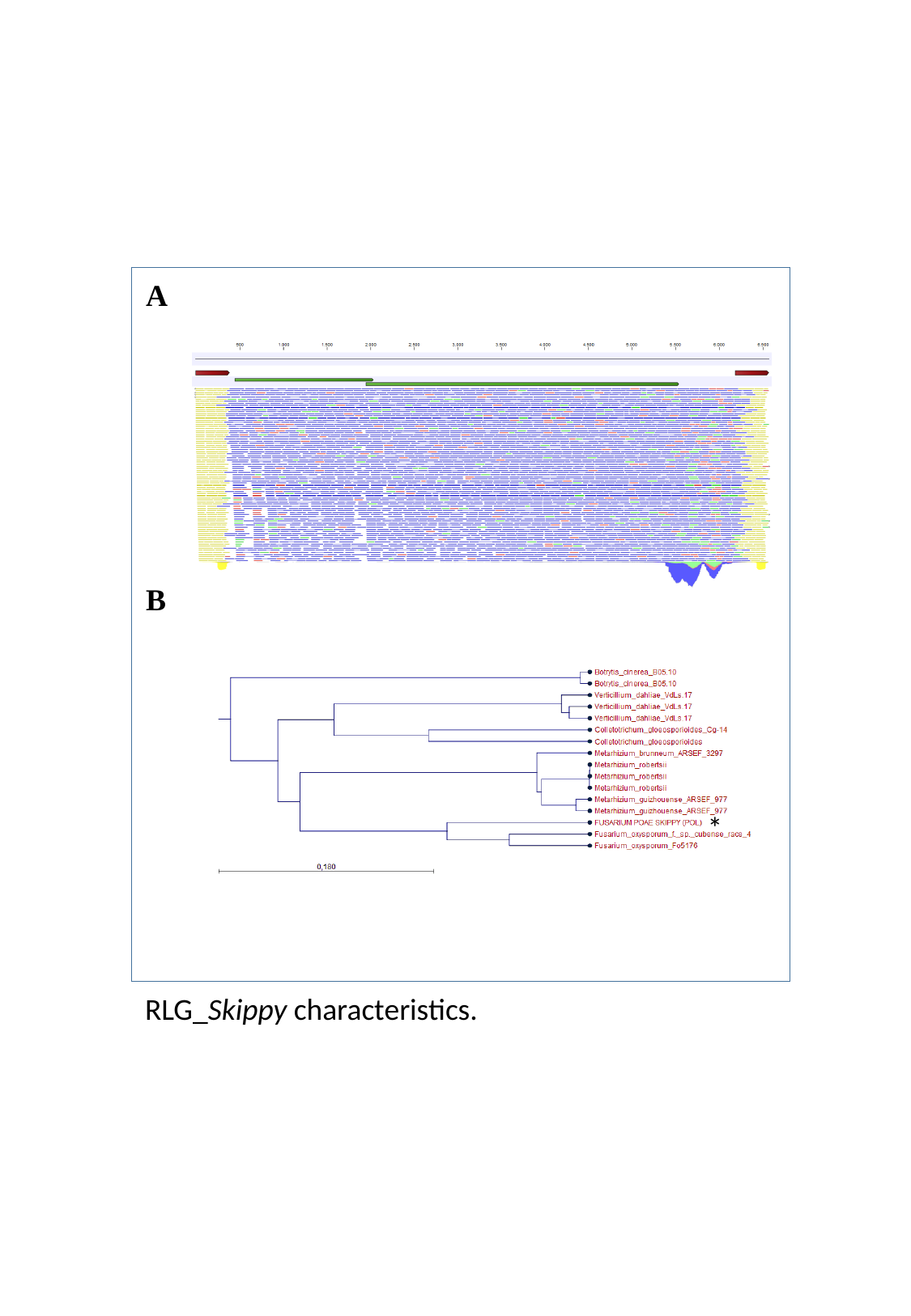

A
B
*
RLG_Skippy characteristics.

## Slide 32
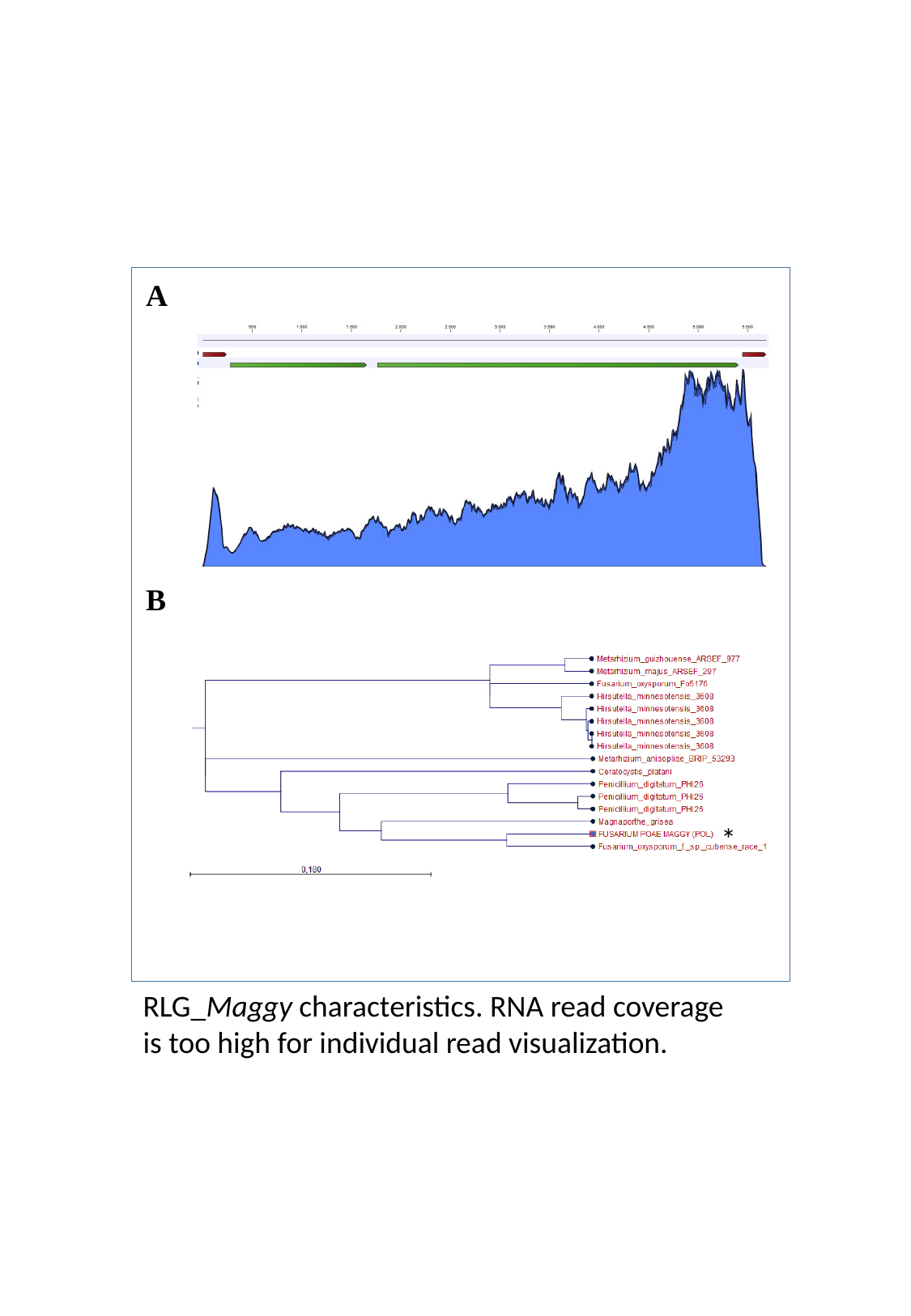

A
B
*
RLG_Maggy characteristics. RNA read coverage is too high for individual read visualization.

## Slide 33
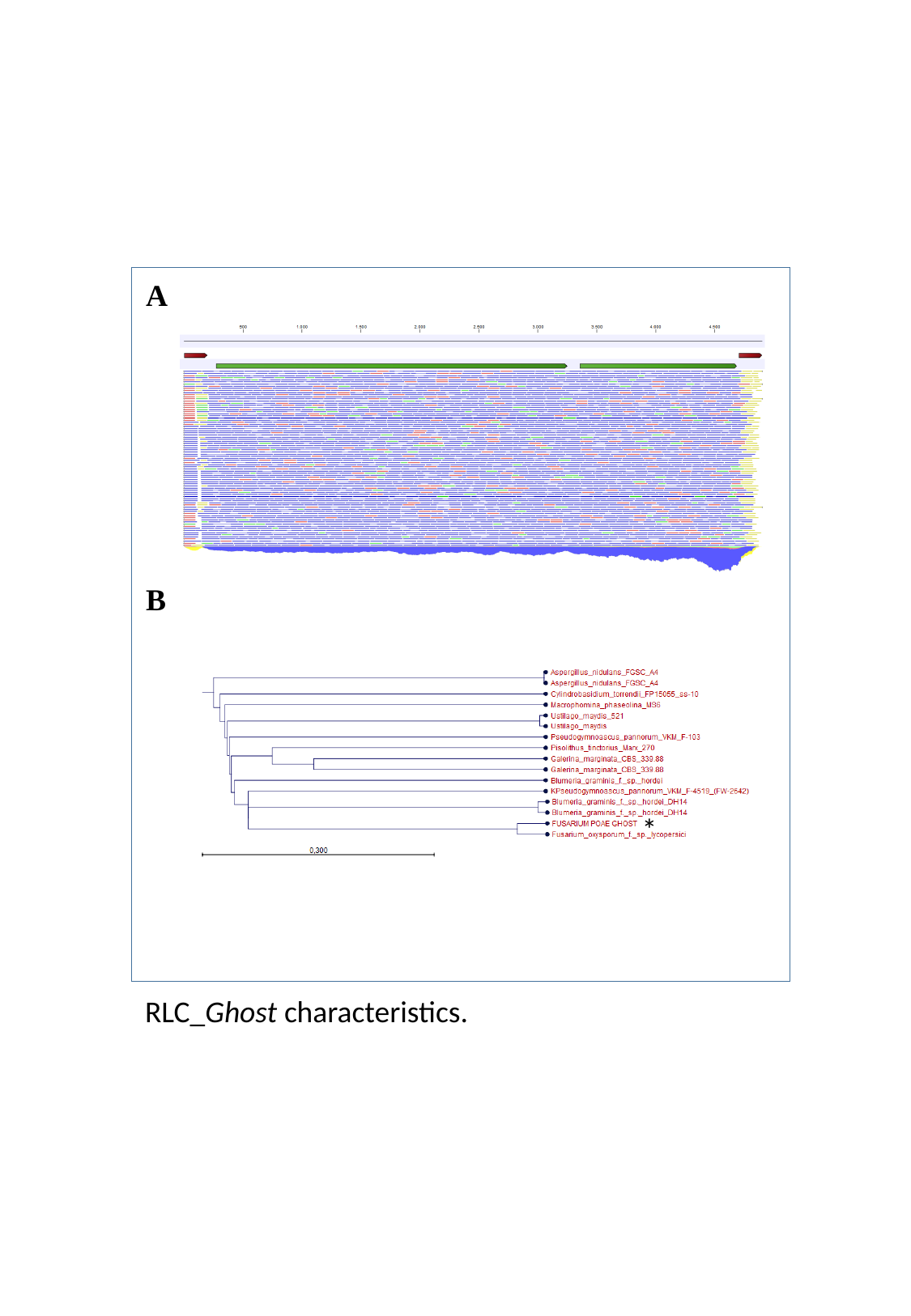

A
B
*
RLC_Ghost characteristics.

## Slide 34
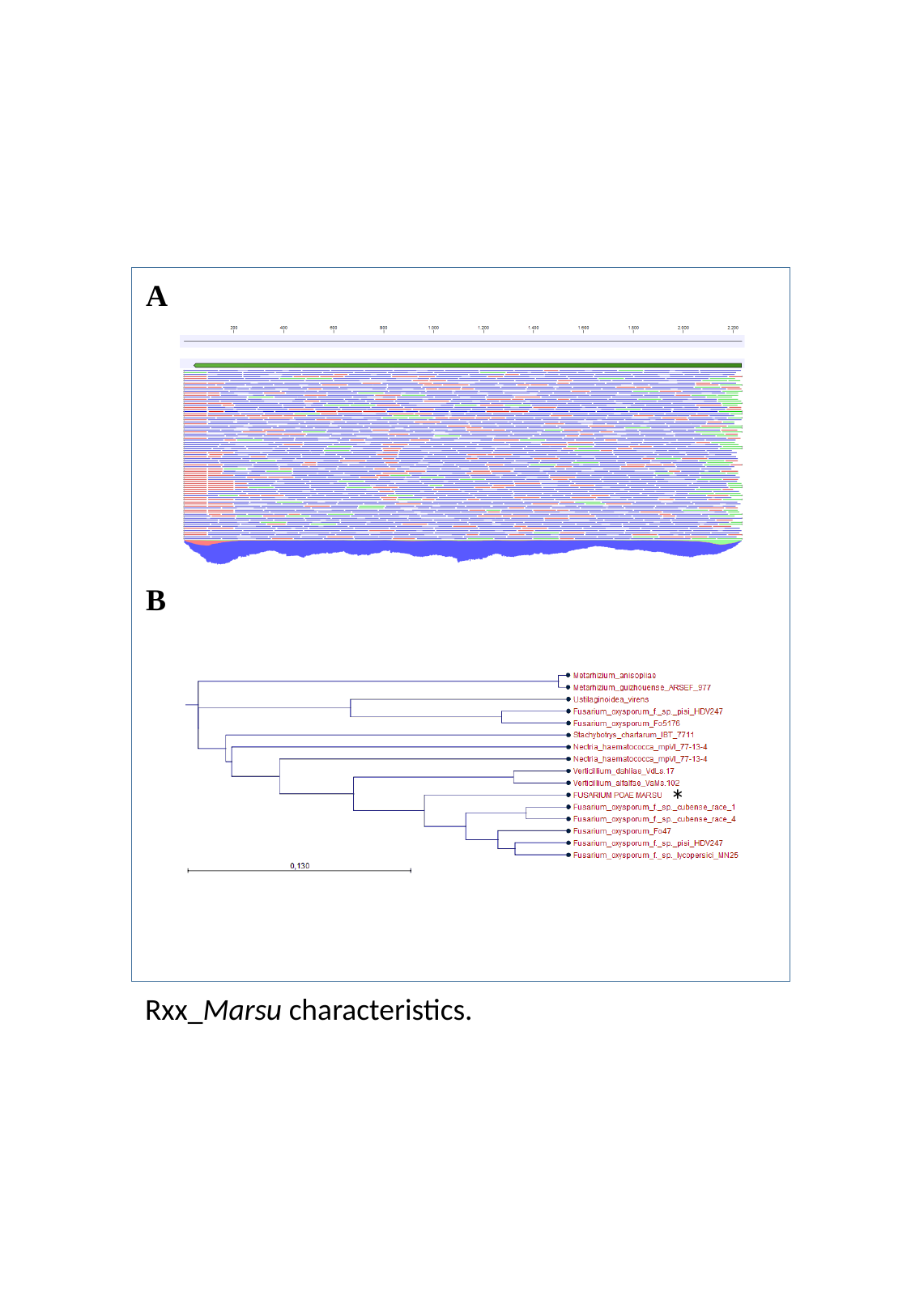

A
B
*
Rxx_Marsu characteristics.
