# Supplementary material for: Living apart together: crosstalk between the core and supernumerary genomes in a fungal plant pathogen
Source: BMC Genomics. 2016 Aug 23;17(1):670. doi: 10.1186/s12864-016-2941-6 (PMC4994206; doi:10.1186/s12864-016-2941-6)
Supplement: Additional file 7: — RIPcal analysis of transposable element families on the core and the supernumerary genome. A comprehensive RIPcal analysis was performed on the core and the supernumerary genome separately. Based on an unbiased approach, 13 families out of 33 show patterns typical for RIP, and this is only detected on the core genome. (PPTX 735 kb) [file 12864_2016_2941_MOESM7_ESM.pptx]

## Slide 1
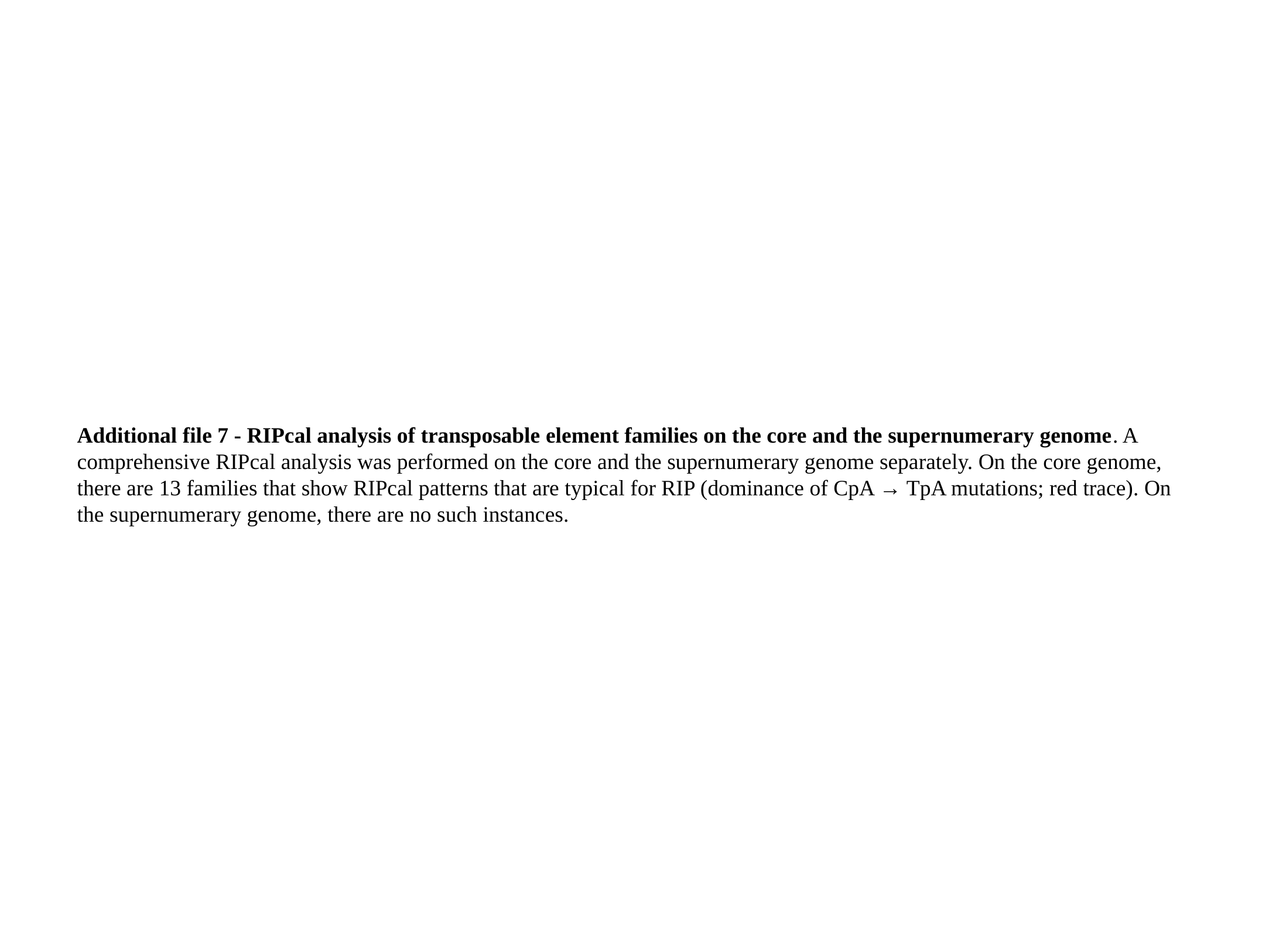

Additional file 7 - RIPcal analysis of transposable element families on the core and the supernumerary genome. A comprehensive RIPcal analysis was performed on the core and the supernumerary genome separately. On the core genome, there are 13 families that show RIPcal patterns that are typical for RIP (dominance of CpA → TpA mutations; red trace). On the supernumerary genome, there are no such instances.

## Slide 2
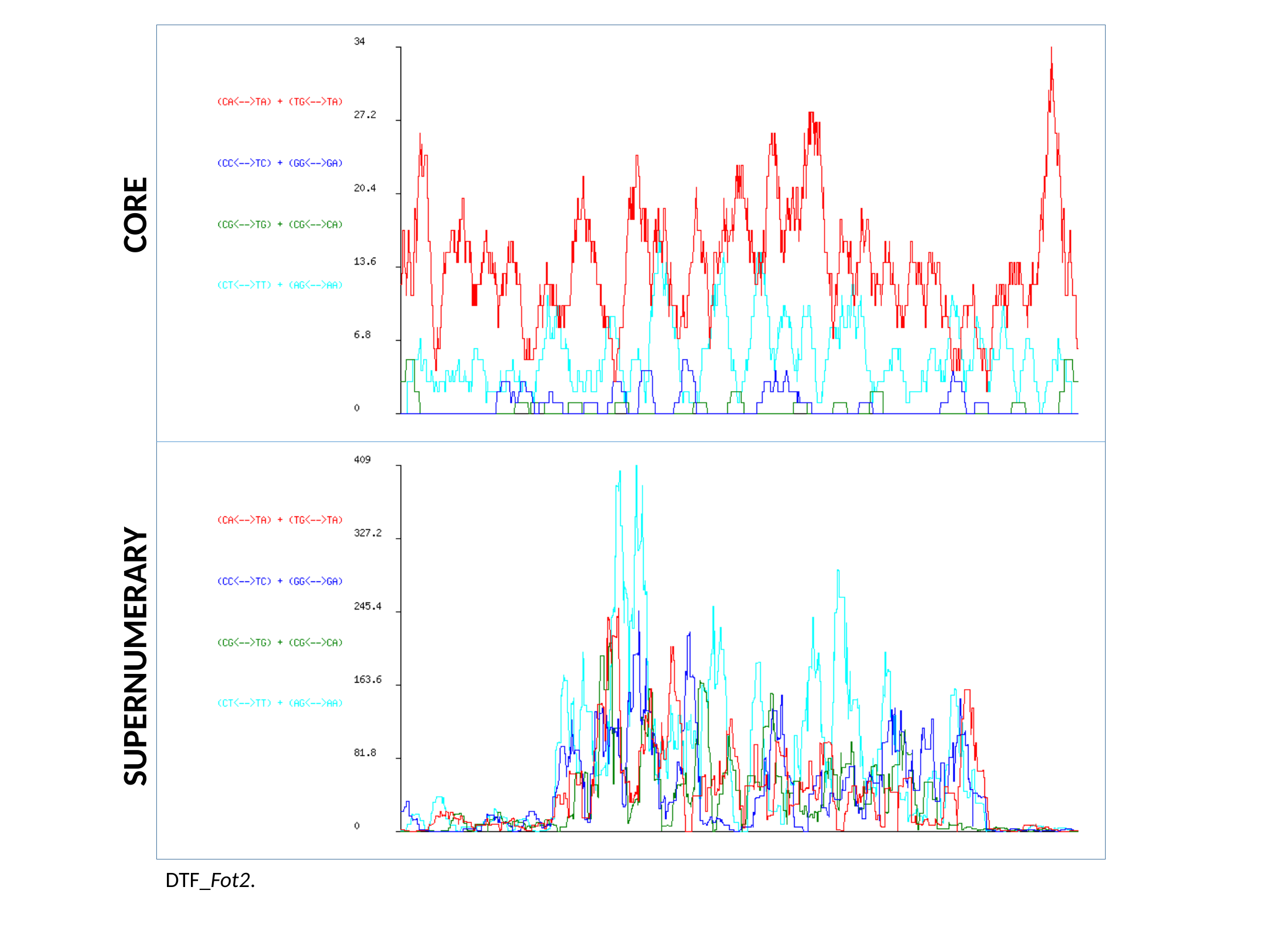

CORE
SUPERNUMERARY
DTF_Fot2.

## Slide 3
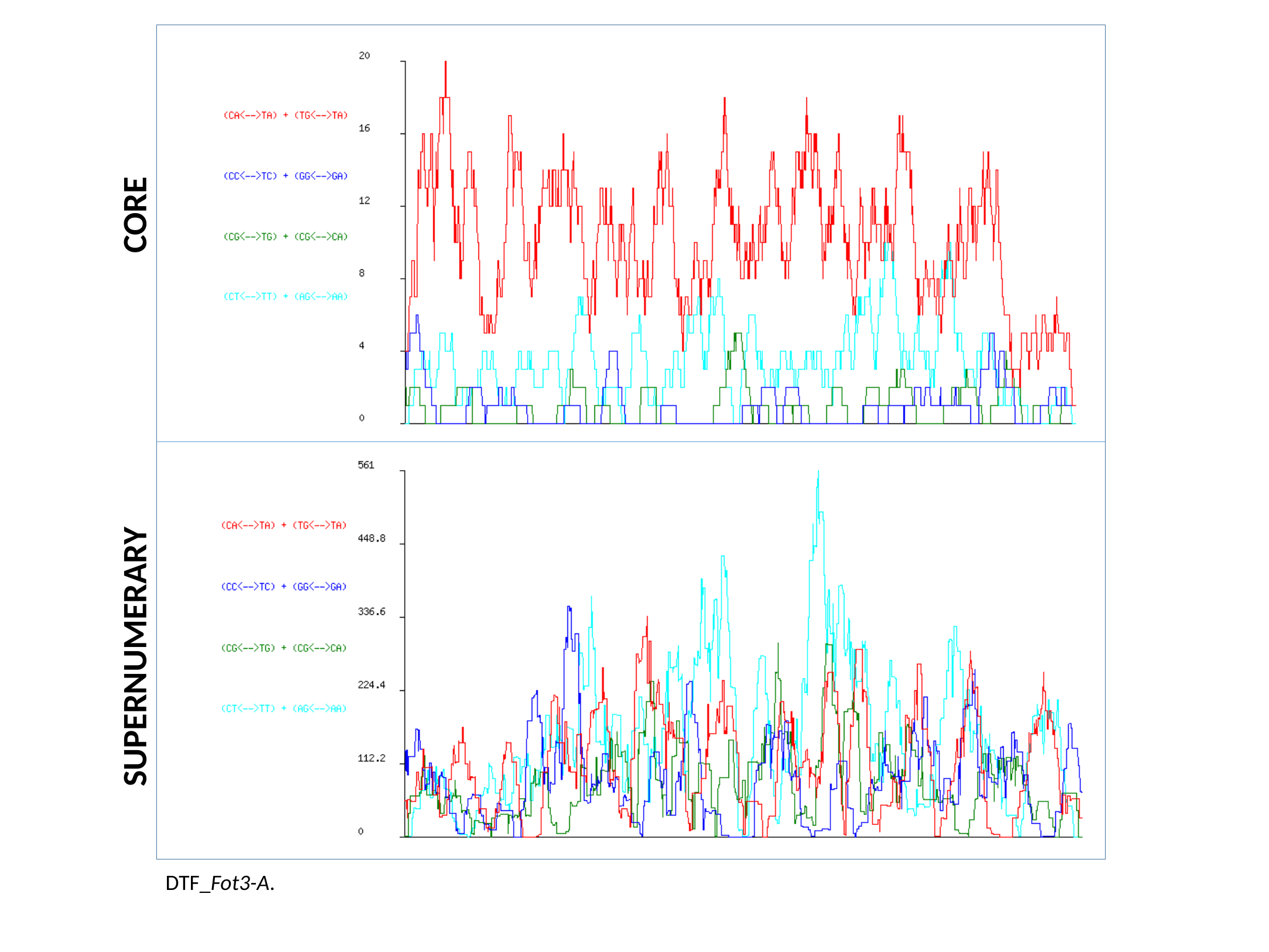

CORE
SUPERNUMERARY
DTF_Fot3-A.

## Slide 4
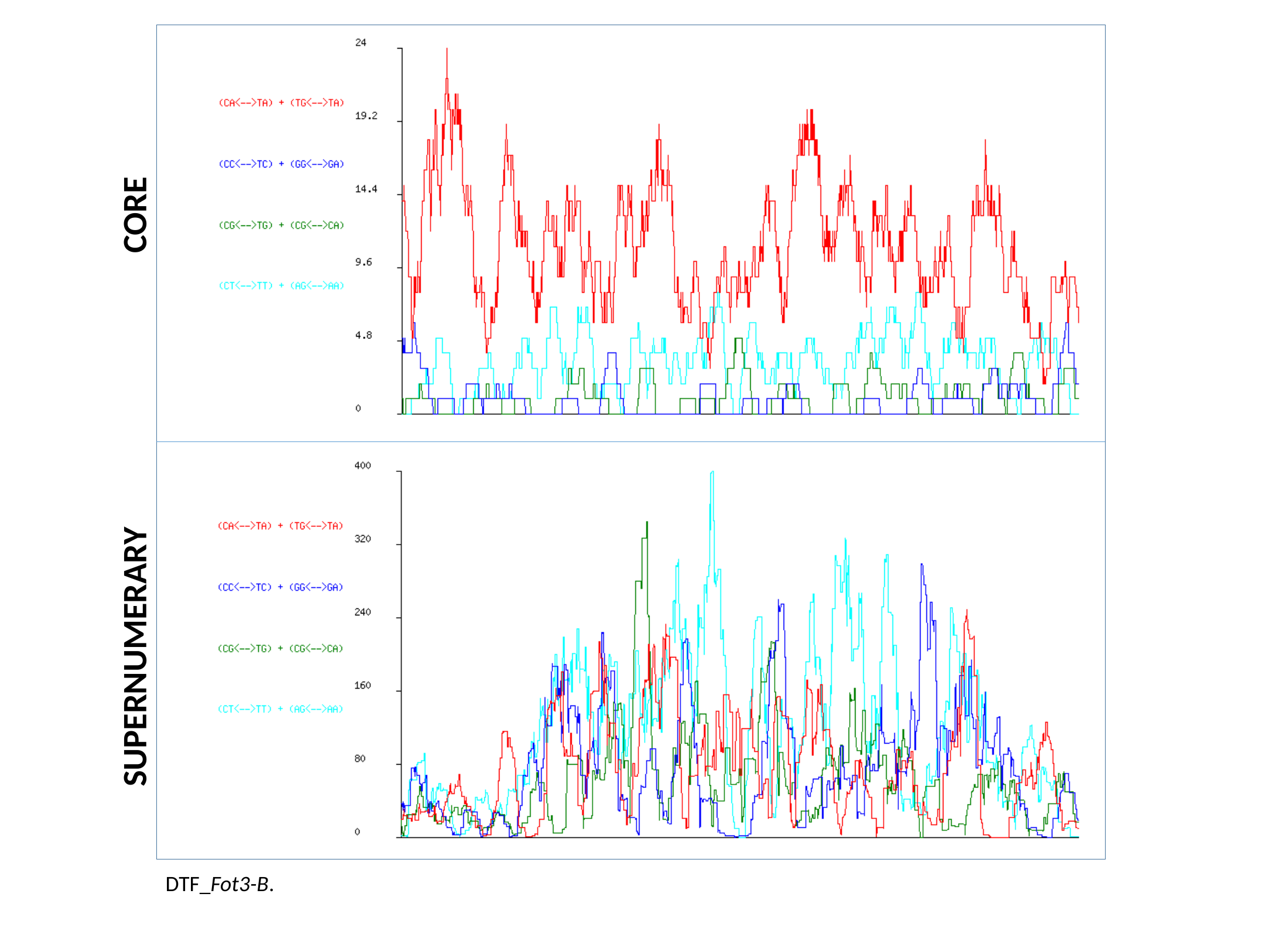

CORE
SUPERNUMERARY
DTF_Fot3-B.

## Slide 5
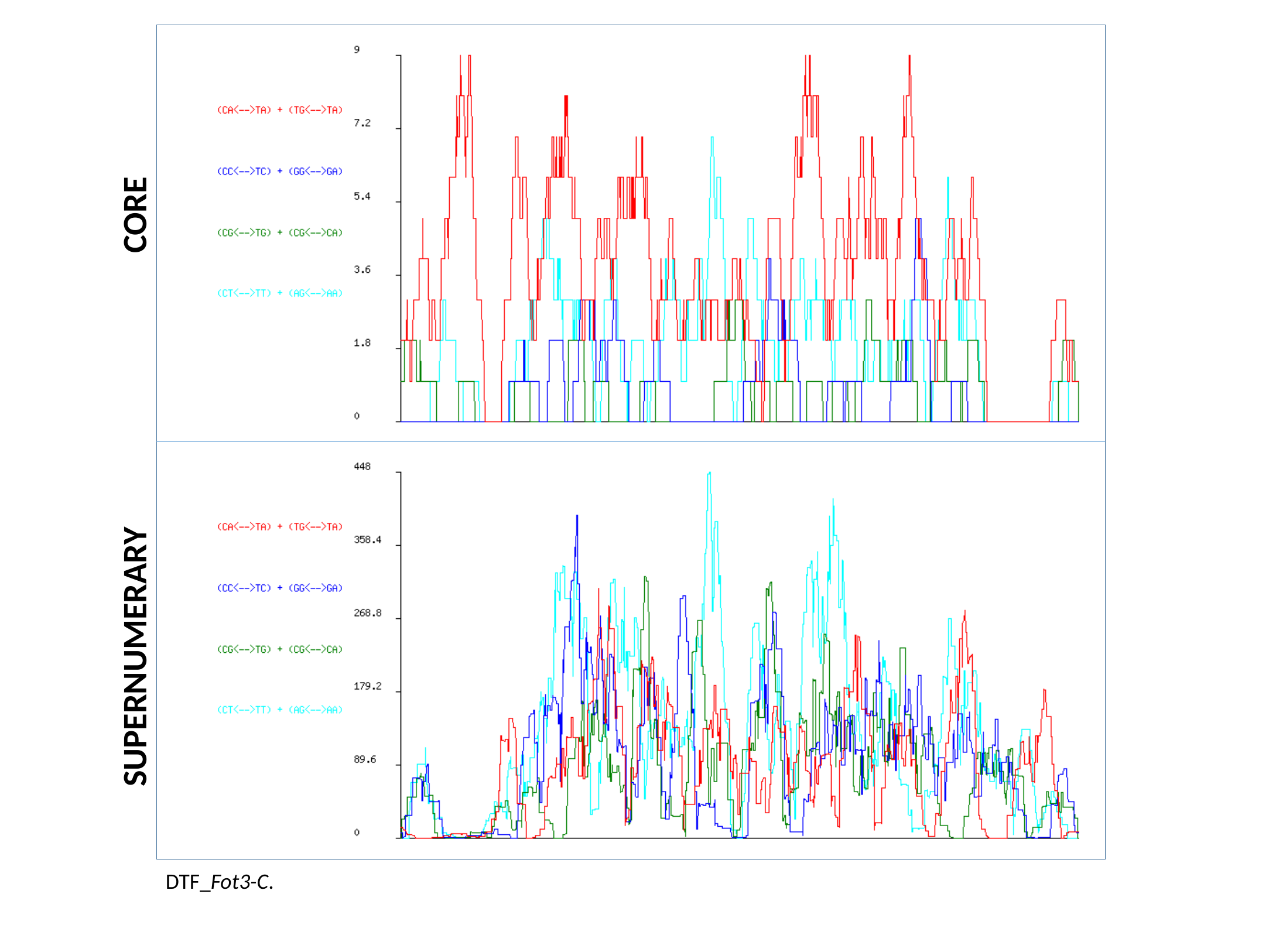

CORE
SUPERNUMERARY
DTF_Fot3-C.

## Slide 6
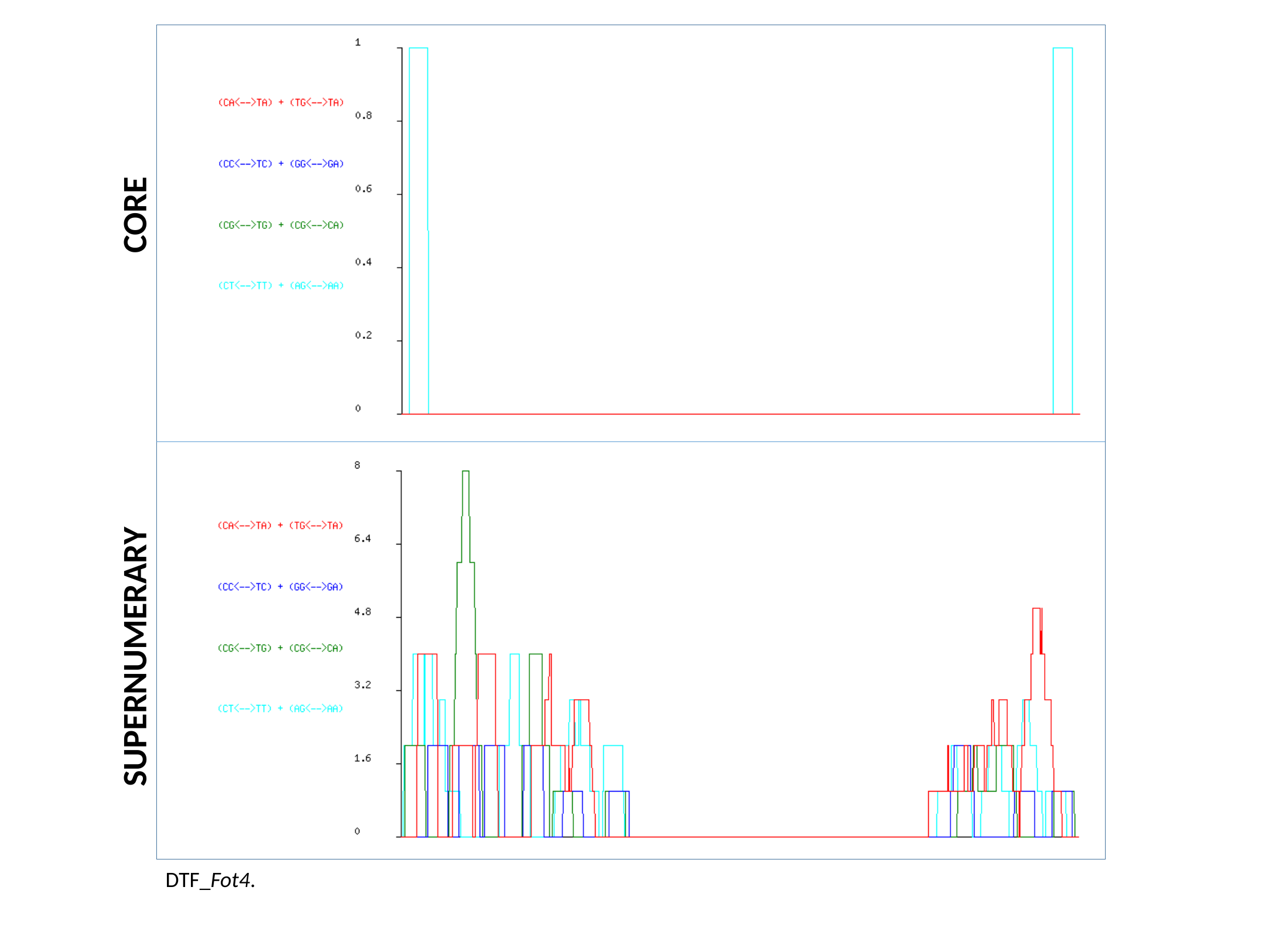

CORE
SUPERNUMERARY
DTF_Fot4.

## Slide 7
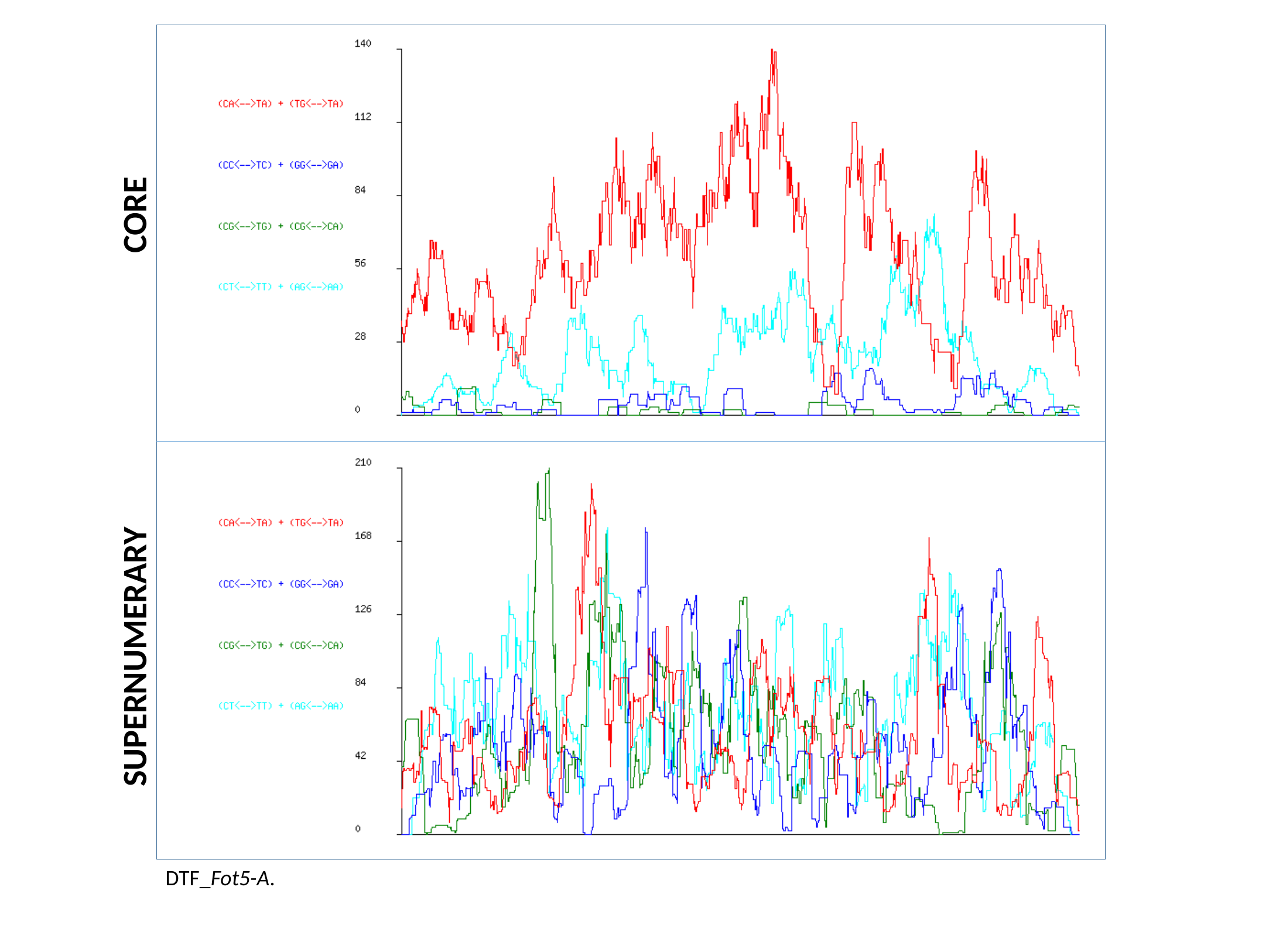

CORE
SUPERNUMERARY
DTF_Fot5-A.

## Slide 8
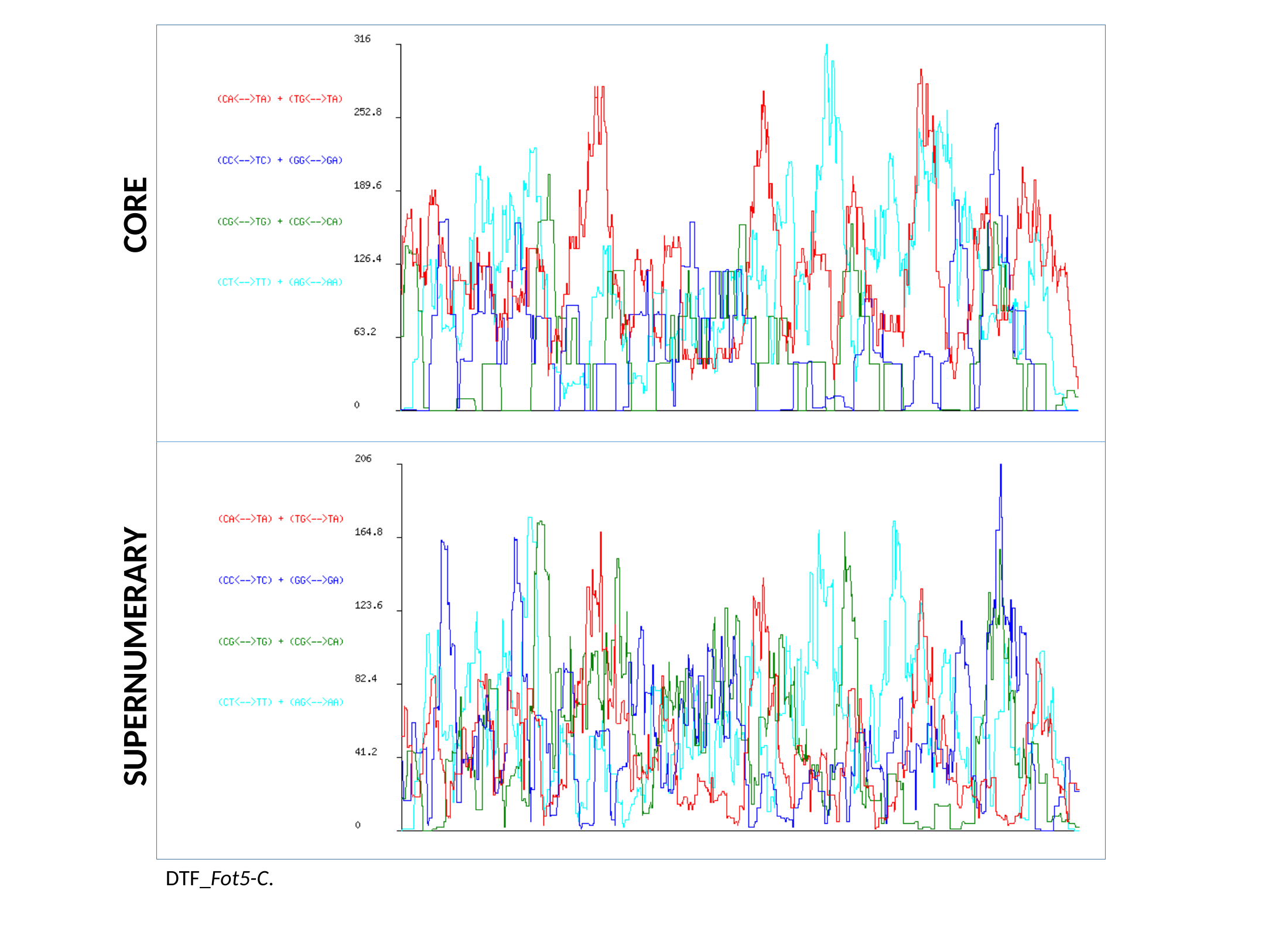

CORE
SUPERNUMERARY
DTF_Fot5-C.

## Slide 9
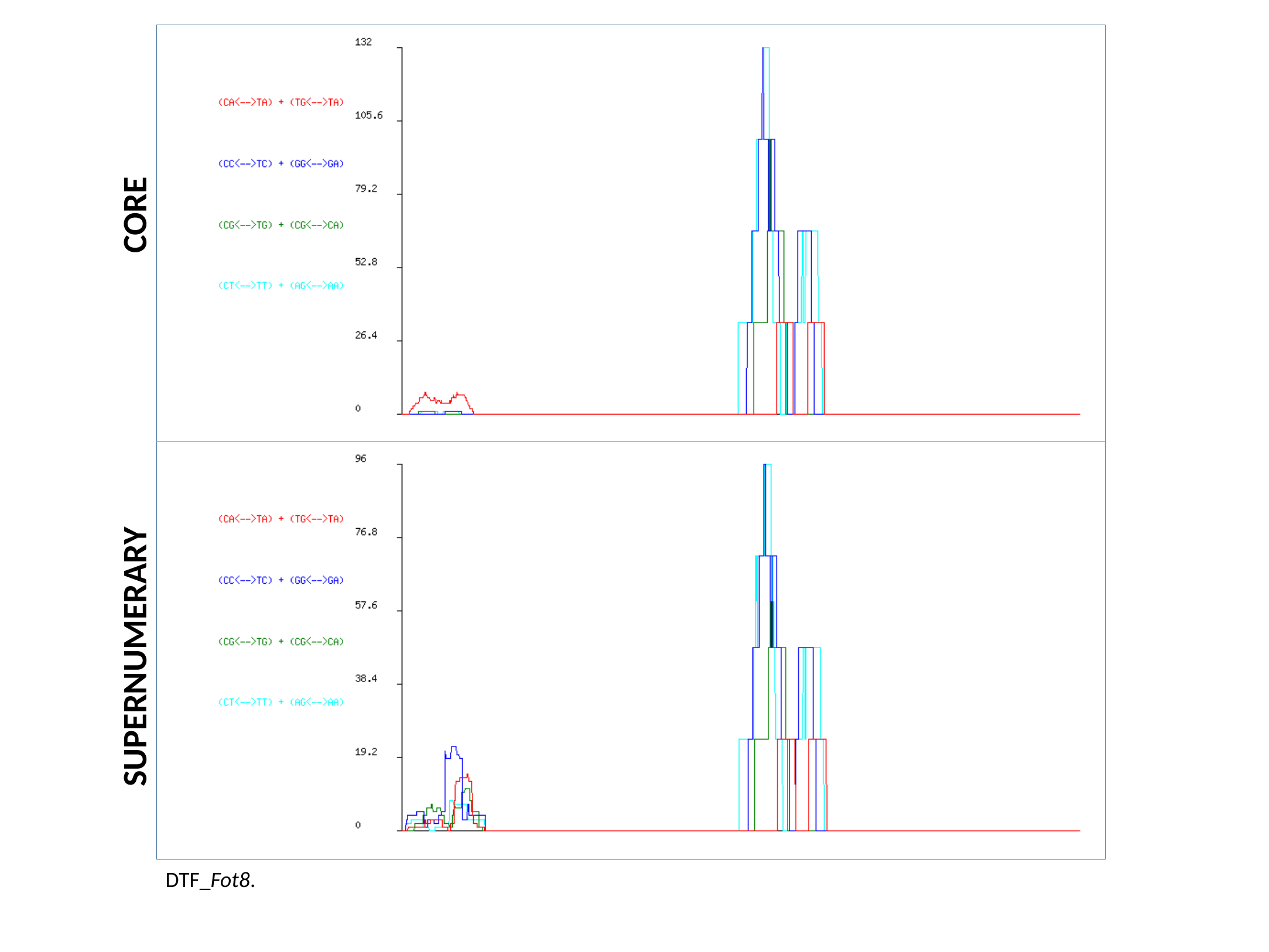

CORE
SUPERNUMERARY
DTF_Fot8.

## Slide 10
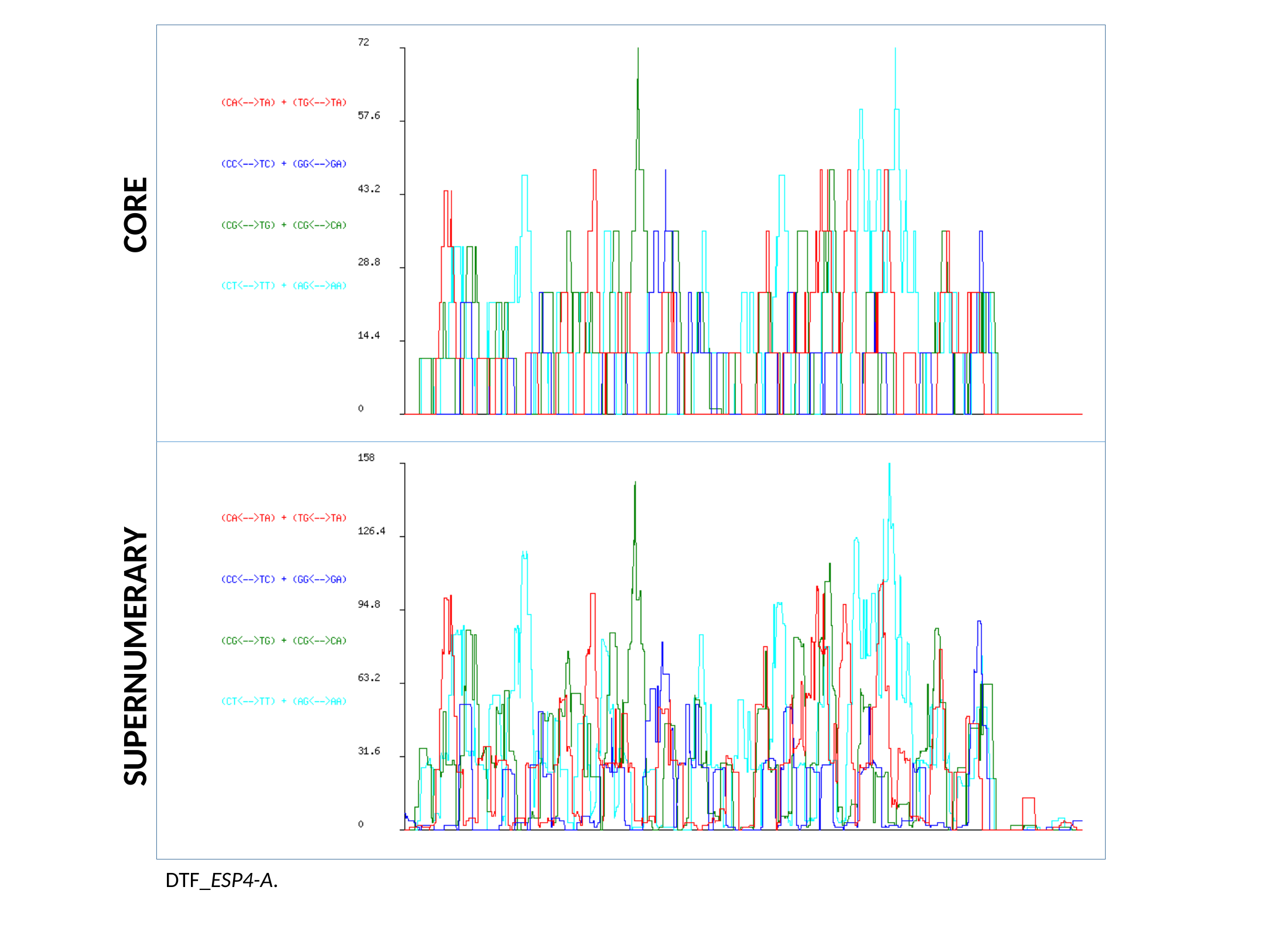

CORE
SUPERNUMERARY
DTF_ESP4-A.

## Slide 11
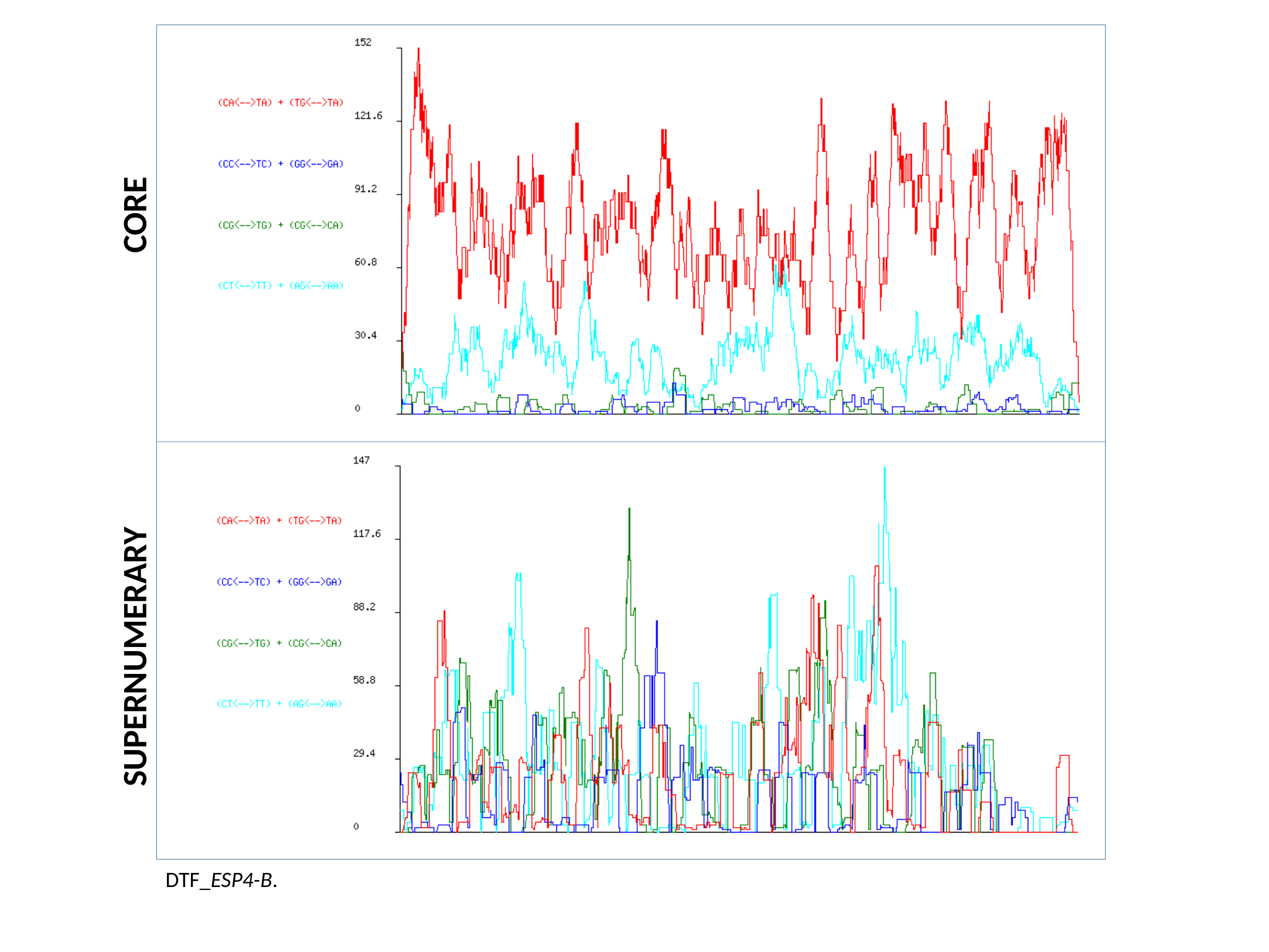

CORE
SUPERNUMERARY
DTF_ESP4-B.

## Slide 12
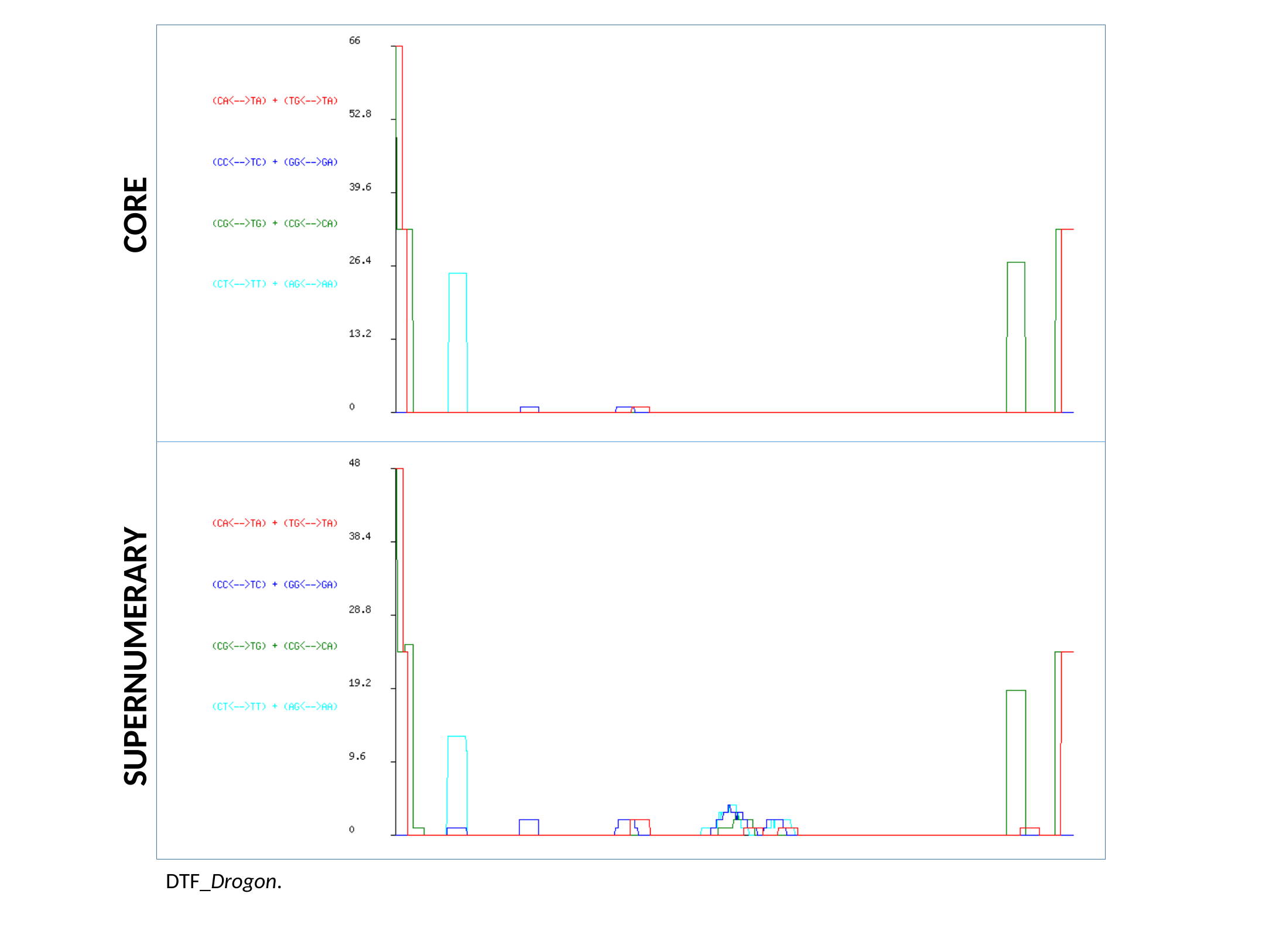

CORE
SUPERNUMERARY
DTF_Drogon.

## Slide 13
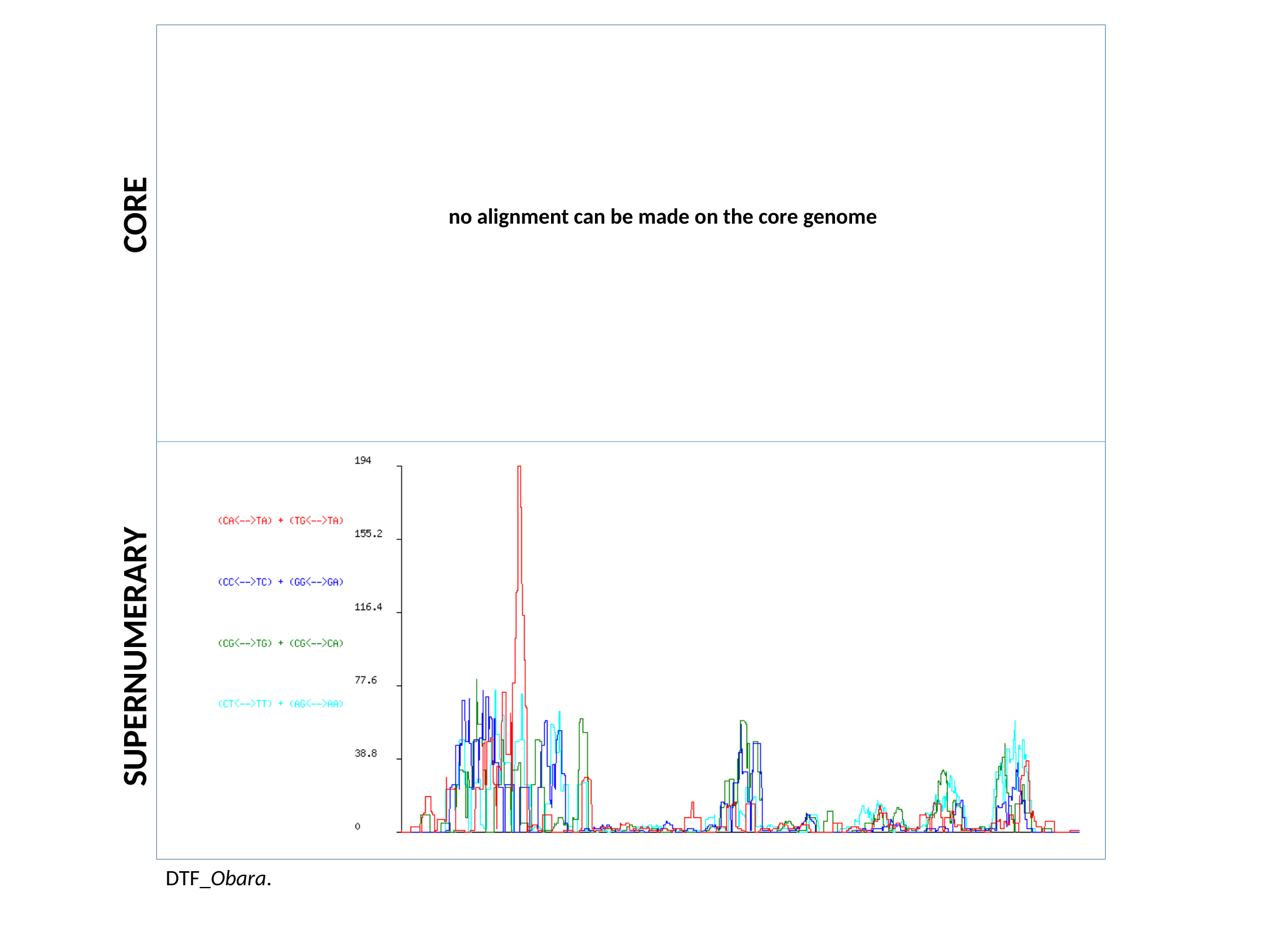

CORE
no alignment can be made on the core genome
SUPERNUMERARY
DTF_Obara.

## Slide 14
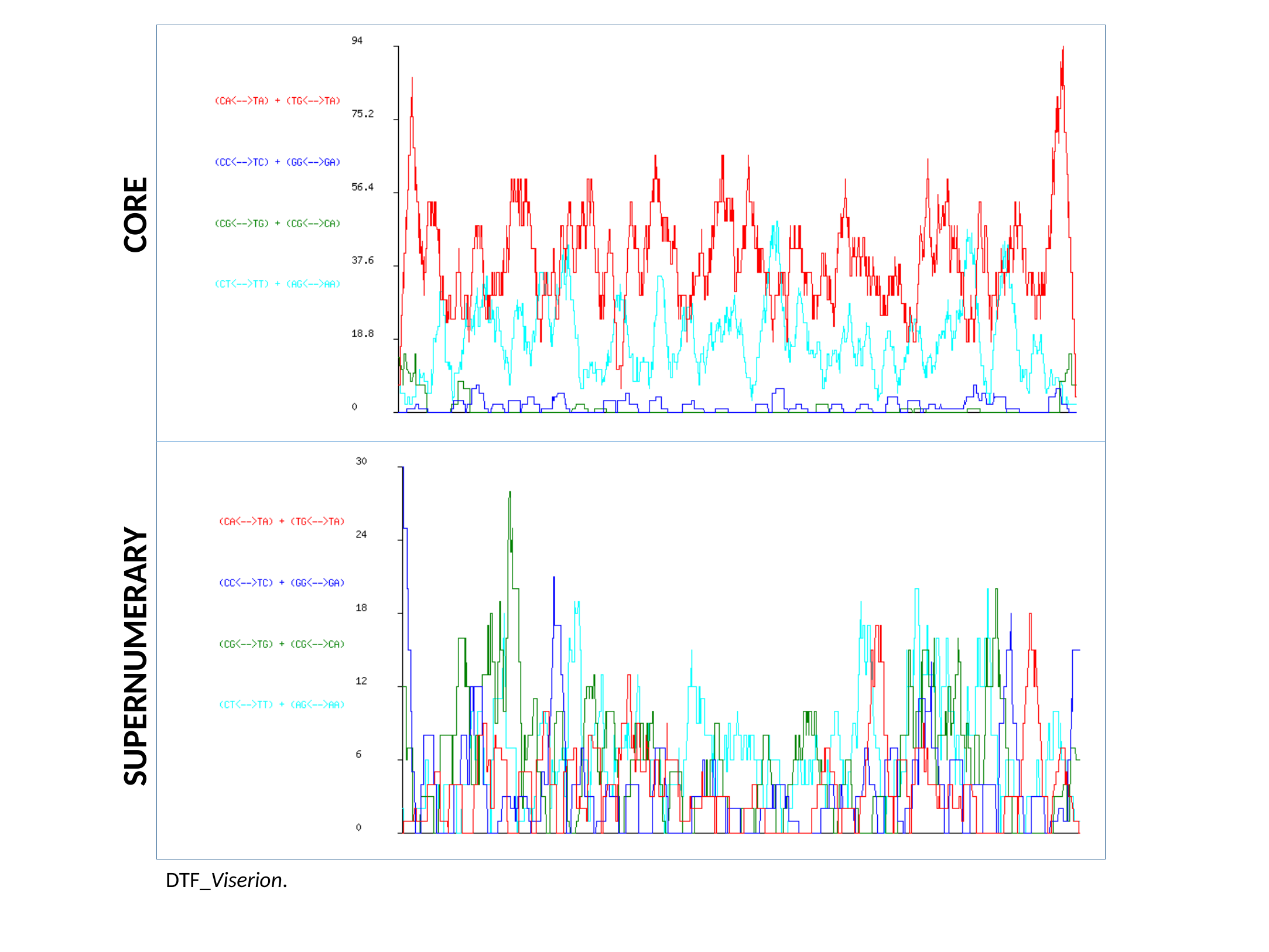

CORE
SUPERNUMERARY
DTF_Viserion.

## Slide 15
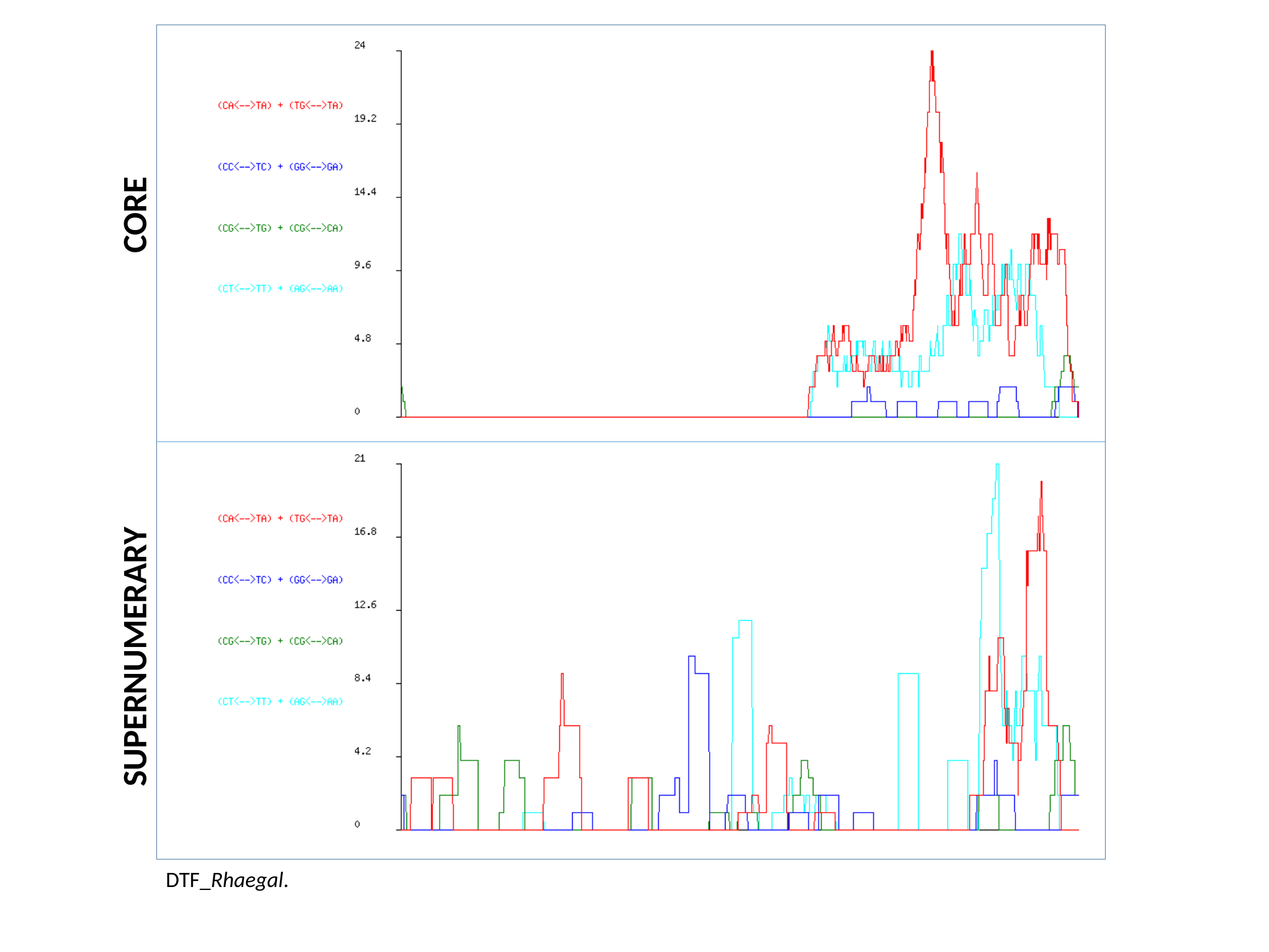

CORE
SUPERNUMERARY
DTF_Rhaegal.

## Slide 16
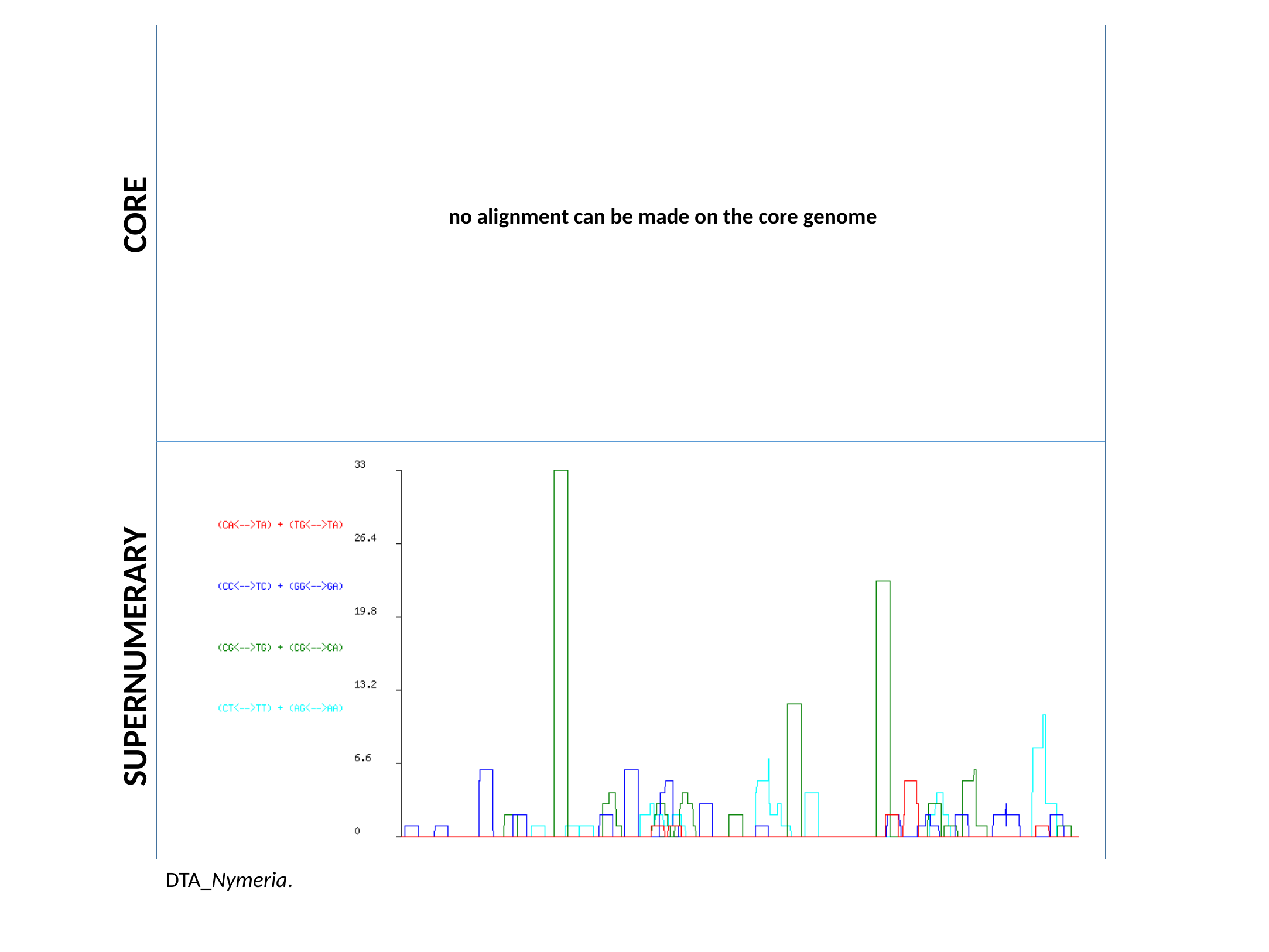

CORE
no alignment can be made on the core genome
SUPERNUMERARY
DTA_Nymeria.

## Slide 17
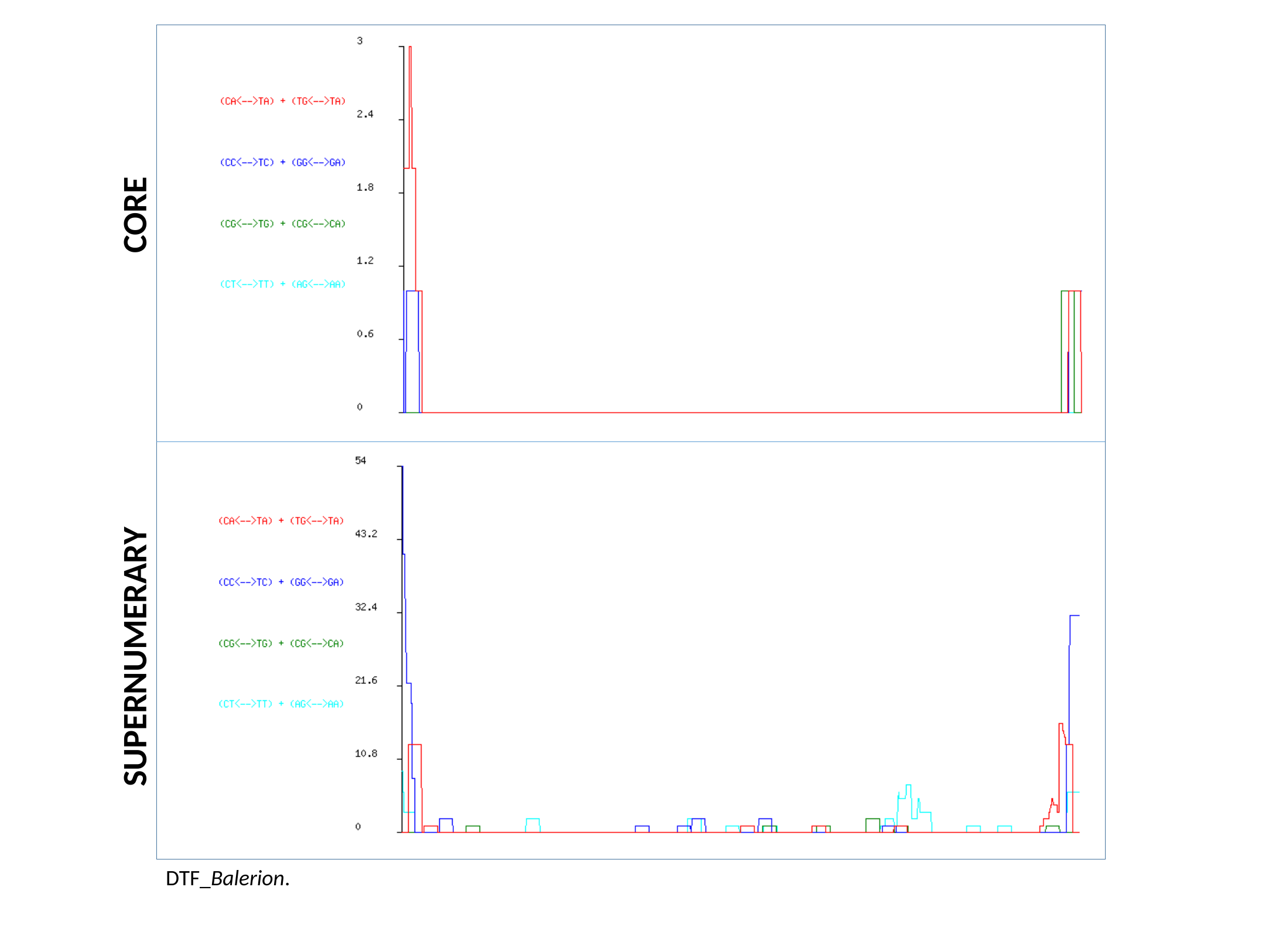

CORE
SUPERNUMERARY
DTF_Balerion.

## Slide 18
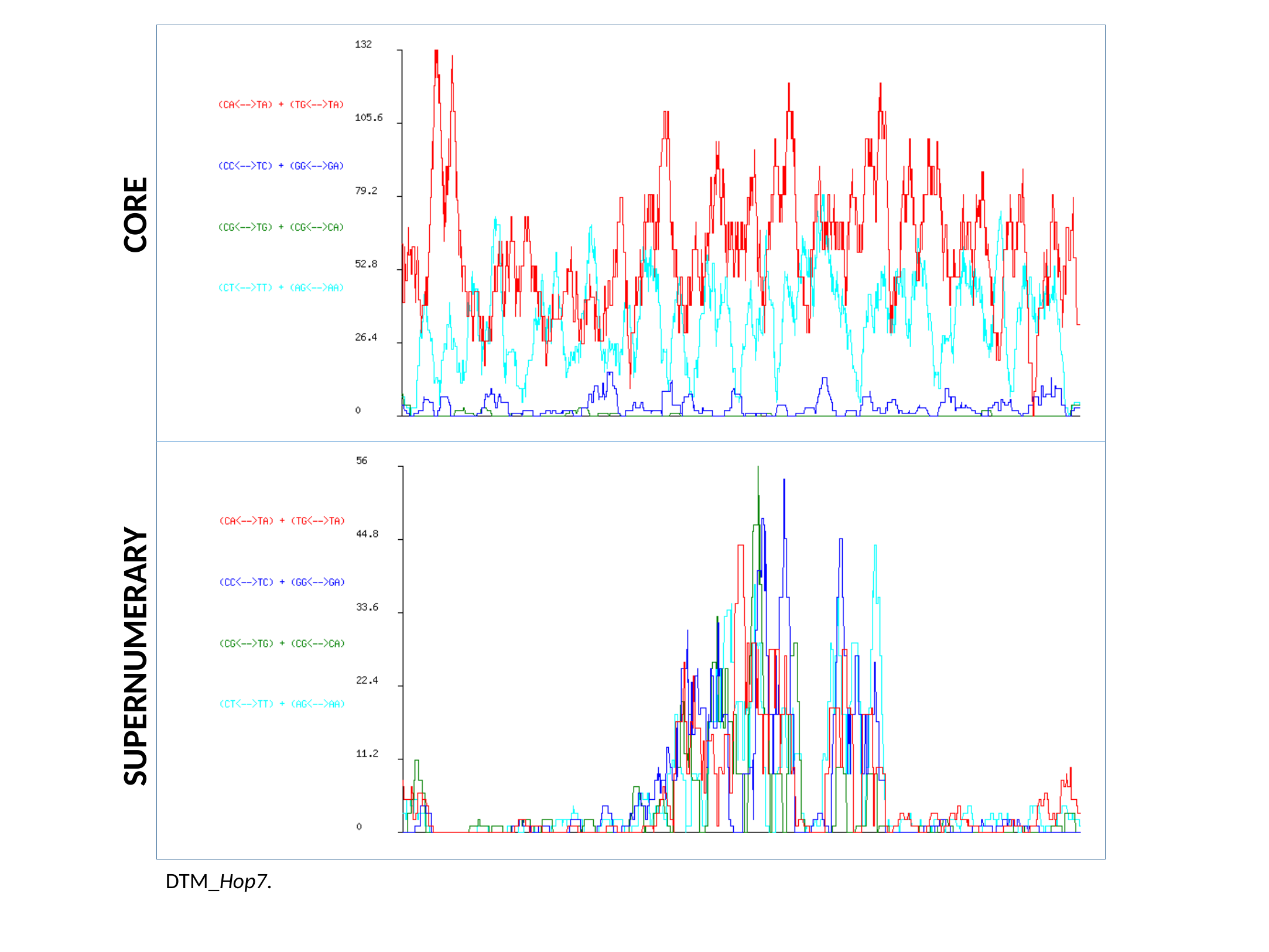

CORE
SUPERNUMERARY
DTM_Hop7.

## Slide 19
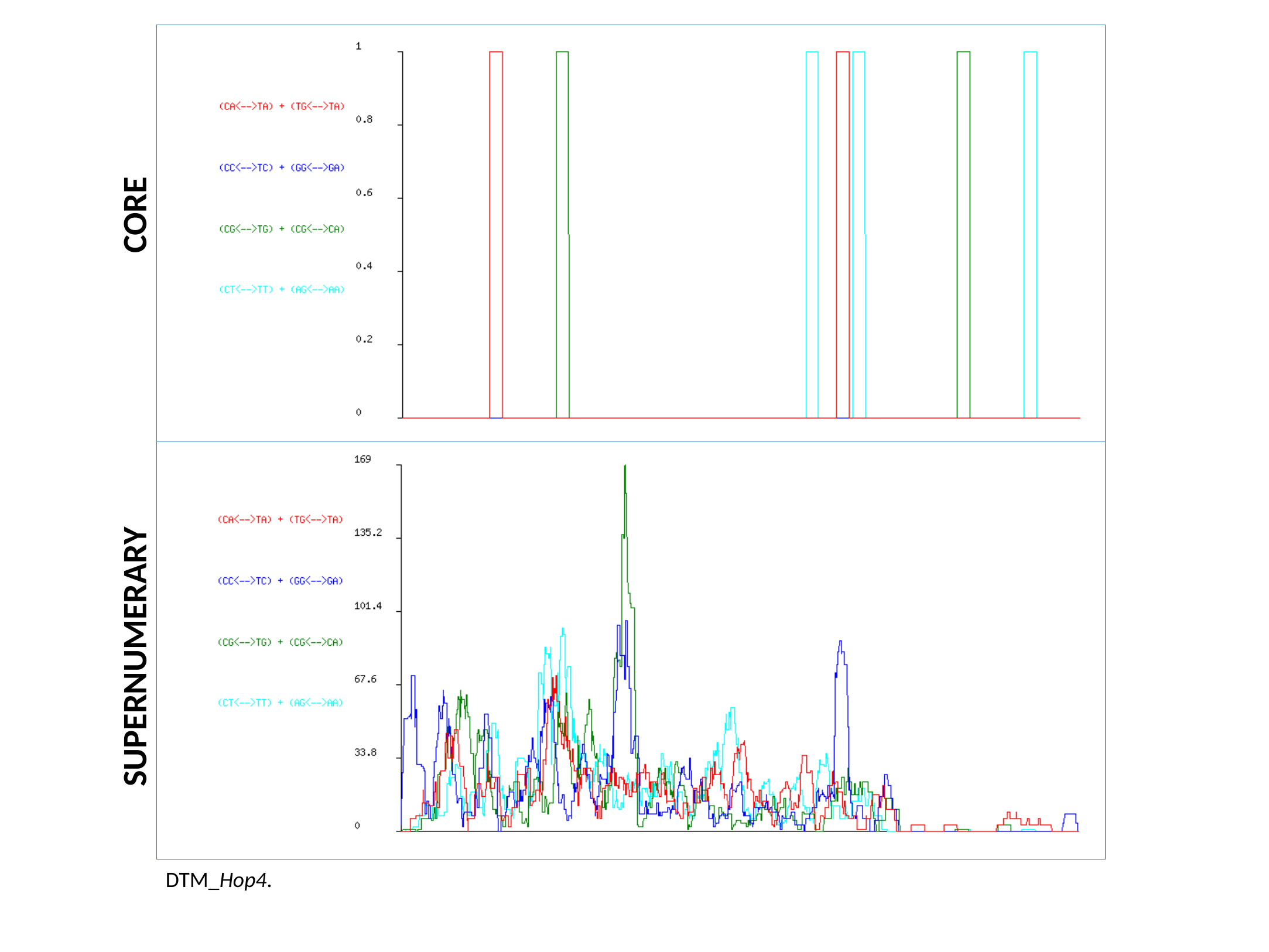

CORE
SUPERNUMERARY
DTM_Hop4.

## Slide 20
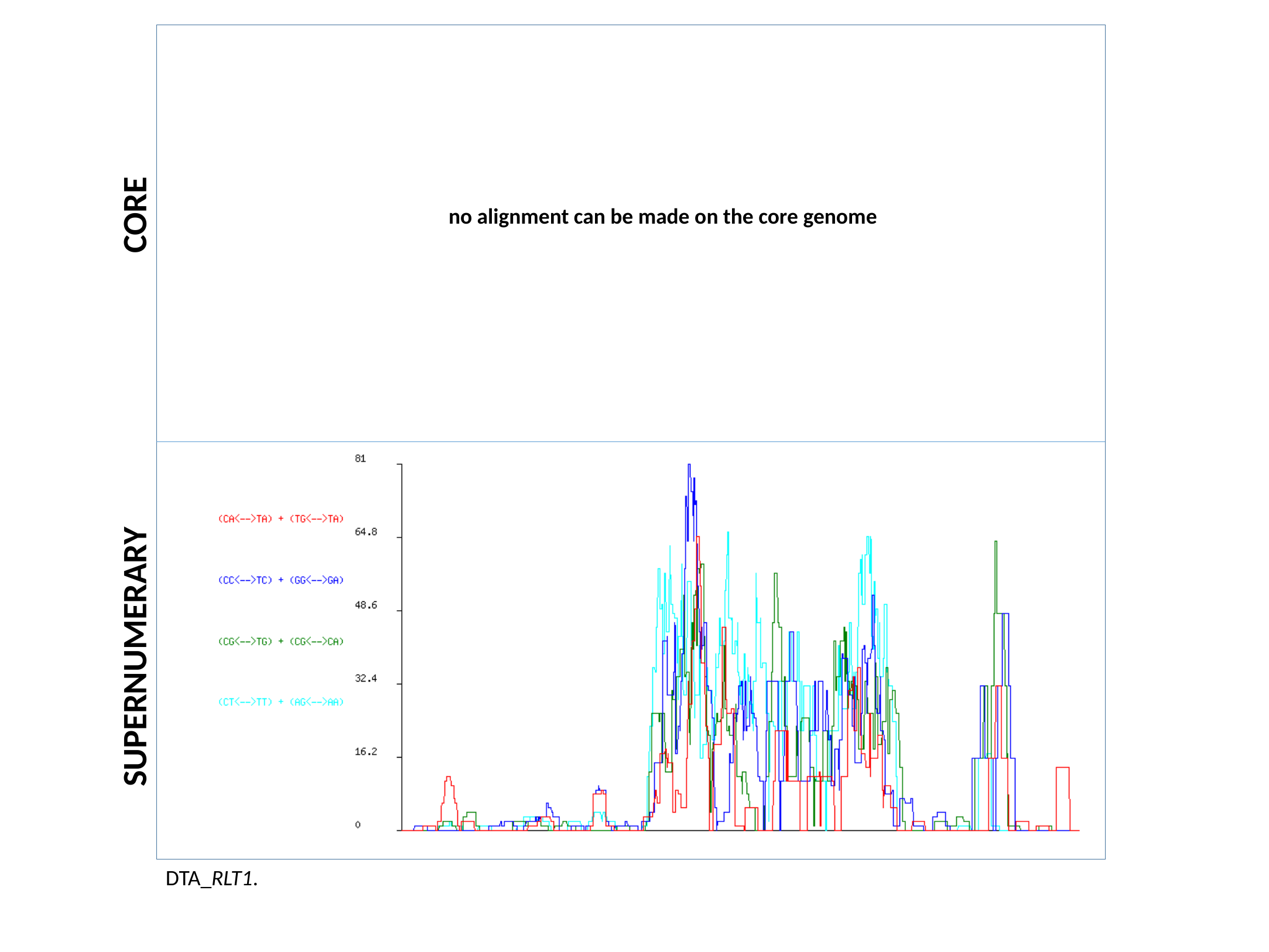

CORE
no alignment can be made on the core genome
SUPERNUMERARY
DTA_RLT1.

## Slide 21
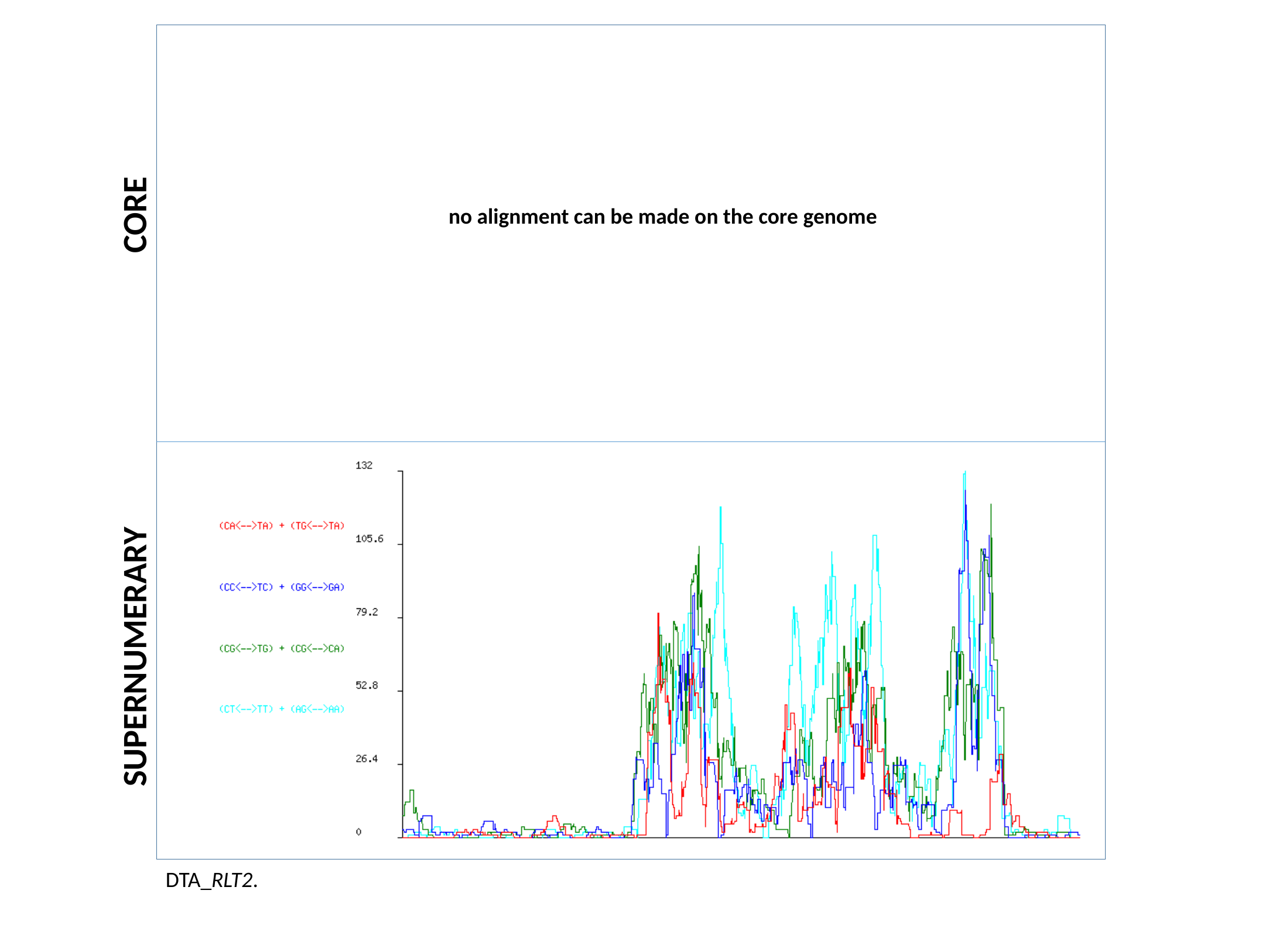

CORE
no alignment can be made on the core genome
SUPERNUMERARY
DTA_RLT2.

## Slide 22
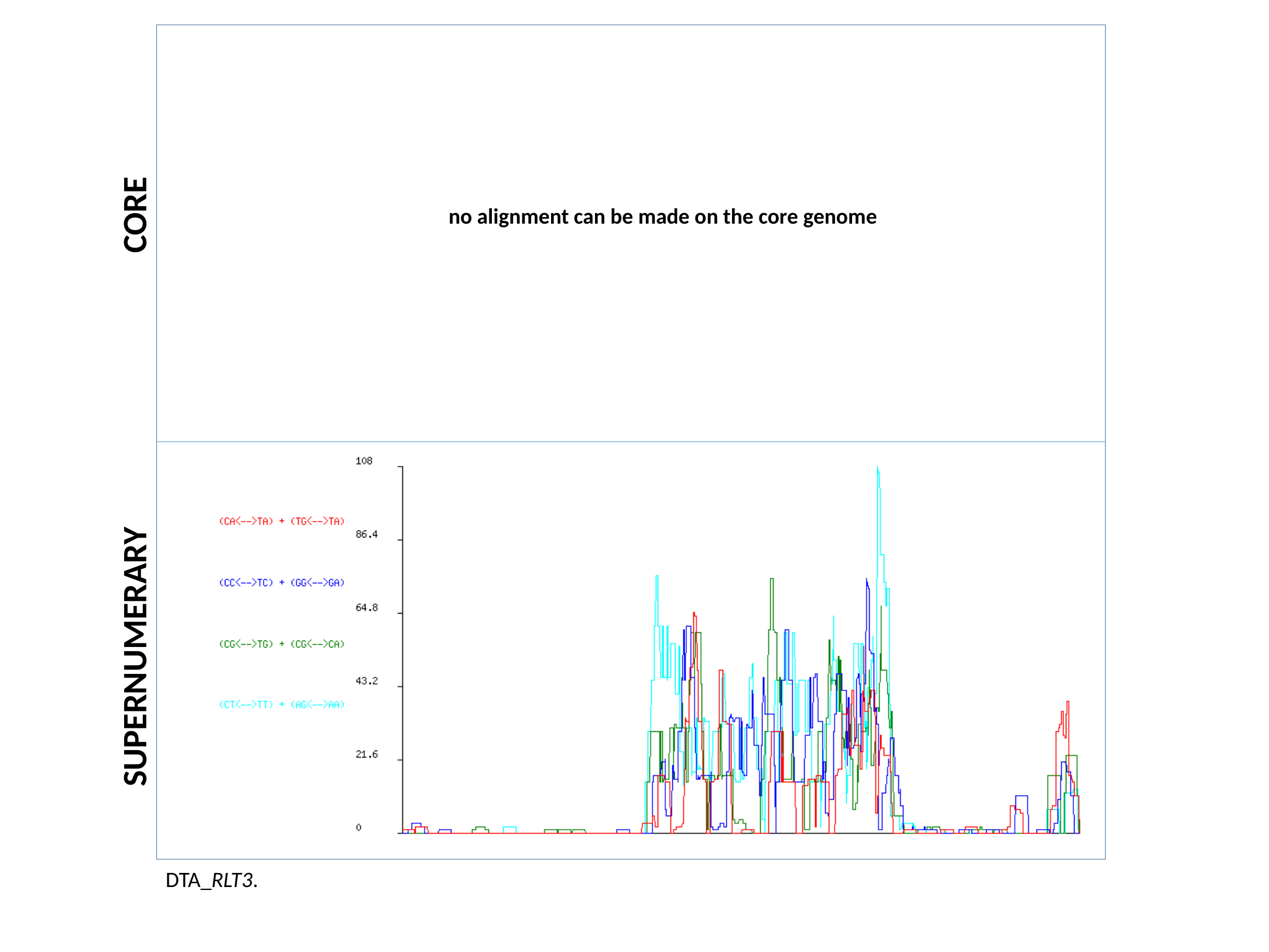

CORE
no alignment can be made on the core genome
SUPERNUMERARY
DTA_RLT3.

## Slide 23
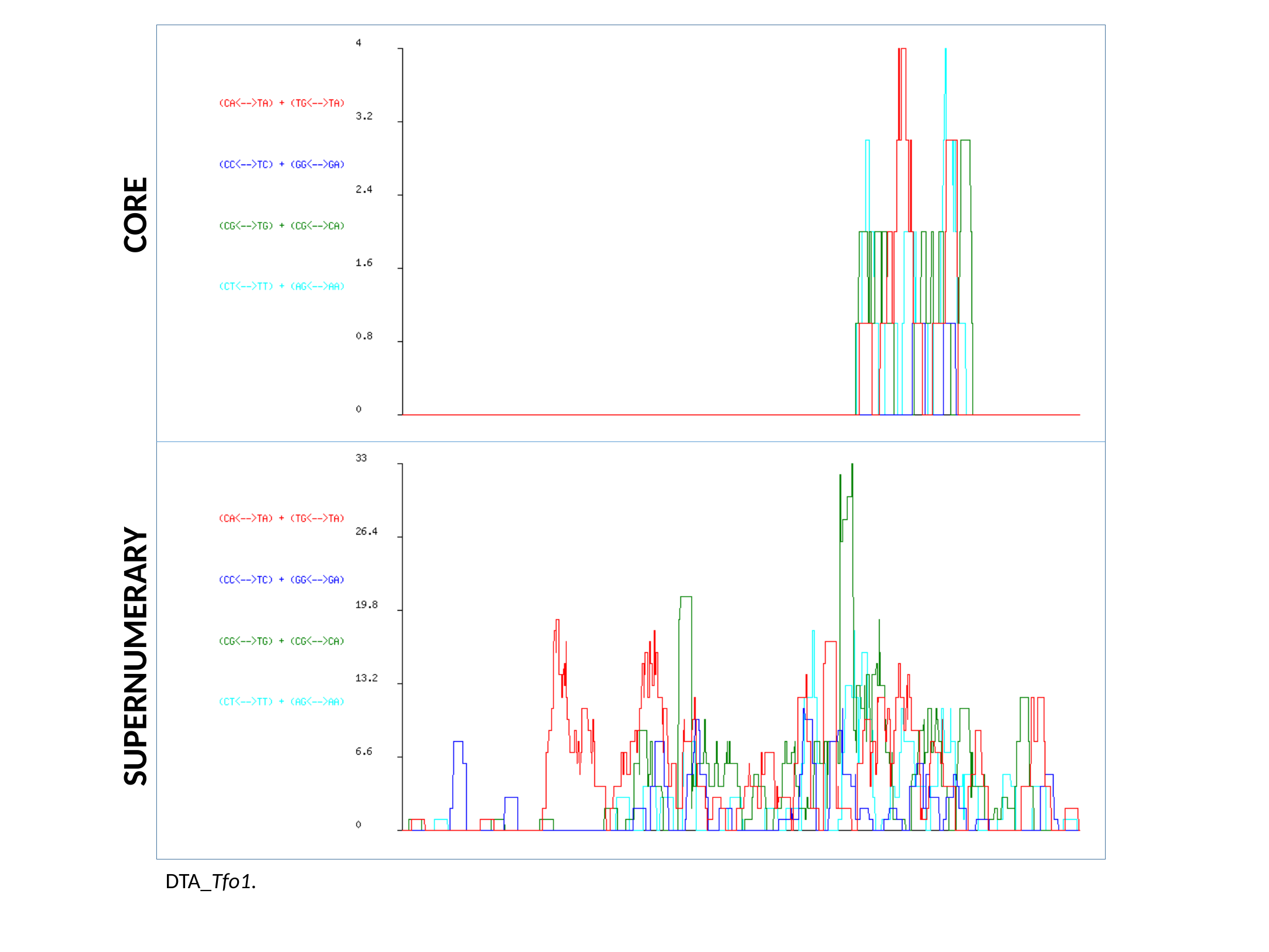

CORE
SUPERNUMERARY
DTA_Tfo1.

## Slide 24
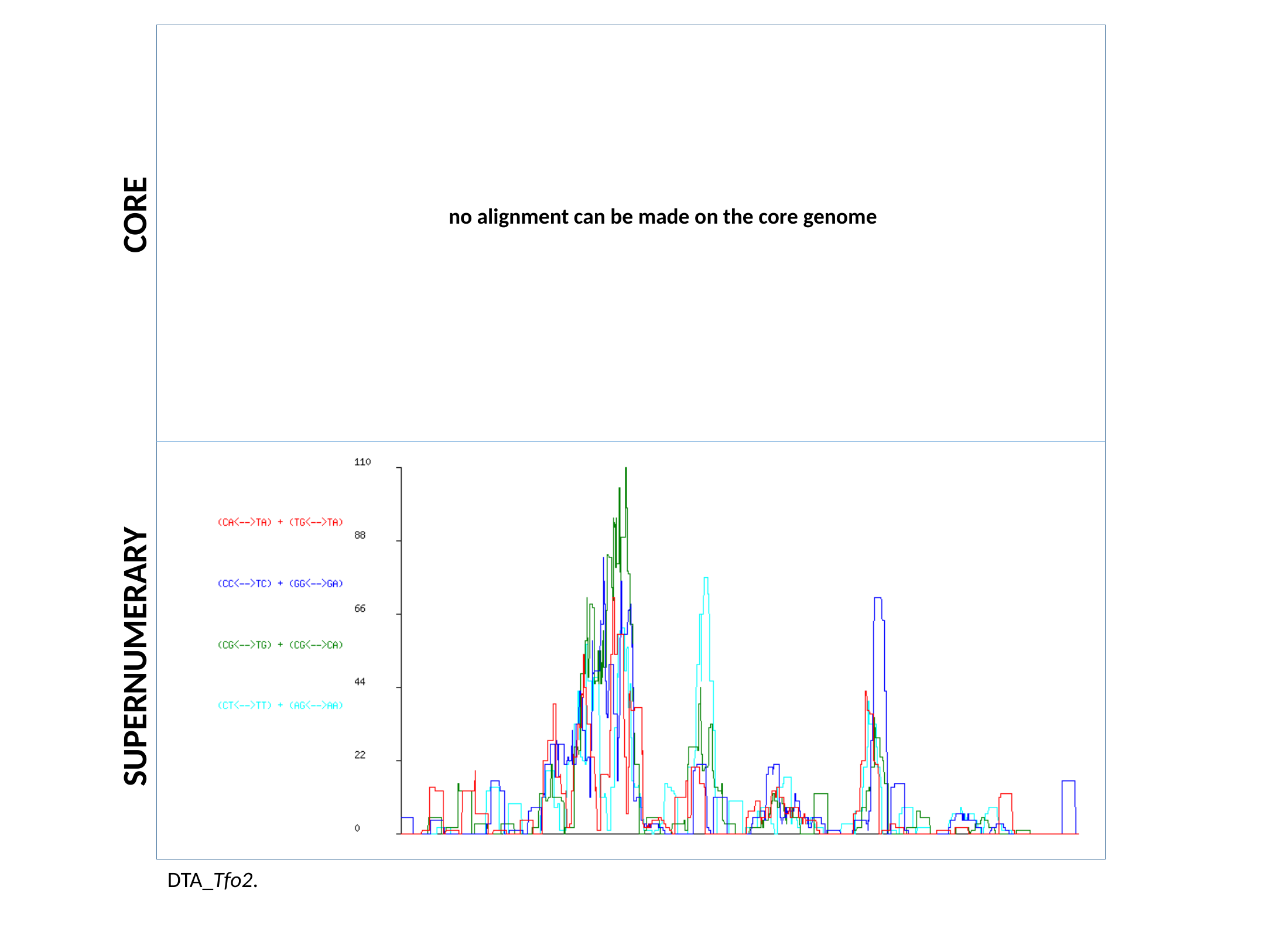

CORE
no alignment can be made on the core genome
SUPERNUMERARY
DTA_Tfo2.

## Slide 25
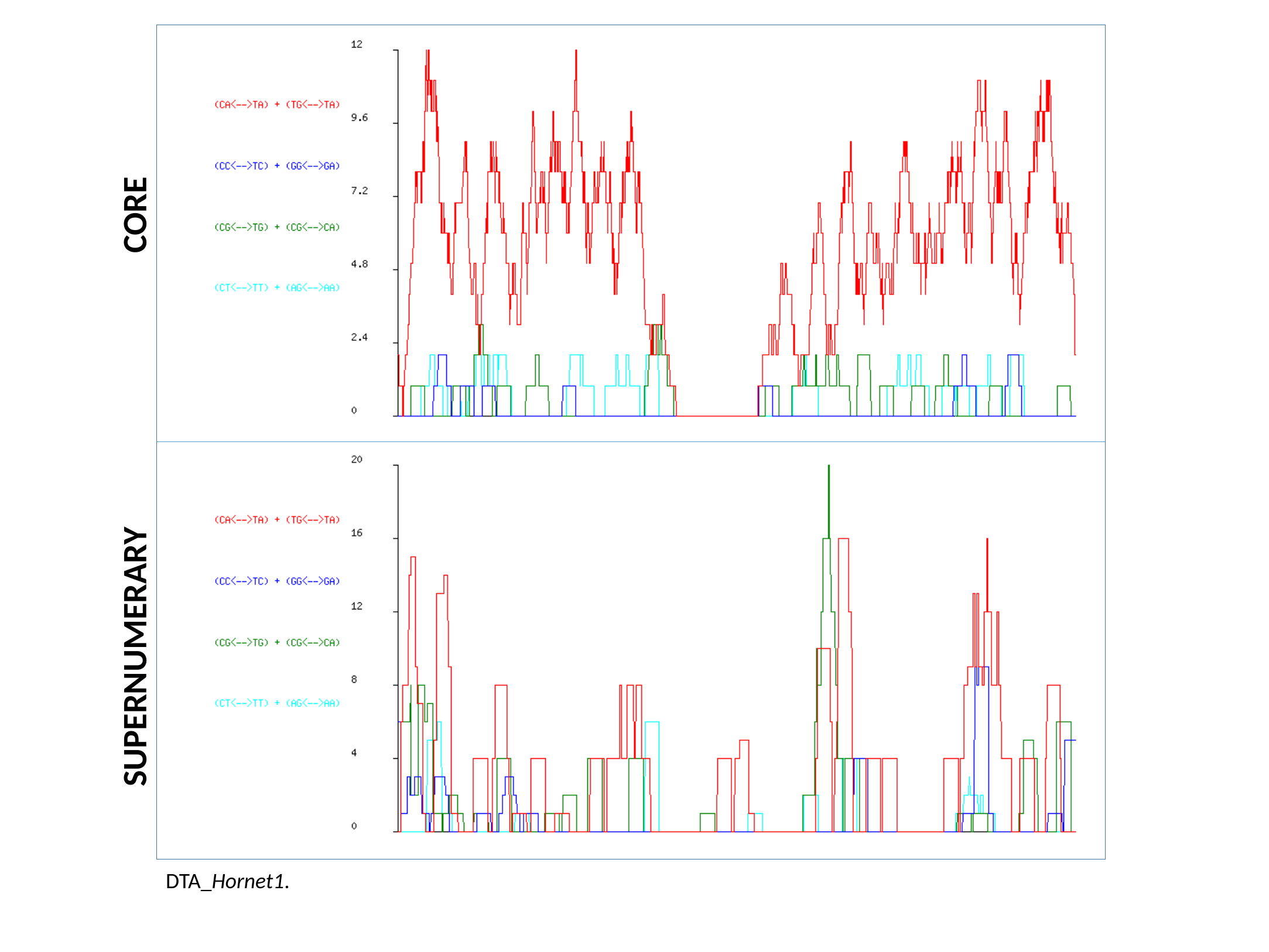

CORE
SUPERNUMERARY
DTA_Hornet1.

## Slide 26
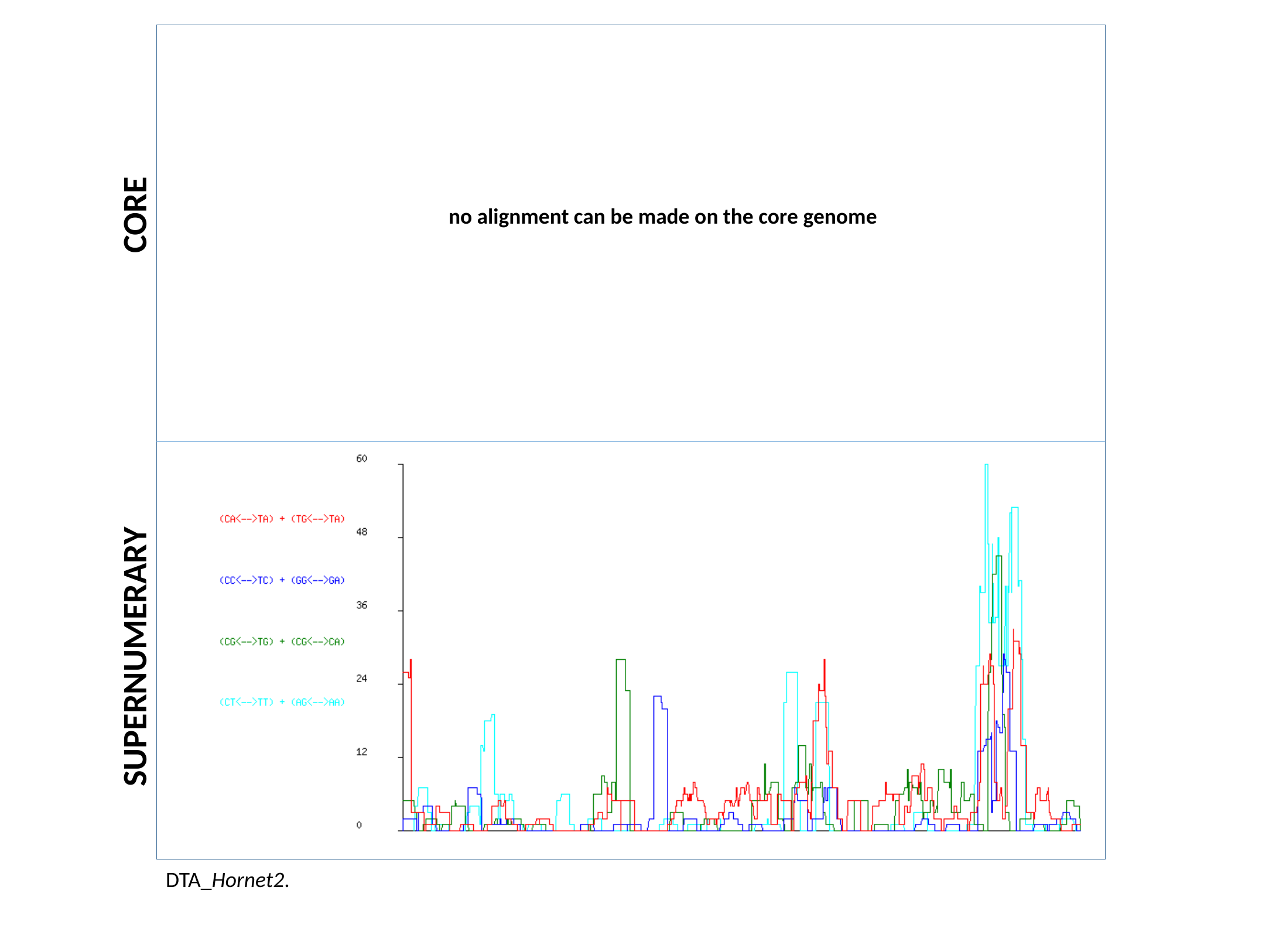

CORE
no alignment can be made on the core genome
SUPERNUMERARY
DTA_Hornet2.

## Slide 27
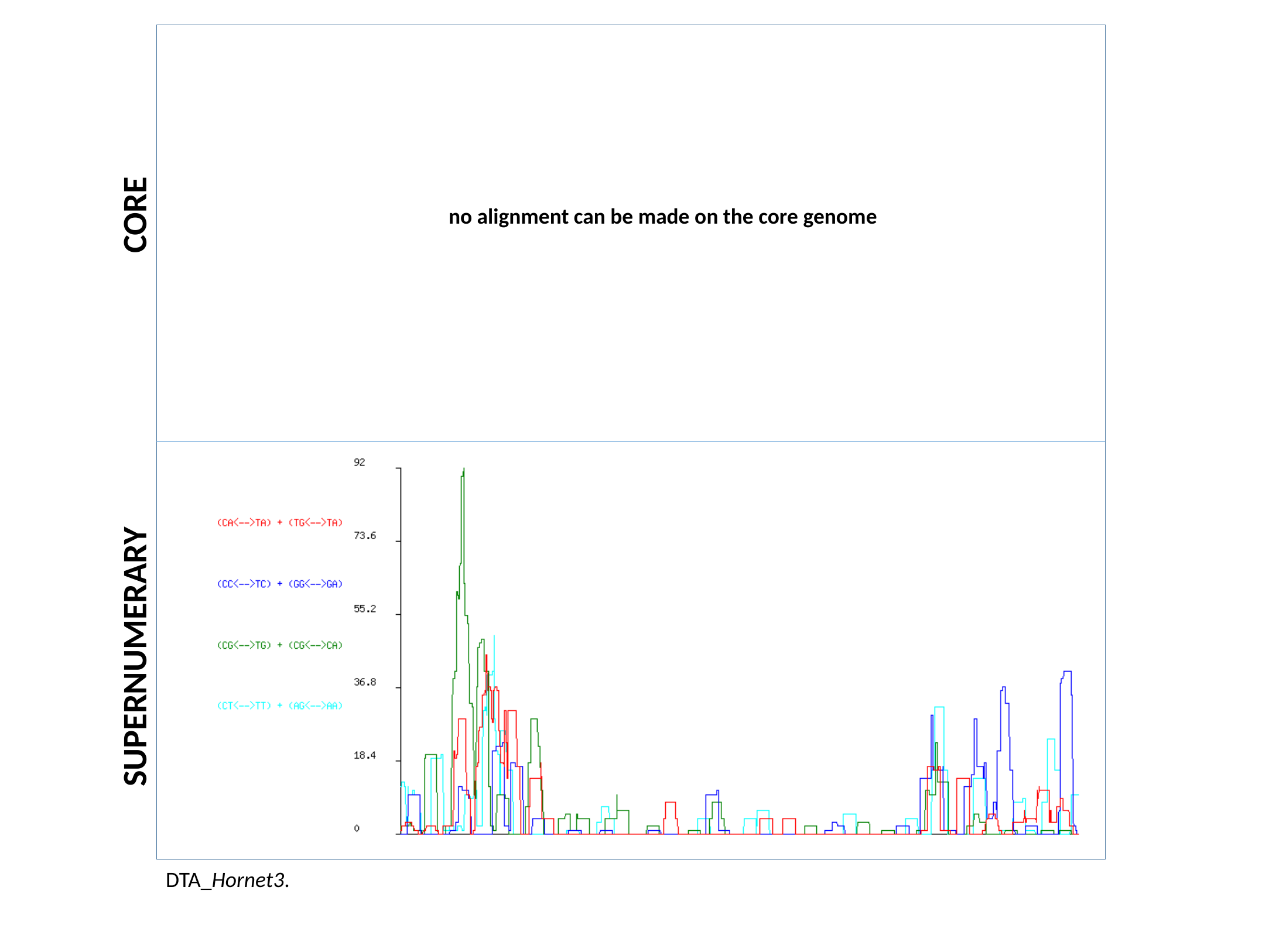

CORE
no alignment can be made on the core genome
SUPERNUMERARY
DTA_Hornet3.

## Slide 28
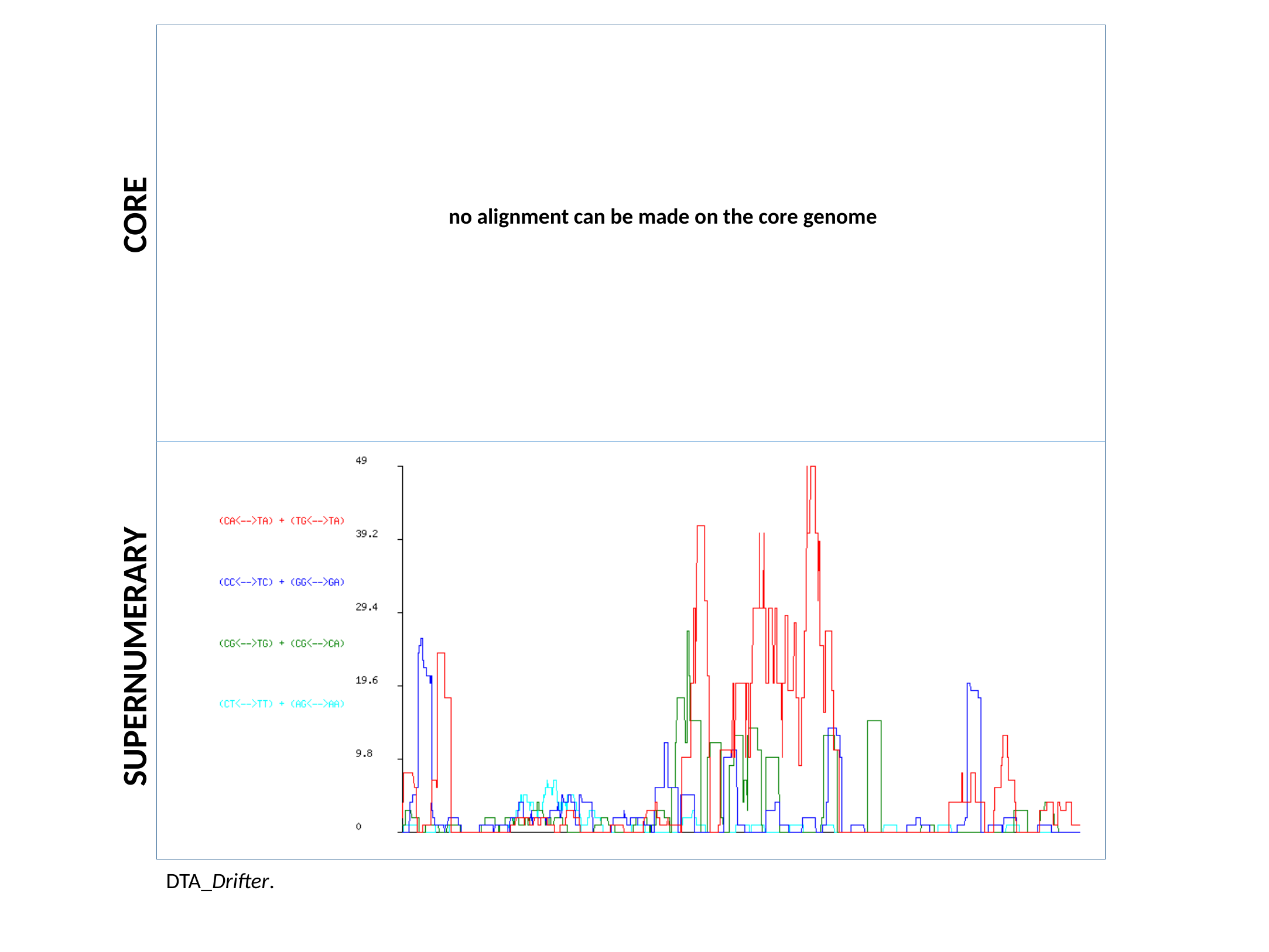

CORE
no alignment can be made on the core genome
SUPERNUMERARY
DTA_Drifter.

## Slide 29
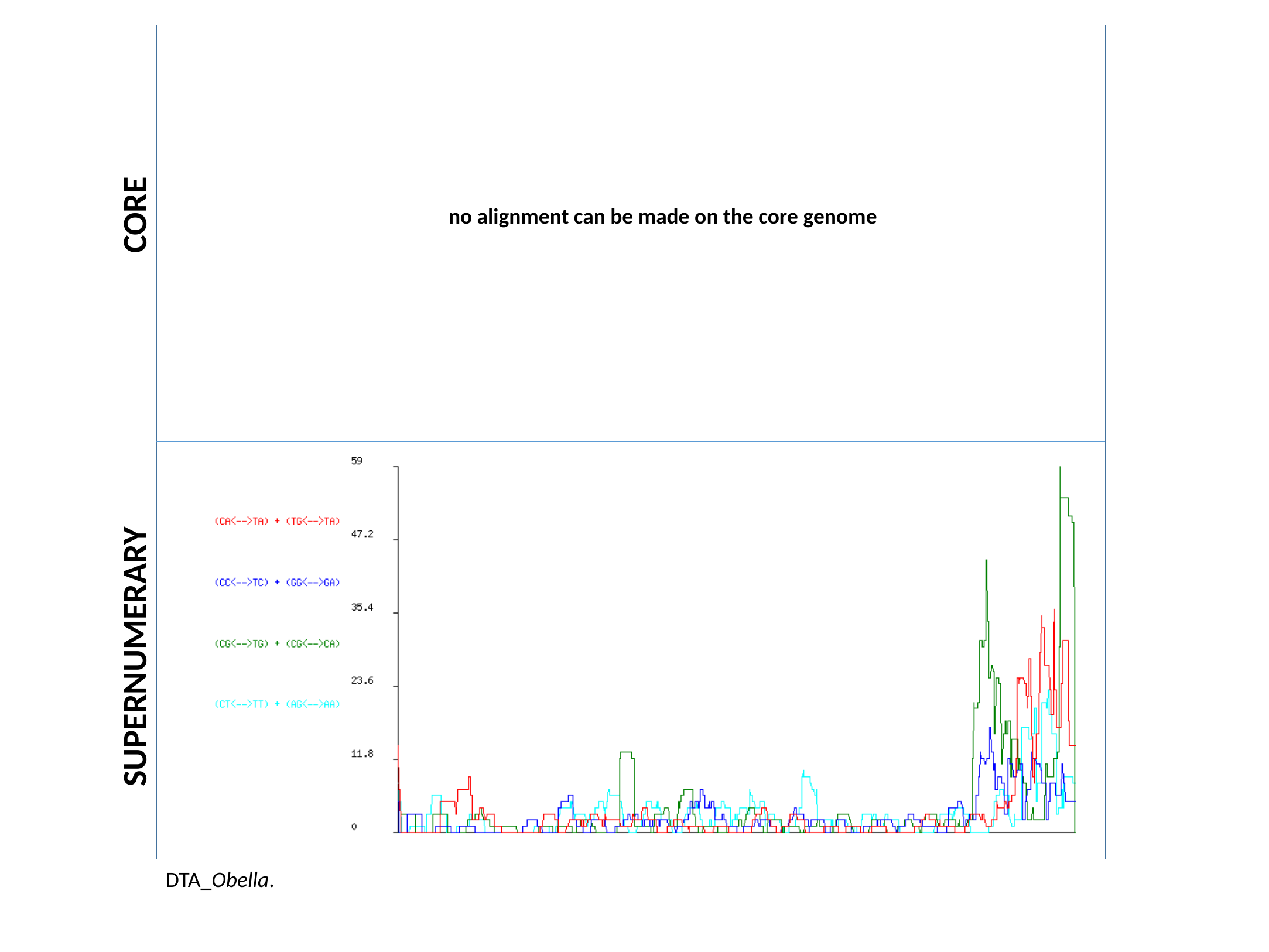

CORE
no alignment can be made on the core genome
SUPERNUMERARY
DTA_Obella.

## Slide 30
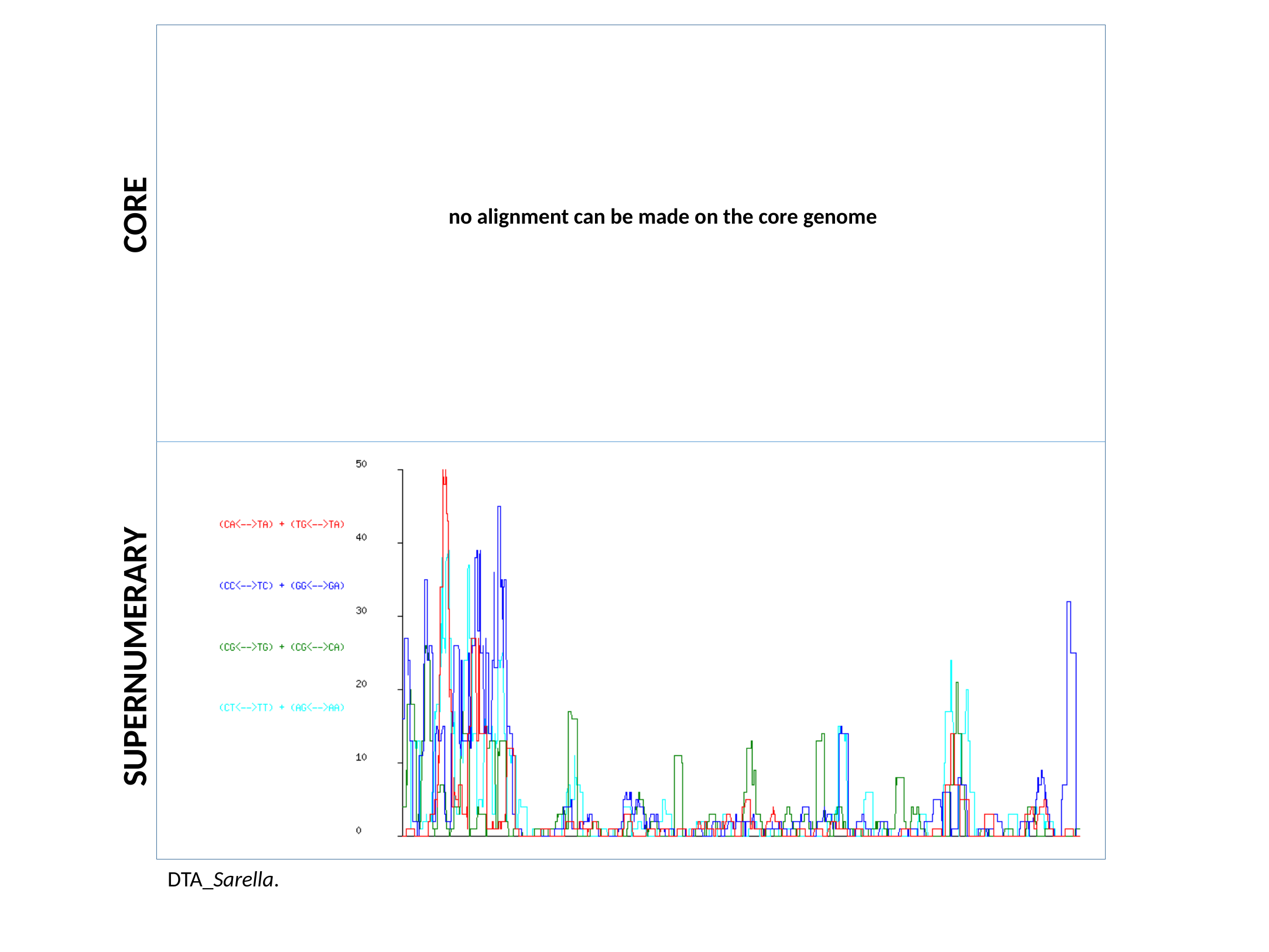

CORE
no alignment can be made on the core genome
SUPERNUMERARY
DTA_Sarella.

## Slide 31
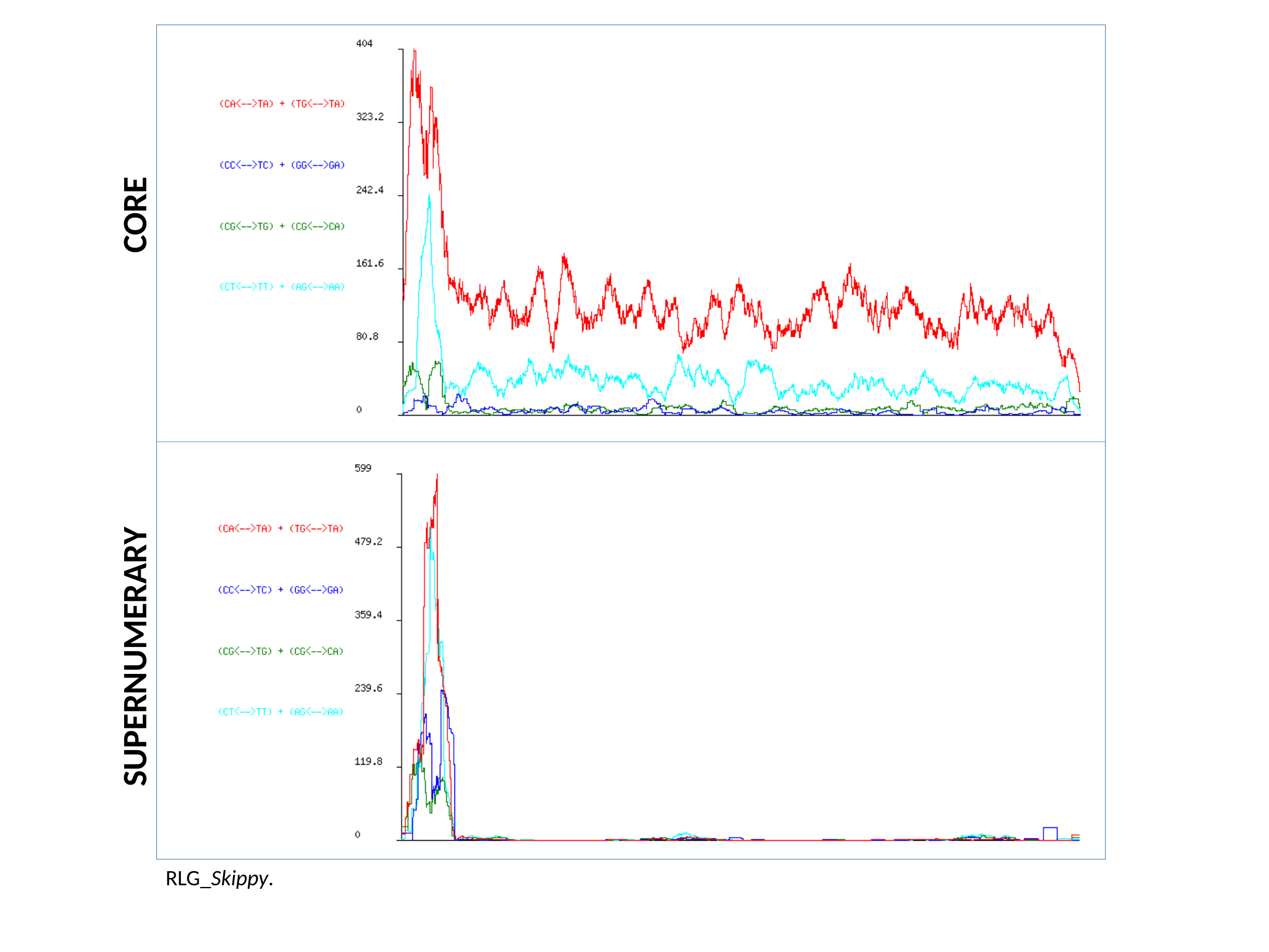

CORE
SUPERNUMERARY
RLG_Skippy.

## Slide 32
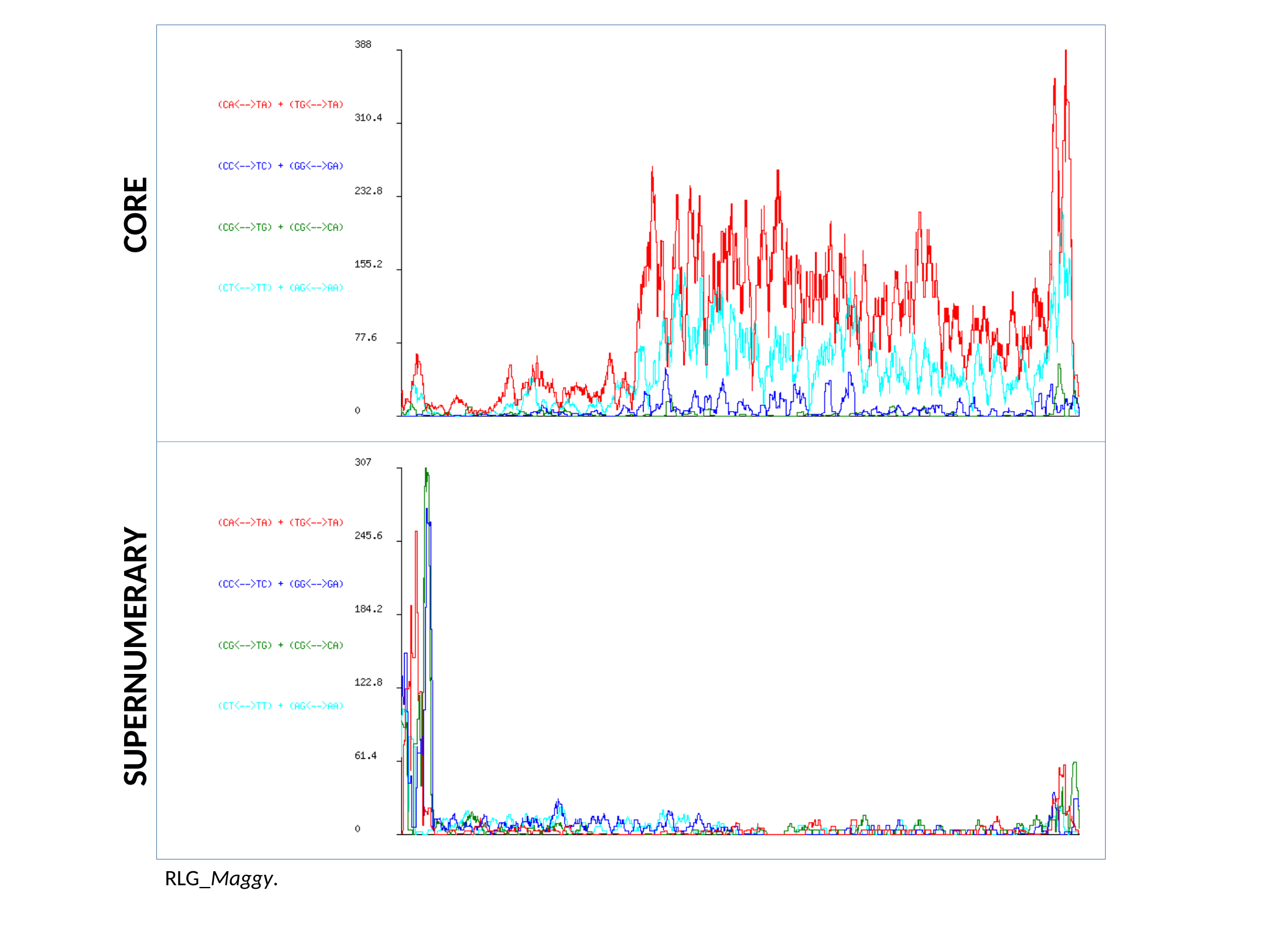

CORE
SUPERNUMERARY
RLG_Maggy.

## Slide 33
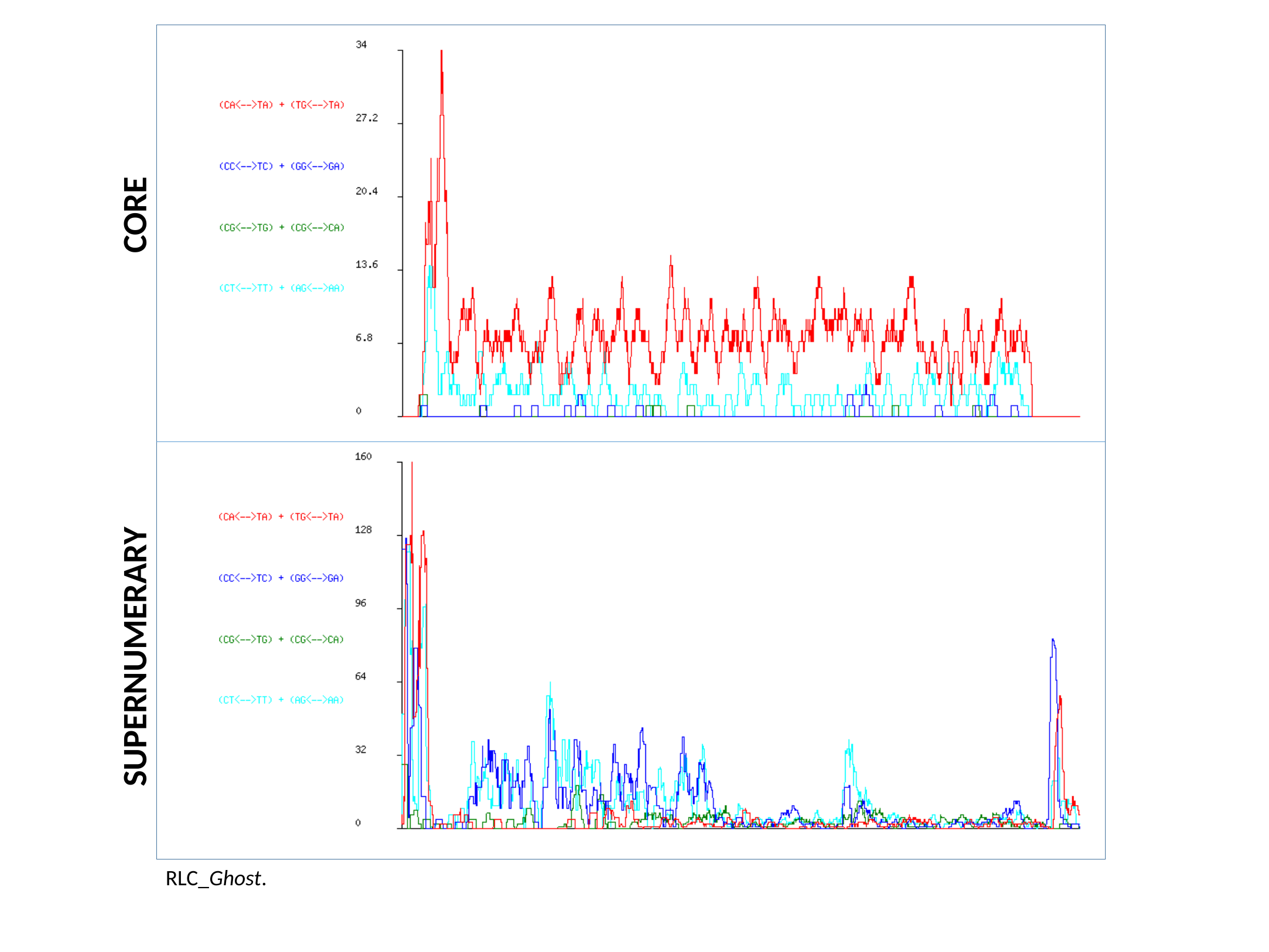

CORE
SUPERNUMERARY
RLC_Ghost.

## Slide 34
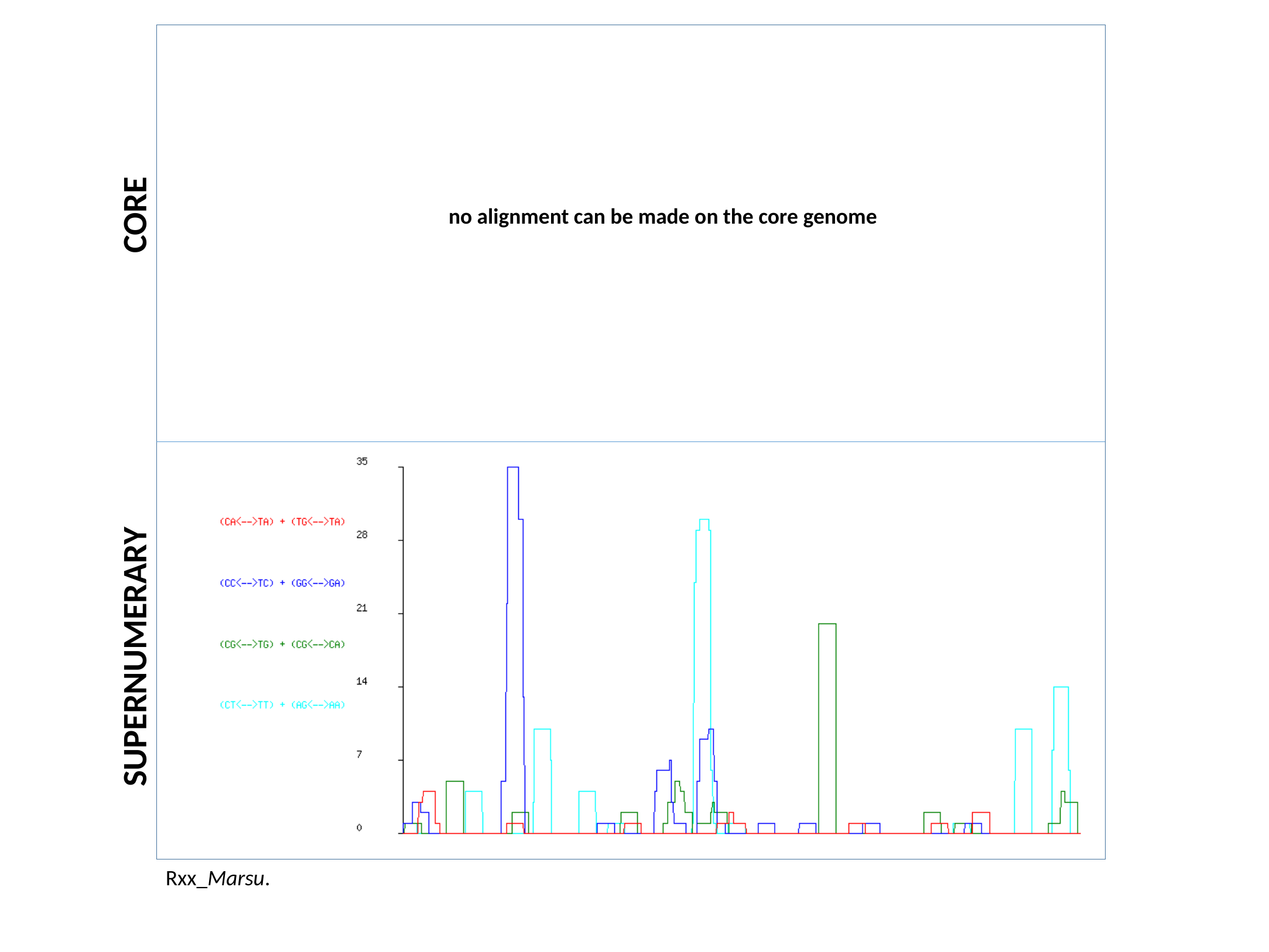

CORE
no alignment can be made on the core genome
SUPERNUMERARY
Rxx_Marsu.
