# Supplementary material for: Living apart together: crosstalk between the core and supernumerary genomes in a fungal plant pathogen
Source: BMC Genomics. 2016 Aug 23;17(1):670. doi: 10.1186/s12864-016-2941-6 (PMC4994206; doi:10.1186/s12864-016-2941-6)
Supplement: Additional file 9: — Intact elements on the core chromosomes of isolate 2516 are not in the same location in the other isolates. Integration of a RLG_Maggy element in chromosome 4 of isolate 2516. Location of the TE is shown in the RepeatMasker track. Mapping of the HiSeq reads of isolates 2516, 2548, 7555 and bfb0173 is shown. This TE is not present at the same location in the other isolates. In isolate 2548 and 7555, no reads span the borders of the element (but the element is present in other locations in the genomes of isolates 2548 and 7555, and therefore reads for this sequence do exist). In isolate bfb0173, RLG_Maggy is not present at all. Yellow indicates that the reads could have mapped to other places in the genome of isolate 2516 as well. (DOCX 768 kb) [file 12864_2016_2941_MOESM9_ESM.docx]

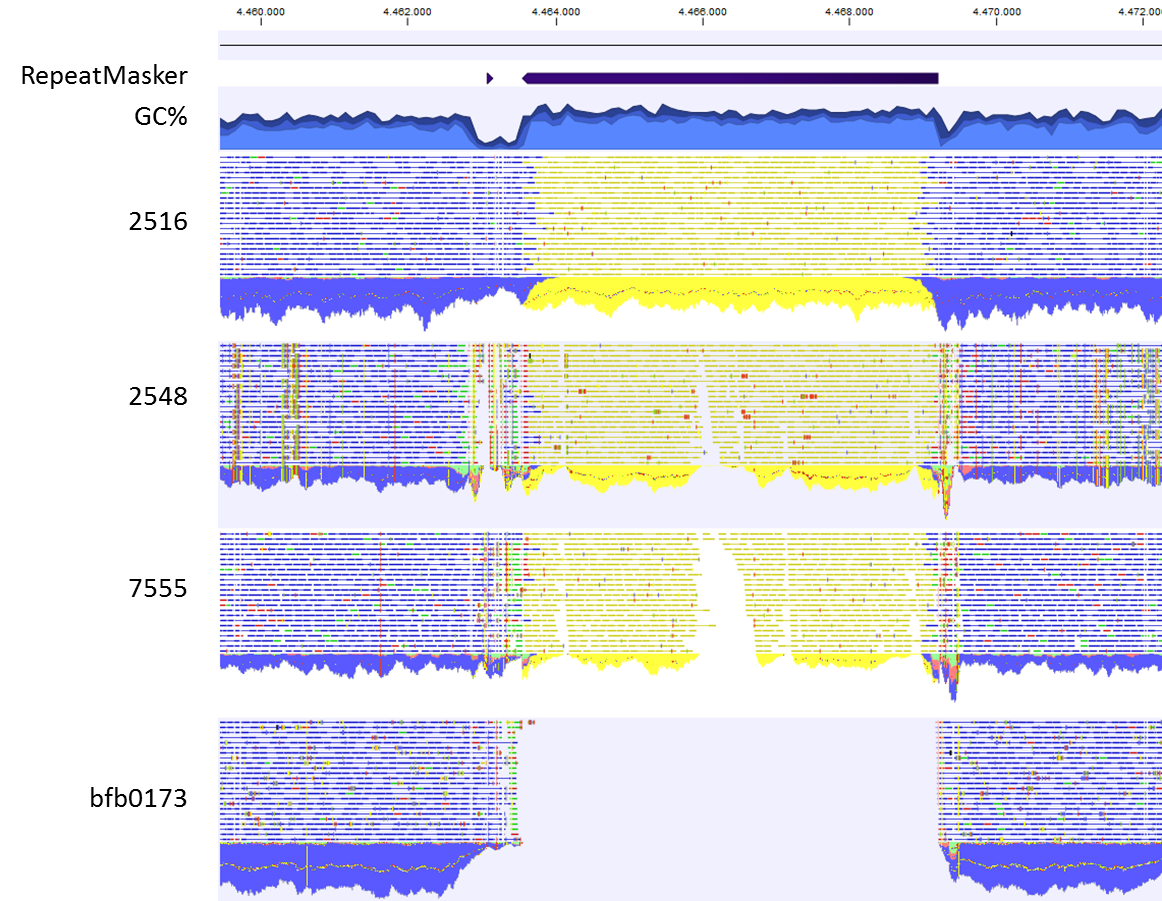


**Additional file 9** - **Intact elements on the core chromosomes of isolate 2516 are not in the same location in the other isolates.** Integration of a RLG_*Maggy* element in chromosome 4 of isolate 2516. Location of the TE is shown in the RepeatMasker track. Mapping of the HiSeq reads of isolates 2516, 2548, 7555 and bfb0173 is shown. This TE is not present at the same location in the other isolates. In isolate 2548 and 7555, no reads span the borders of the element (but the element is present in other locations in the genomes of isolates 2548 and 7555, and therefore reads for this sequence do exist). In isolate bfb0173, RLG_*Maggy* is not present at all. Yellow indicates that the reads could have mapped to other places in the genome of isolate 2516 as well.
