# Supplementary material for: Living apart together: crosstalk between the core and supernumerary genomes in a fungal plant pathogen
Source: BMC Genomics. 2016 Aug 23;17(1):670. doi: 10.1186/s12864-016-2941-6 (PMC4994206; doi:10.1186/s12864-016-2941-6)
Supplement: Additional file 10: — TE integration and genome coverage on three supernumerary contigs. Integration of intact TEs on supernumerary contigs 668, 561 and 550. The graphs shows in a sliding 1 kb window the fraction of bases from the reference contig that is covered by HiSeq reads of every isolate (value between 0 and 1). The upper track shows all TEs on contig 668, 561 and 550 of isolate 2516 that are >1 kb and >90 % identity to the element prototype (Additional file 5) with yellow dots. This TE landscape was used for comparison with isolates 2548, 7555 and bfb0173. Dots for these three isolates indicate elements for which there is read mapping that an element has integrated in the exact same location as the element in isolate 2516 (and is therefore ancestral). Dots that align vertically are conserved in multiple isolates. (DOCX 261 kb) [file 12864_2016_2941_MOESM10_ESM.docx]

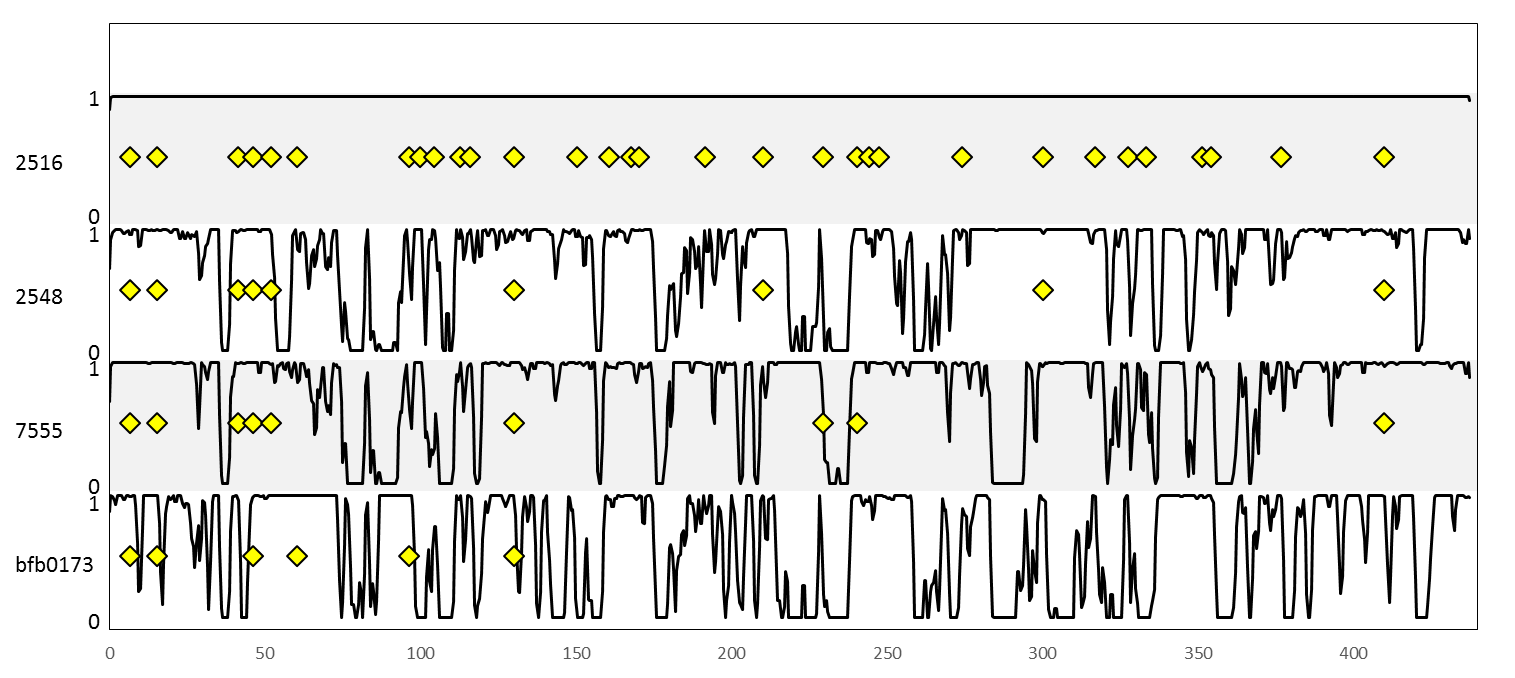


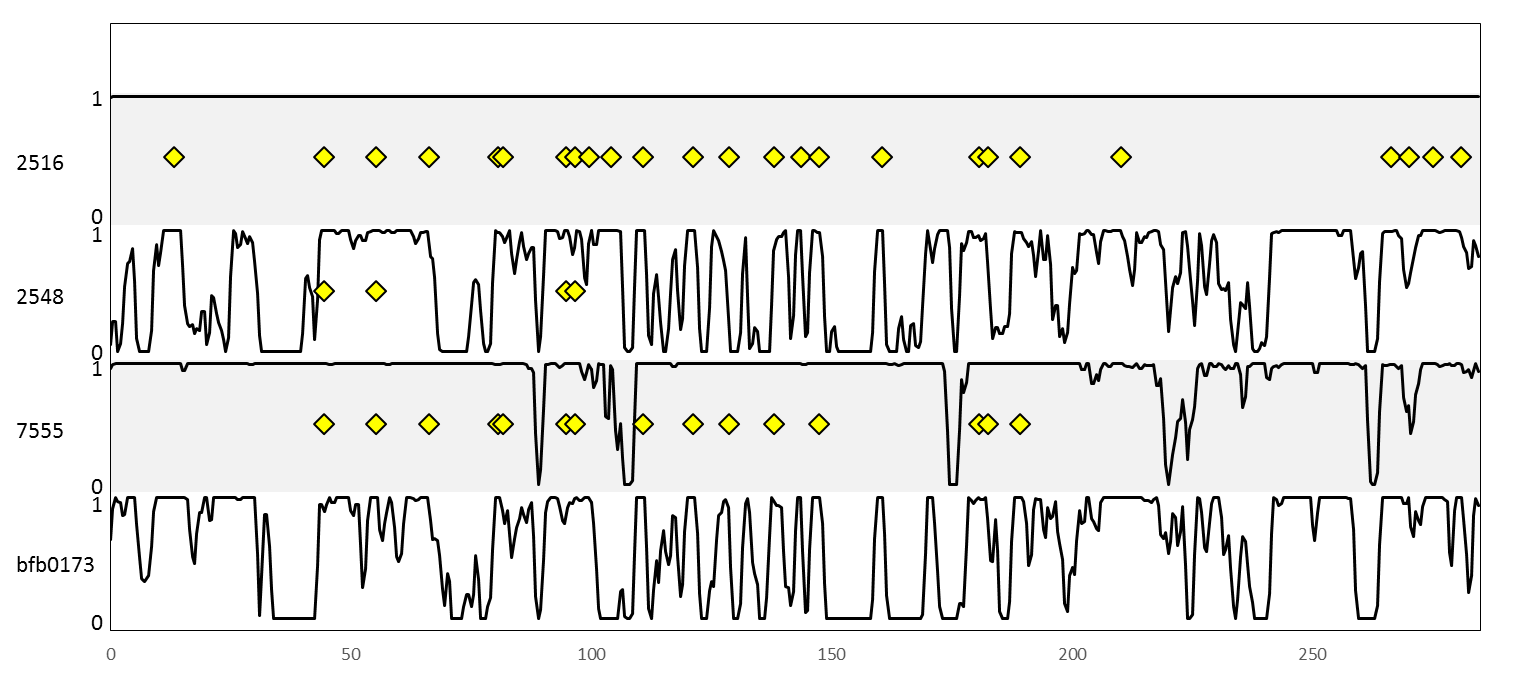


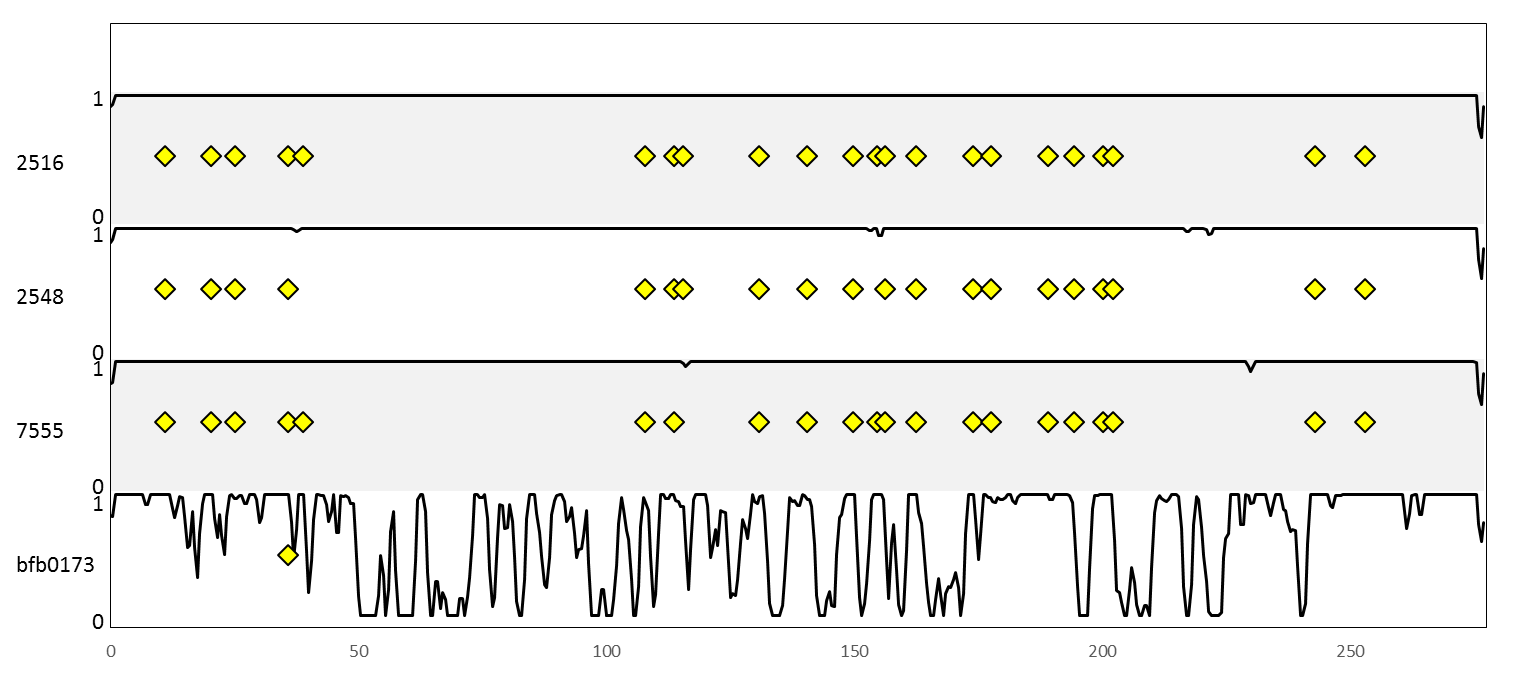


**Additional file 10** - **TE integration and genome coverage on three supernumerary contigs.** Integration of intact TEs on supernumerary contigs 668, 561 and 550. The graphs shows in a sliding 1kb window the fraction of bases from the reference contig that is covered by HiSeq reads of every isolate (value between 0 and 1). The upper track shows all TEs on contig 668, 561 and 550 of isolate 2516 that are >1kb and >90% identity to the element prototype (Text File S1) with yellow dots. This TE landscape was used for comparison with isolates 2548, 7555 and bfb0173. Dots for these three isolates indicate elements for which there is read mapping that an element has integrated in the exact same location as the element in isolate 2516 (and is therefore ancestral). Dots that align vertically are conserved in multiple isolates.
