# Supplementary material for: Living apart together: crosstalk between the core and supernumerary genomes in a fungal plant pathogen
Source: BMC Genomics. 2016 Aug 23;17(1):670. doi: 10.1186/s12864-016-2941-6 (PMC4994206; doi:10.1186/s12864-016-2941-6)
Supplement: Additional file 11: — Non-cumulative integrations of TEs on the supernumerary genome. Four instances are shown where a transposable element is in the same location for isolate 2516 and one or more other isolates. Tracks from top to bottom: RepeatMasker output, HiSeq reads from isolate 2516, HiSeq reads from isolate 2548, HiSeq reads from isolate 7555, and HiSeq reads from isolate bfb0173. Reads that can map to more than one location in the genome are automatically colored yellow in CLC Genomics Workbench. First screenshot: a DTA_Nymeria element on contig 550 is shared between 2516, 2548 and 7555. Second screenshot: a DTF_Fot2 element is shared between 2516, 2548 and 7555 on contig 550. Third screenshot: A DTF_Fot3-B element has inserted into a DTA_RLT2 element on contig 308. The DTA_RLT2 element is shared between 2516, 7555 and bfb0173. The insertion of DTF_Fot3-B is shared between 2516 and bfb0173. Fourth screenshot: a RLG_Skippy element on contig 308 is partially shared between 2516 and bfb0173 (only downstream flank has convincing read support). (DOCX 622 kb) [file 12864_2016_2941_MOESM11_ESM.docx]

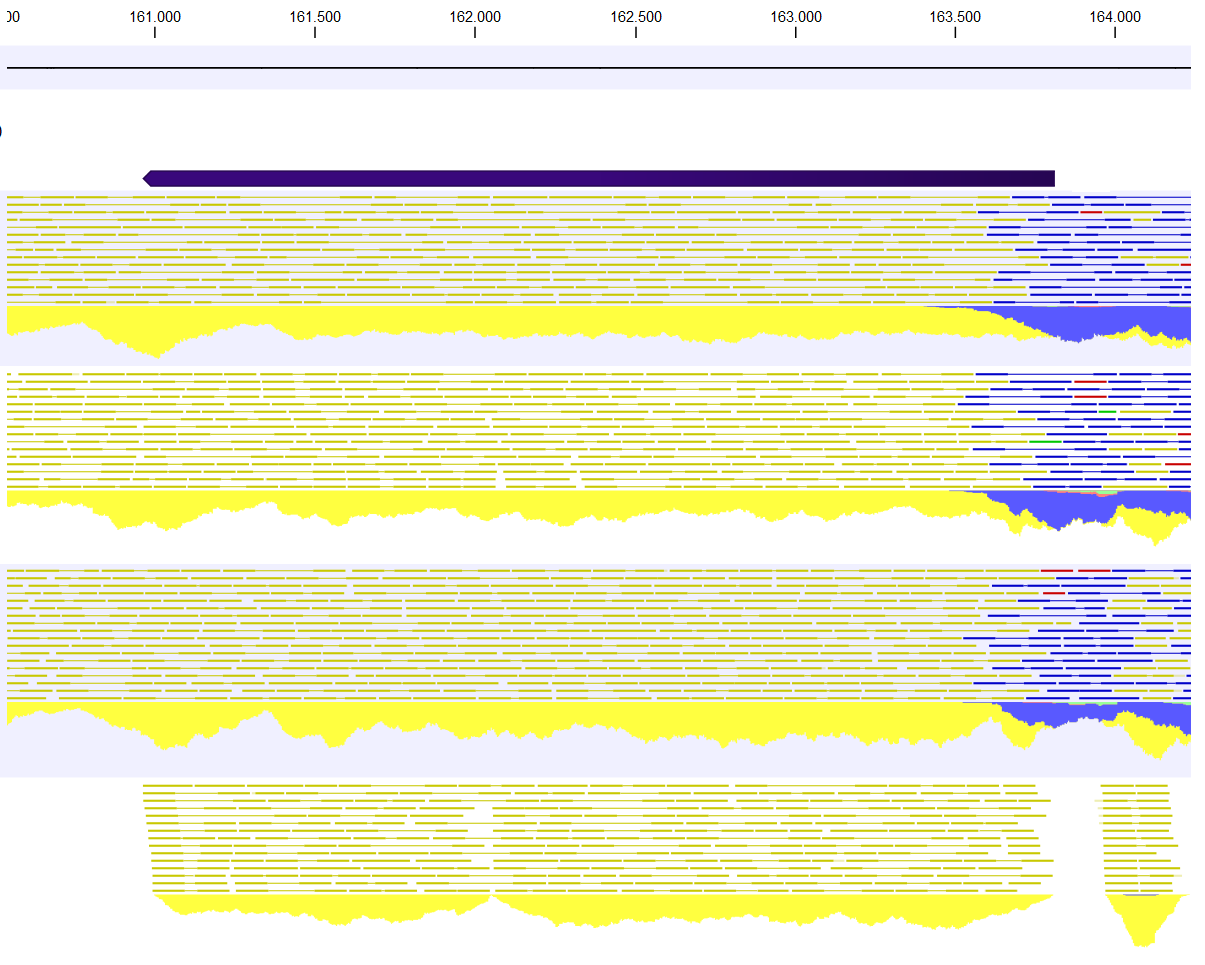


2516

2548

7555

bfb0173

DTA_*Nymeria*


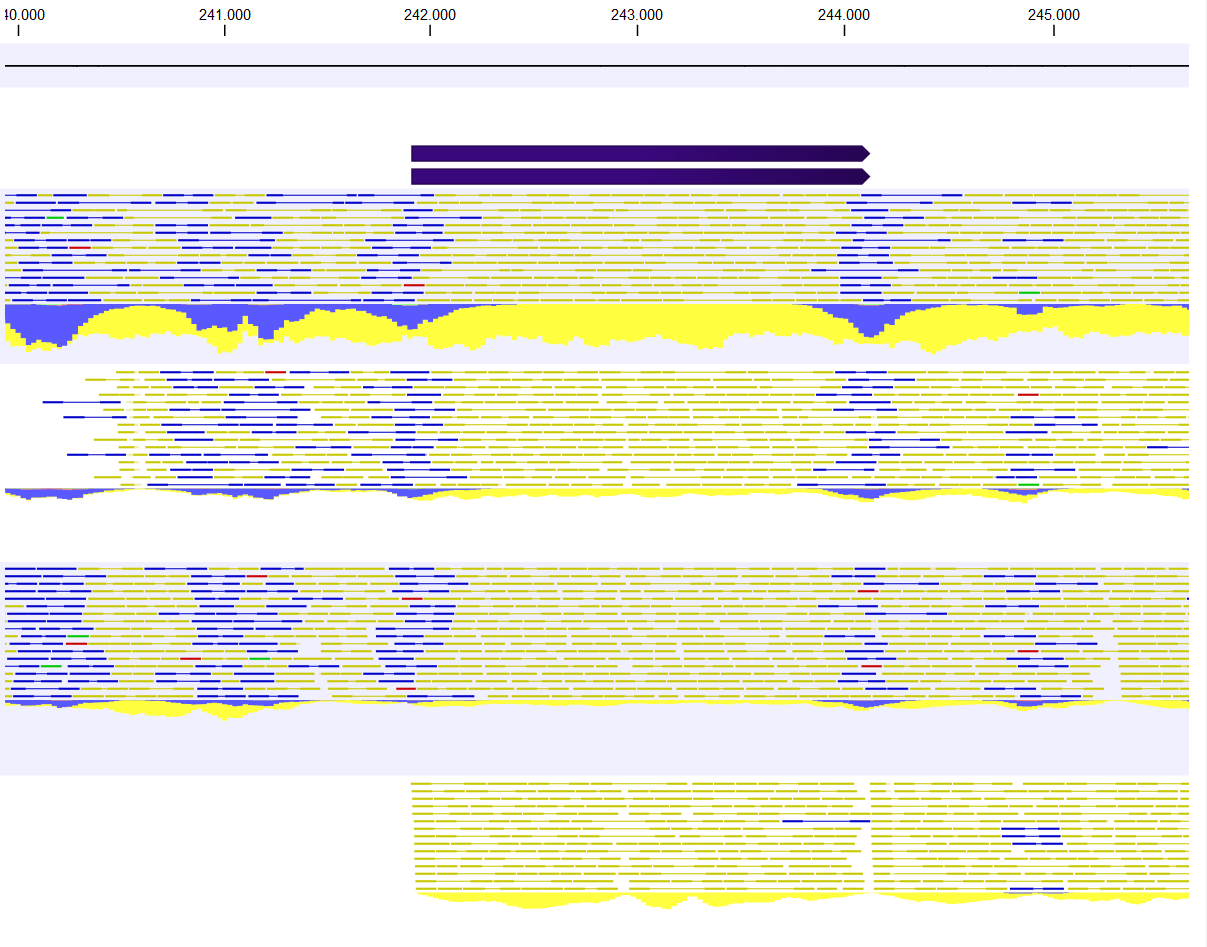


2516

2548

7555

bfb0173

DTF_*Fot2*


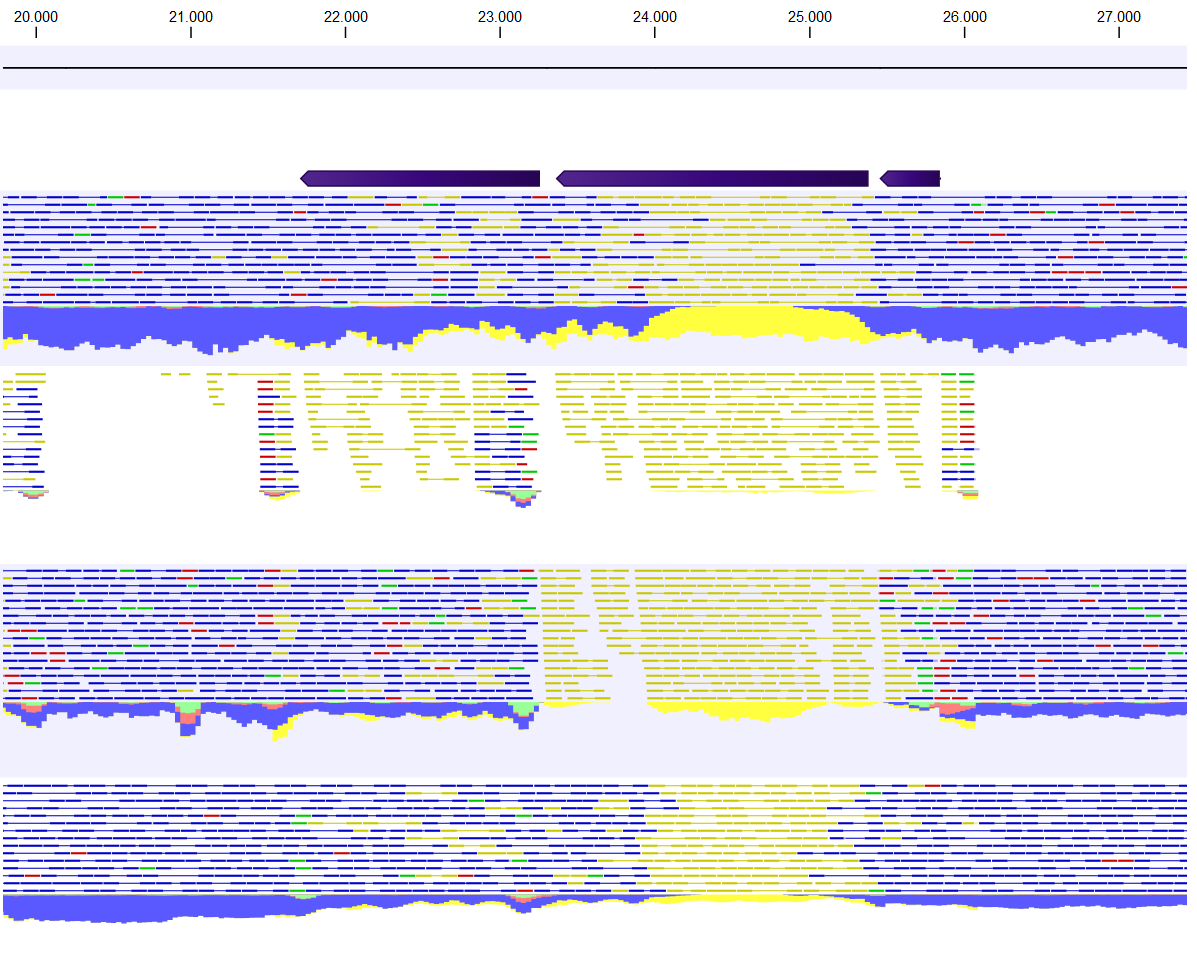


2516

2548

7555

bfb0173

DTF_*Fot3-B*

DTA_*RLT2*

DTA_*RLT2*


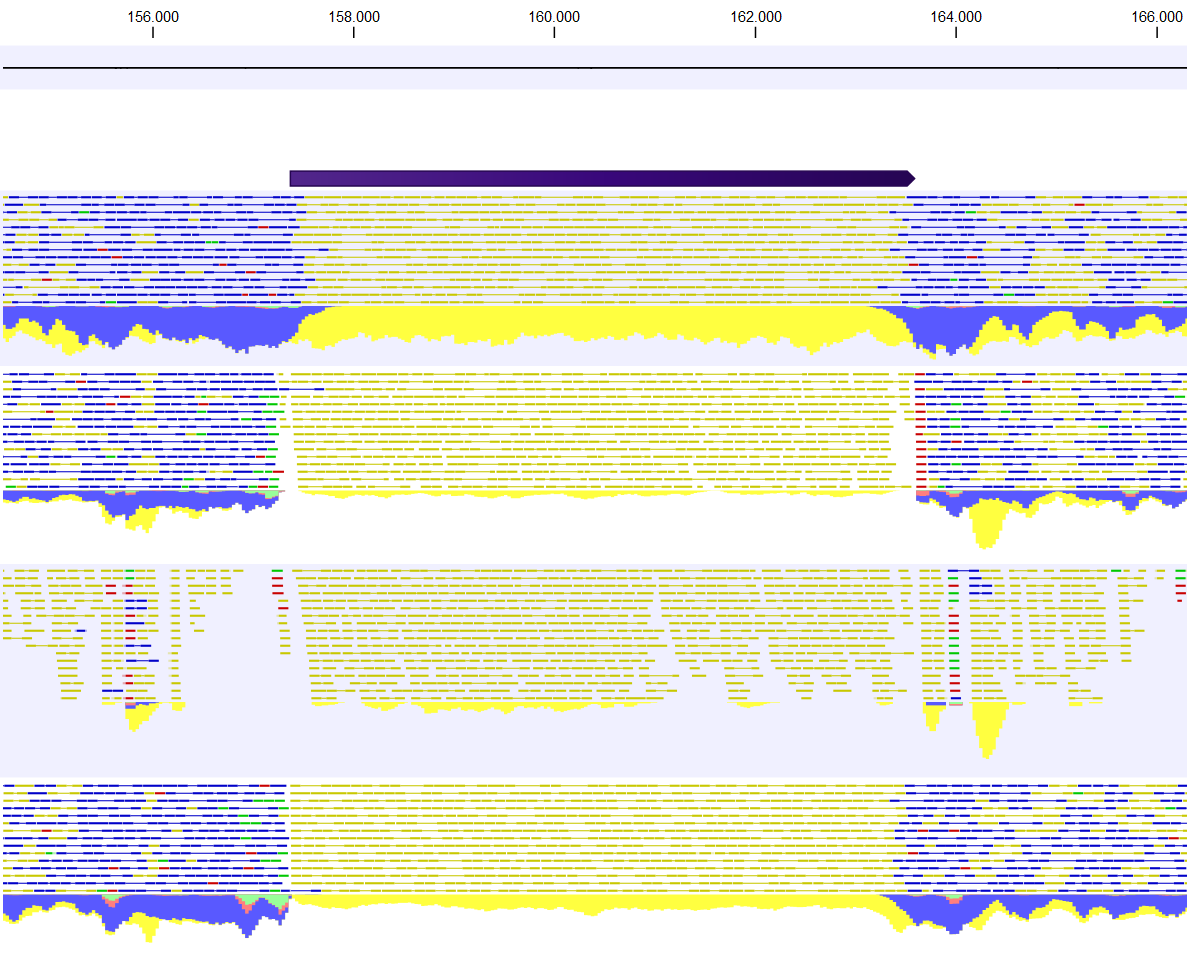


2516

2548

7555

bfb0173

RLG_*Skippy*

**Additional file 11** - **Non-cumulative integrations of TEs on the supernumerary genome.** Four instances are shown where a transposable element is in the same location for isolate 2516 and one or more other isolates. Tracks from top to bottom: RepeatMasker output, HiSeq reads from isolate 2516, HiSeq reads from isolate 2548, HiSeq reads from isolate 7555, and HiSeq reads from isolate bfb0173. Reads that can map to more than one location in the genome are automatically colored yellow in CLC Genomics Workbench. First screenshot: a DTA_*Nymeria* element on contig 550 is shared between 2516, 2548 and 7555. Second screenshot: a DTF_*Fot2* element is shared between 2516, 2548 and 7555 on contig 550. Third screenshot: A DTF_*Fot3-B* element has inserted into a DTA_*RLT2* element on contig 308. The DTA_*RLT2* element is shared between 2516, 7555 and bfb0173. The insertion of DTF_*Fot3-B* is shared between 2516 and bfb0173. Fourth screenshot: a RLG_*Skippy* element on contig 308 is partially shared between 2516 and bfb0173 (only downstream flank has convincing read support).
