# Supplementary material for: Living apart together: crosstalk between the core and supernumerary genomes in a fungal plant pathogen
Source: BMC Genomics. 2016 Aug 23;17(1):670. doi: 10.1186/s12864-016-2941-6 (PMC4994206; doi:10.1186/s12864-016-2941-6)
Supplement: Additional file 14: — Representation of both a genuine (top) and double assembled gene duplication (middle and bottom). Upper track in every panel: mapping of the SMRT reads. Second track: CDS annotations of the reference genome. Lower track: mapping of the HiSeq reads for the reference isolate. Upper panel: two identical genes (circled in black, g12962 and g12967) on contig 459, separated by 20 kb of sequence. Read mapping shows contiguous sequence without assembly mistakes. Middle and bottom panel: identical genes (circled in black) are present at respectively the end of contig 440 (at 17 kb of the 23 kb contig) and the beginning of contig 441 (at 6 kb into the contig). Note the untangling of the reads near the end of the contig 440, where the assembler presumably stalled. The environment of the “duplicated genes” is identical in both instances. This is likely a case of double assembly. (DOCX 408 kb) [file 12864_2016_2941_MOESM14_ESM.docx]

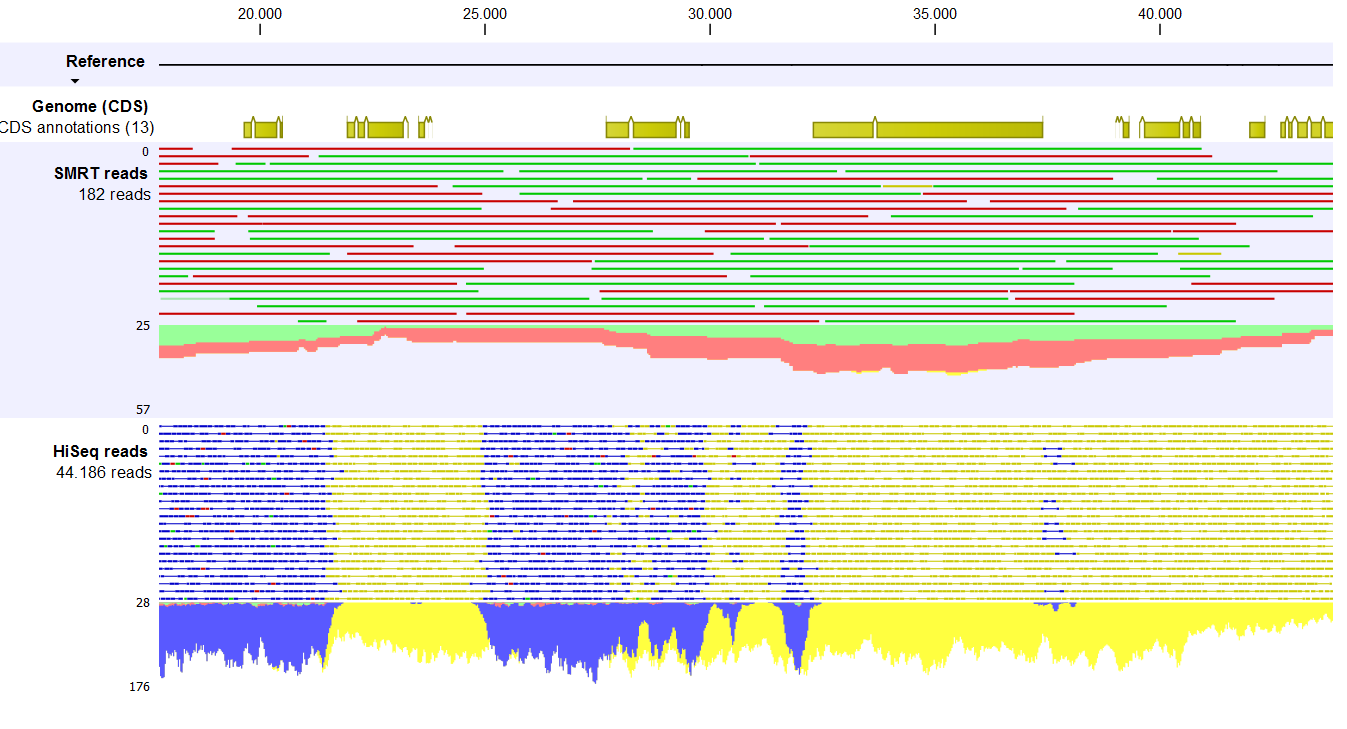


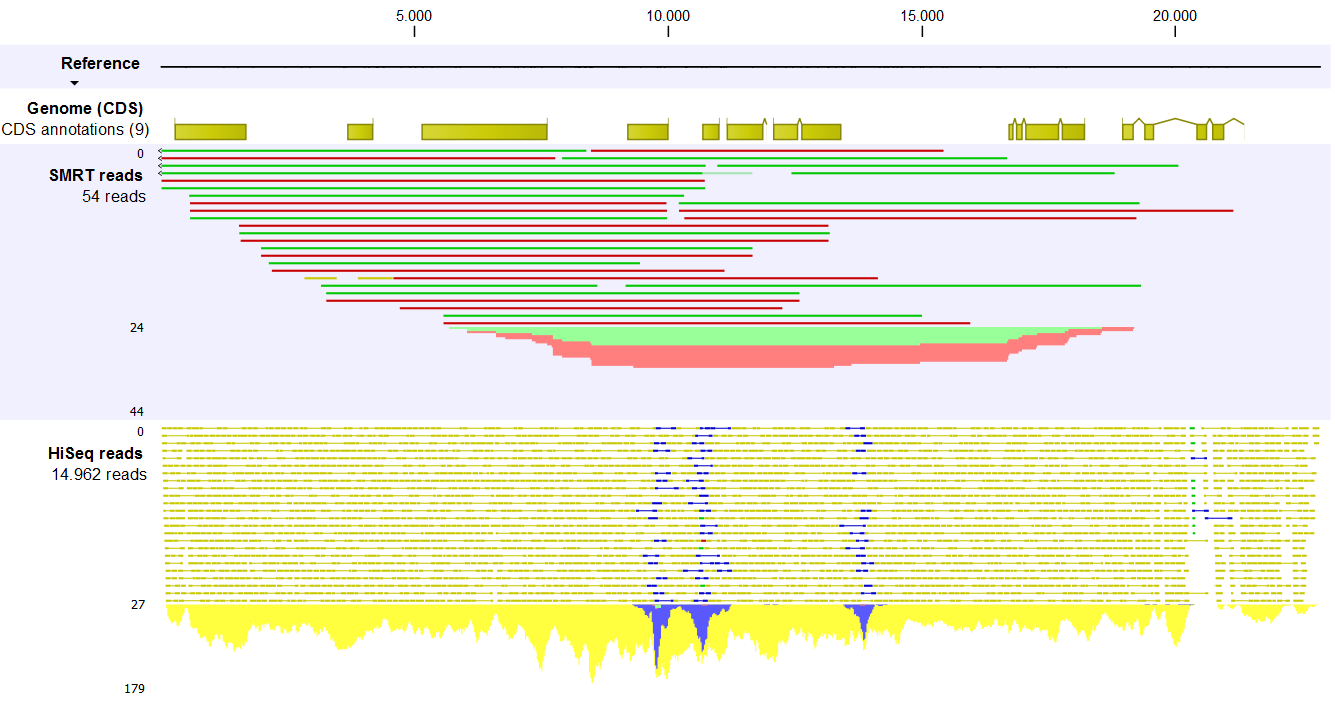


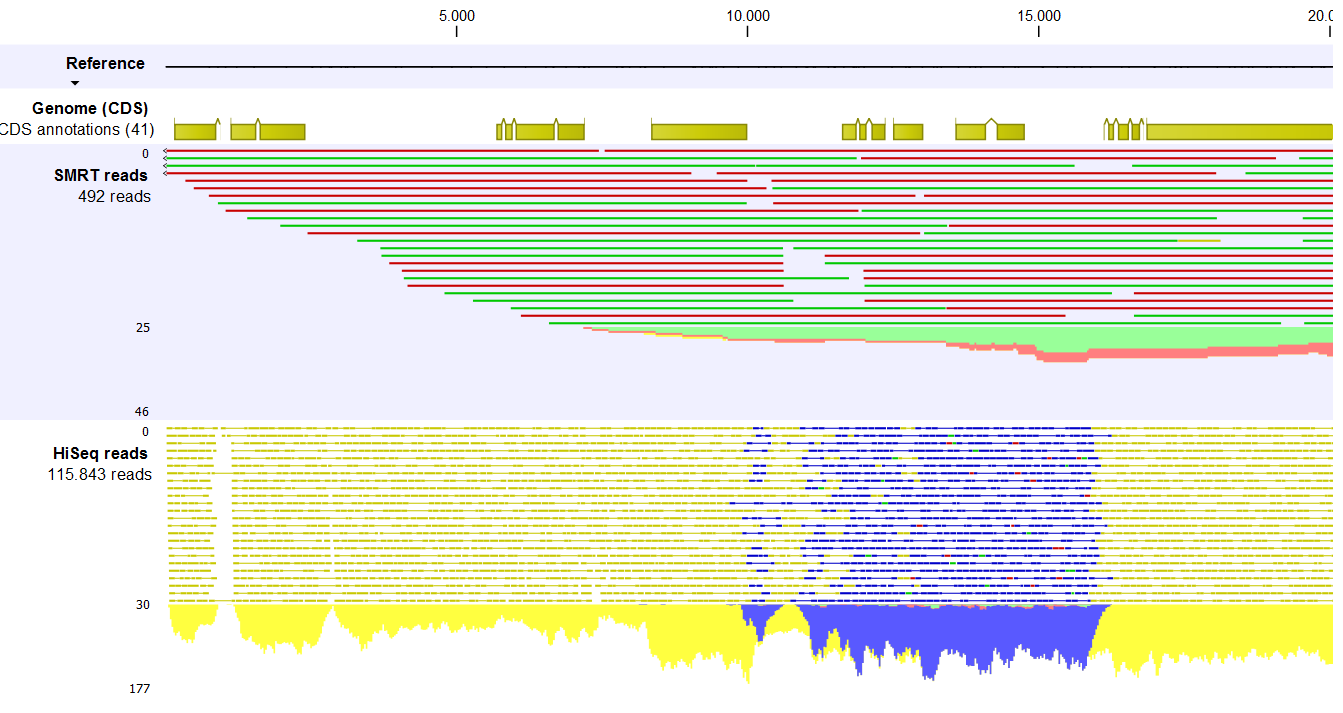


**Additional file 14** – **Representation of both a genuine (top) and double assembled gene duplication (middle and bottom).** Upper track in every panel: mapping of the SMRT reads. Second track: CDS annotations of the reference genome. Lower track: mapping of the HiSeq reads for the reference isolate. Upper panel: two identical genes (circled in black, g12962 and g12967) on contig 459, separated by 20kb of sequence. Read mapping shows contiguous sequence without assembly mistakes. Middle and bottom panel: identical genes (circled in black) are present at respectively the end of contig 440 (at 17kb of the 23kb contig) and the beginning of contig 441 (at 6kb into the contig). Note the untangling of the reads near the end of the contig 440, where the assembler presumably stalled. The environment of the “duplicated genes” is identical in both instances. This is likely a case of double assembly.
