# Supplementary material for: Living apart together: crosstalk between the core and supernumerary genomes in a fungal plant pathogen
Source: BMC Genomics. 2016 Aug 23;17(1):670. doi: 10.1186/s12864-016-2941-6 (PMC4994206; doi:10.1186/s12864-016-2941-6)
Supplement: Additional file 15: — Supporting methods. (DOCX 18 kb) [file 12864_2016_2941_MOESM15_ESM.docx]

**Additional file 15: Supporting material and methods.**

**Growth conditions and nucleic acid manipulation**

Conidia were taken from long term storage and plated on PDA plates. For isolates 2548, 7555 and 2516 (HiSeq sequencing), young mycelium was picked from the edges of the grown plates, and cultured in liquid GPY (glucose peptone yeast, 10 g/l glucose; 1 g/l yeast extract; 1 g/l peptone, Oxoid, Belgium) medium for 5 days with 100rpm shaking at room temperature and 16h light/8h dark regime. Mycelium was collected, lyophilized and pulverized in liquid nitrogen with a pestle. DNA was extracted with the Invisorb Spin Plant Mini kit (Invitek) according to the manufacturer’s instruction. Mycelium from isolate bfb0173 was inoculated in 100ml potato dextrose broth and was kept stationary for six days at room temperature and 16h light/8h dark regime. Mycelium has harvested, lyophilized and pulverized by beadbeating with 3.2mm chrome-steel beads at 2500rpm for 20 seconds in a Hybaid Ribolyser. DNA was extracted with the Wizard Magnetic DNA Purification System for Food (Promega) according to the manufacturer’s instructions. For SMRT sequencing, DNA was isolated from isolate 2516. After growth in stationary liquid GPY medium, other conditions as described above, mycelium was immediately crushed in liquid nitrogen with a pestle. Genomic DNA was extracted with the Wizard Magnetic DNA Purification System for Food (Promega) according to the manufacturer’s instructions.

Two different RNA samples were prepared from isolate 2516 for RNA sequencing, one favoring primary metabolism and one favoring “secondary metabolism”. For all treatments, conidia were prepared by inoculating young mycelium on PDA plates and incubating them for 7 days under conidiation inducing conditions (UV 10 hours / dark 14 hours at room temperature). Spores were harvested with Tween20 (0.01%) and gentle disruption with a spatula. After filtration through Miracloth (Merck), concentrations were determined with a Bürker chamber. For each of the conditions described below, RNA was extracted from two replicates and pooled. All replicates were performed at 100 rpm shaking, room temperature and 16h light/8h dark regime. For the primary metabolism sample, 10^6^ conidia were inoculated in 250ml complete medium (CM) ([1](#_ENREF_1)) and incubated for 30h. For the “secondary metabolism” sample, extracted RNA for sequencing was pooled from RNA extracts from five different conditions: trichothecene biosynthesis induction, fungicide application, N starvation, C starvation, and conidiation under UV (each comprising RNA from two replicates).

Trichothecene biosynthesis inducing medium consisted of basal medium ([1](#_ENREF_1)) without agar, but amended with L-arginine at 5mM ([2](#_ENREF_2)), and was inoculated with 10^6^ conidia in 250 ml for 48 hours. For prothioconazole application, N-starvation and C-starvation, 10^6^ spores were inoculated in 250ml CM, and after 12 hours the mycelia were harvested on sterile filter paper, flushed with sterile water, and transferred to erlenmeyers containing 250 ml of the stress inducing media for another 12 hours. Fungicide containing medium consisted of CM amended with 3 ppm of prothioconazole. N-starvation medium consisted of pure basal medium without added N-source, while for C-starvation NaNO_3_ was included in this medium, and sucrose was dropped.

RNA was extracted with TRIzol (Life Technologies). Subsequently, the crude RNA was purified with the RNA cleanup protocol included in the RNeasy Plant Mini kit (Qiagen) according to the manufacturer’s instructions. Quantity of DNA/RNA were assessed with respectively Picogreen or Ribogreen (Life Technologies).

**HiSeq library preparation and sequencing**

DNA from isolate bfb0173 was used for random sheared shotgun library preparation using the NEXTflex ChIP-seq Library prep kit with adaptations for low gDNA input according to the manufacturer’s instructions (Bioscientific). In short, approximately 10 ng DNA was used for fragmentation in a 50 µL volume using a Covaris E210 device targeting 400-500 bp fragment sizes. From the fragmented DNA, 40µL was used for end repair at 22°C for 30 minutes with the enzyme provided by the manufacturer. End repaired DNA was purified using the purification column provided and eluted in 16 µL elution buffer. 3’ adenylation and barcoded adapter ligation was done according to the manufacturer’s instructions (Bioscientific). For all temperature incubation steps a 2720 Thermocycler (Life Technologies) was used. Adapter ligated fragments were then purified twice using 1 volume Ampure XP beads (Agencourt) and finally used in a 50 µL PCR reaction with 15 cycles for library amplification. Amplified libraries were purified using AmpureXP beads and eluted in 20uL. Final libraries were quantified by Qubit (Life Technologies) and Bioanalyzer High Sensitivity DNA assay (Agilent technologies). The library was loaded as (part of) one lane of an Illumina Paired End flowcell for cluster generation using a cBot. Sequencing was performed on an Illumina HiSeq2000 instrument using 101, 7, 101 flow cycles for forward, index and reverse reads respectively. De-multiplexing of resulting data was carried out using Casava 1.8.

For isolates 2516, 2548 and 7555, shotgun libraries were made using the Illumina TruSeq LT DNA sample prep kit according to the manufacturer’s instructions (Illumina). Libraries were quantified by Qubit fluorescence and library fragment size was analyzed by Bioanalyzer High Sensitivity DNA assay. Libraries were then pooled equimolarly and loaded on one flowcell lane for 2x100 nt paired end sequencing on an Illumina HiSeq2000 platform as described above.

**RNAseq library preparation and sequencing**

Four total RNA samples were used for RNAseq library preparation using the Illumina TruSeq total RNA sample preparation kit and guidelines. From 1 µg of total RNA, mRNA was isolated using oligo dT beads, fragmented thermochemically and used for first and second strand cDNA synthesis by random priming. cDNA fragments were (end) repaired, purified using AmpureXP, 3’ adenylated and used for adapter ligation. Adapter ligated cDNA fragments were purified using AmpureXP beads and enriched by PCR amplification using 15 cycles. Final libraries were quantified by Qubit (Life Technologies) and fragment size distribution was determined by Bioanalyzer RNA6000 pico DNA assay (Agilent technologies). These libraries were pooled equimolarly and loaded on one Illumina HiSeq2000 flowcell lane for 2x100 nt paired end sequencing as described above.

**SMRT library preparation and sequencing**

Twenty µg DNA was used for a large (10 kb) library prep according to the manufacturer’s instructions (Pacific Biosciences) with small adaptations. For all bead purifications, siliconized tubes (Sigma) and a Labquake rotator were used to homogenize DNA bead solutions and DNA was eluted for at least 30 minutes. In short, DNA was sheared using a Covaris g-tube by centrifugation two times for 1 minute at 6000 rpm. Sheared DNA was purified and concentrated using 0.45 x washed AmpureXP beads. Fragmented DNA was analysed on a Bioanalyzer 12000 DNA chip (Agilent technologies). DNA damage repair and polishing was done according to the provided protocol. Using the enzyme provided in library prep kit, adapters were ligated over night at 25˚C, followed by heat inactivation of the enzyme at 65˚C for 10 minutes. Subsequently, ligated DNA fragments were treated with ExoIII and ExoVII to remove linear DNA molecules and finally SMRT bells were purified with Ampure XP beads. SMRT bells were quantified using Qubit (Life Technologies) and Bioanalyzer 12000 DNA chip.

SMRT bells were size selected on a Bluepippin High-Pass v3 cassette (Sage science) with a 7000 bp minimum cutoff. After elution, DNA was left for 45 minutes in the elution port before collection and the elution port was washed once to increase the yield of recovered DNA. Size selected SMRT bells were finally purified with AmpureXP beads and eluted in 10 µL, and part was quantified and analysed on a Bioanalyzer 12000 DNA assay.

Size selected purified SMRT bells were used for primer binding and subsequent P6 polymerase complexing in long term storage buffer for four SMRT cells according to PacBio’s binding calculator version 2.3.0.0. Complex Magbead binding was done for 45 minutes at 4˚C and finally used for a 0.05 nM on plate loading concentration on a Pacbio RS II system. Sequencing was done using one cell per well, C4 chemistry and 240 minutes movie time. Magbead binding complexes for an addition twelve SMRT cells were prepared for 0.12 nM on plate loading and sequencing, with same settings as described.

**References**

1. Correll JC, Klittich CJR, Leslie JF. Nitrate non-utilizing mutants of *Fusarium oxysporum* and their use in vegetative compatibility tests. Phytopathology. 1987;77(12):1640-6.

2. Gardiner DM, Kazan K, Manners JM. Nutrient profiling reveals potent inducers of trichothecene biosynthesis in *Fusarium graminearum*. Fungal Genetics and Biology. 2009;46(8):604-13.
