# Supplementary material for: Living apart together: crosstalk between the core and supernumerary genomes in a fungal plant pathogen
Source: BMC Genomics. 2016 Aug 23;17(1):670. doi: 10.1186/s12864-016-2941-6 (PMC4994206; doi:10.1186/s12864-016-2941-6)
Supplement: Additional file 16: — Strategy for the detection of the supernumerary sequence insertions in chromosome three. A: Gel electrophoresis and PCR schematic for insertion 1 at 115-319 kb of chromosome 3. A single amplicon of 1010 bp is formed in the absence of the insertion (primers INS1-FLANK-fwd + INS1-FLANK-rev). When the insertion is present, two amplicons of 848 bp (INS1-FLANK-fwd + INS1-BLOCK-rev) and 1158 bp (INS1-BLOCK-fwd + INS1-FLANK-rev) are formed. An impression of the PCR schematic is given, the principle is the same for both insertions. B: Gel electrophoresis for insertion 2 at 883-1.348 kb of chromosome 3. A single amplicon of 1213 bp is formed in the absence of the insertion (primers INS2-FLANK-fwd + INS2-FLANK-rev). When the insertion is present, two amplicons of 932 bp (INS2-FLANK-fwd + INS2-BLOCK-rev) and 1294 bp (INS2-BLOCK-fwd + INS2-FLANK-rev) are formed. C: Gel electrophoresis of MAT1-1 (1203 bp) and MAT1-2 (859) diagnostic PCR. (DOCX 176 kb) [file 12864_2016_2941_MOESM16_ESM.docx]

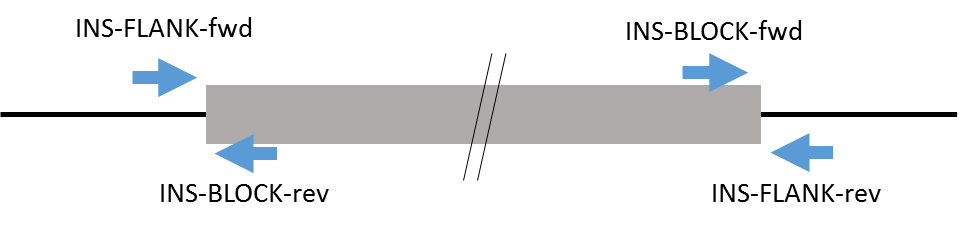

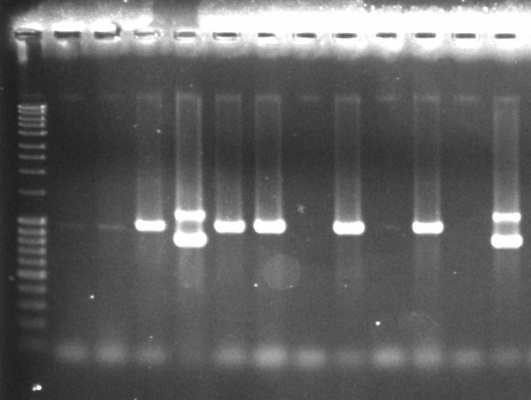


**A**


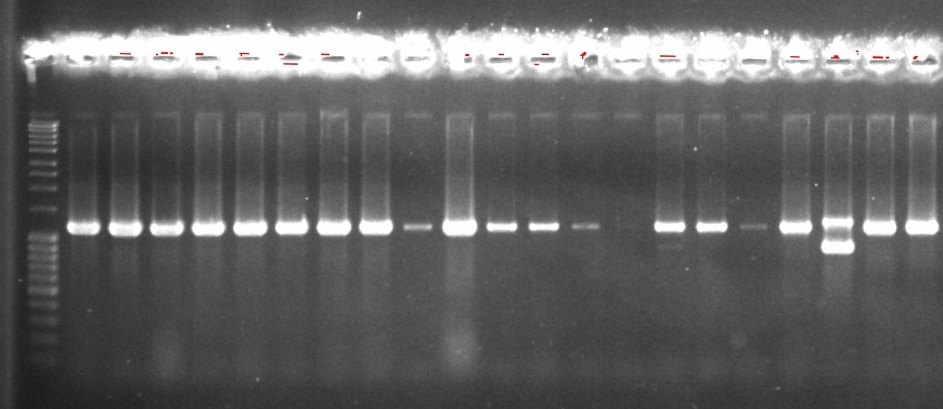


**B**


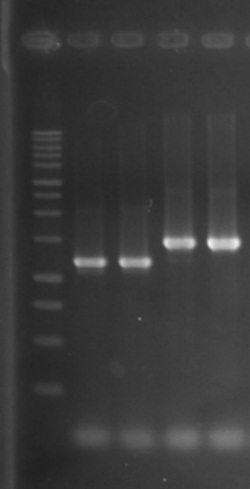


**C**

**Additional file 16** - **Strategy for the detection of the supernumerary sequence insertions in chromosome three.** A: Gel electrophoresis and PCR schematic for insertion 1 at 115-319kb of chromosome 3. A single amplicon of 1010 bp is formed in the absence of the insertion (primers INS1-FLANK-fwd + INS1-FLANK-rev). When the insertion is present, two amplicons of 848 bp (INS1-FLANK-fwd + INS1-BLOCK-rev) and 1158 bp (INS1-BLOCK-fwd + INS1-FLANK-rev) are formed. An impression of the PCR schematic is given, the principle is the same for both insertions. B: Gel electrophoresis for insertion 2 at 883-1.348 kb of chromosome 3. A single amplicon of 1213 bp is formed in the absence of the insertion (primers INS2-FLANK-fwd + INS2-FLANK-rev). When the insertion is present, two amplicons of 932 bp (INS2-FLANK-fwd + INS2-BLOCK-rev) and 1294 bp (INS2-BLOCK-fwd + INS2-FLANK-rev) are formed. C: Gel electrophoresis of MAT1-1 (1203 bp) and MAT1-2 (859) diagnostic PCR.
