# Supplementary material for: Living apart together: crosstalk between the core and supernumerary genomes in a fungal plant pathogen
Source: BMC Genomics. 2016 Aug 23;17(1):670. doi: 10.1186/s12864-016-2941-6 (PMC4994206; doi:10.1186/s12864-016-2941-6)
Supplement: Additional file 18: — Centromeres of the four core chromosomes of isolate 2516. Hypothetical position of the centromeres of the four core chromosomes in F. poae isolate 2516 (denoted with black line). The positions of these regions that are low in GC%, coincide with low GC% regions in F. graminearum, where the presumed centromeres lie for that species. (DOCX 302 kb) [file 12864_2016_2941_MOESM18_ESM.docx]

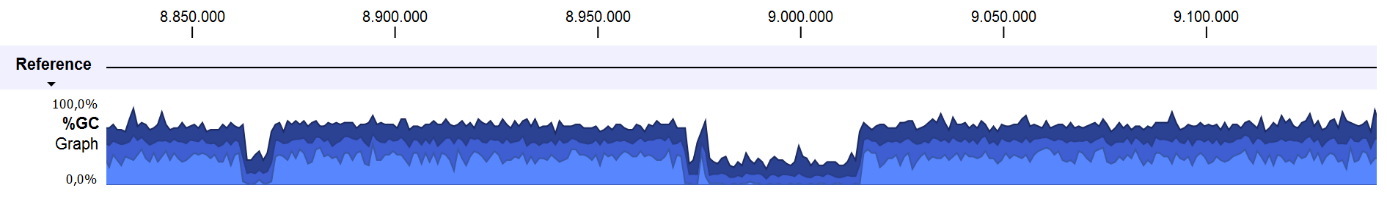


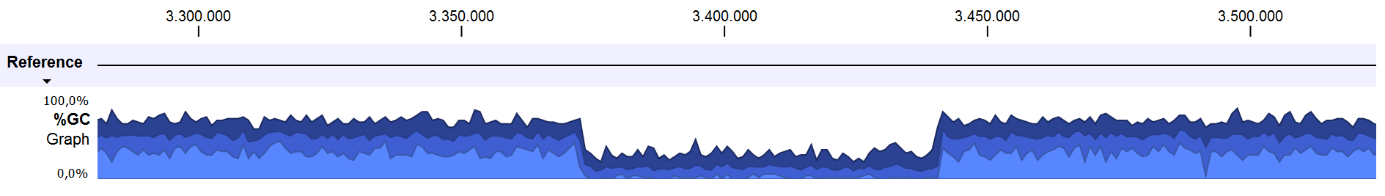


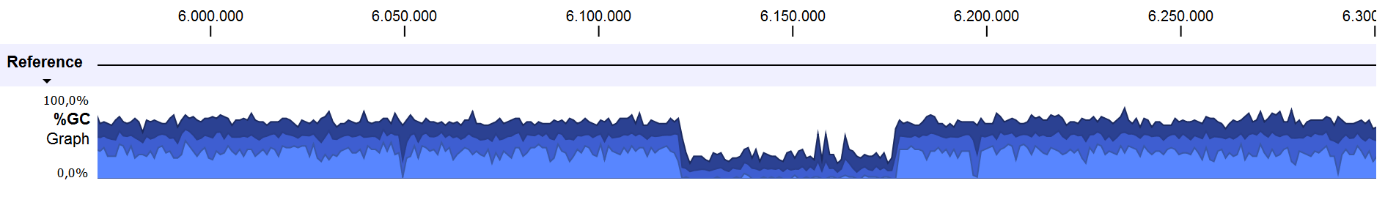


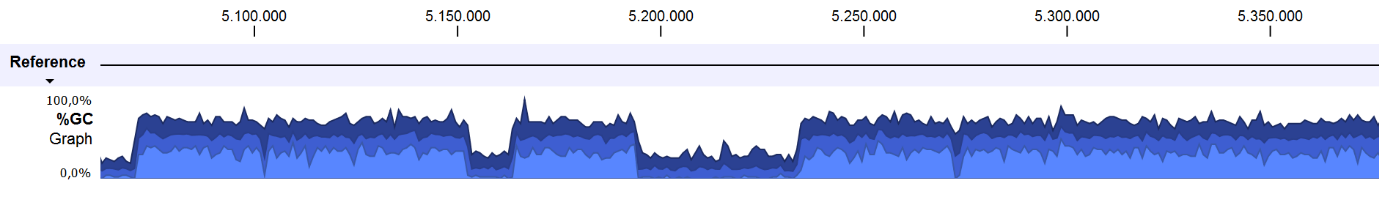


**Additional file 18** – **Centromeres of the four core chromosomes of isolate 2516.** The positions of these regions that are low in GC%, coincide with low GC% regions in *F. graminearum*, where the presumed centromeres lie for that species.
