# Supplementary material for: Living apart together: crosstalk between the core and supernumerary genomes in a fungal plant pathogen
Source: BMC Genomics. 2016 Aug 23;17(1):670. doi: 10.1186/s12864-016-2941-6 (PMC4994206; doi:10.1186/s12864-016-2941-6)
Supplement: Additional file 19: — Molecular dating of TEs on the supernumerary genome, with outliers. Copies were aligned and branch lengths extracted from a maximum-likelihood phylogenetic tree. Branch lengths were used to calculate divergence times with a fixed substitution rate (1.05 * 10-9 substitutions per site per year). (DOCX 1068 kb) [file 12864_2016_2941_MOESM19_ESM.docx]

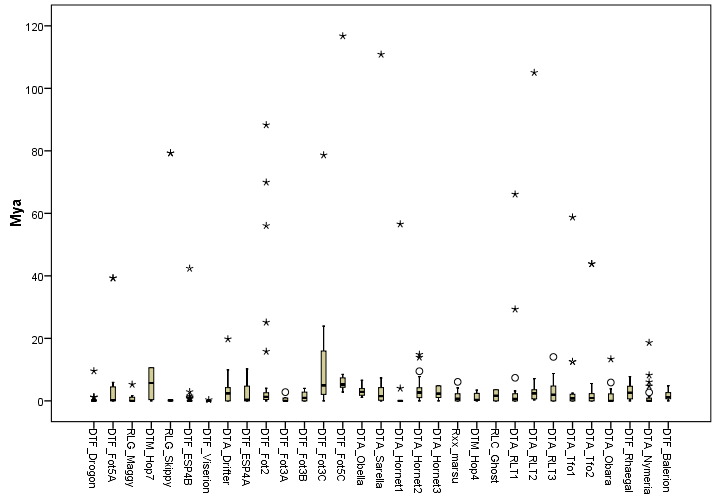


**Additional file 19** - **Molecular dating of TEs on the supernumerary genome, with outliers.** Divergence estimation of intact (not RIPped) TE copies on the supernumerary (right) genome. Copies were aligned and branch lengths extracted from a maximum-likelihood phylogenetic tree. Branch lengths were used to calculate divergence times with a fixed substitution rate (1.05 * 10^-9^ substitutions per site per year ([39](#_ENREF_39))).
